# G Protein-Coupled Receptor Systems as Crucial Regulators of DNA Damage Response Processes

**DOI:** 10.3390/ijms19102919

**Published:** 2018-09-26

**Authors:** Hanne Leysen, Jaana van Gastel, Jhana O. Hendrickx, Paula Santos-Otte, Bronwen Martin, Stuart Maudsley

**Affiliations:** 1 Department of Biomedical Sciences, University of Antwerp, 2610 Antwerp, Belgium; Hanne.Leysen@student.uantwerpen.be (H.L.); Jaana.vanGastel@uantwerpen.vib.be (J.v.G.); Jhana.Hendrickx@uantwerpen.vib.be (J.O.H.); Bronwen.Martin@uantwerpen.be (B.M.); 2 Translational Neurobiology Group, Center of Molecular Neurology, VIB, 2610 Antwerp, Belgium; 3 Institute of Biophysics, Humboldt-Universität zu Berlin, 10115 Berlin, Germany; p.santosotte@gmail.com

**Keywords:** G protein-coupled receptor (GPCR), aging, DNA damage, β-arrestin, G protein-coupled receptor kinase (GRK), interactome, G protein-coupled receptor kinase interacting protein 2 (GIT2), ataxia telangiectasia mutated (ATM), clock proteins, energy metabolism

## Abstract

G protein-coupled receptors (GPCRs) and their associated proteins represent one of the most diverse cellular signaling systems involved in both physiological and pathophysiological processes. Aging represents perhaps the most complex biological process in humans and involves a progressive degradation of systemic integrity and physiological resilience. This is in part mediated by age-related aberrations in energy metabolism, mitochondrial function, protein folding and sorting, inflammatory activity and genomic stability. Indeed, an increased rate of unrepaired DNA damage is considered to be one of the ‘hallmarks’ of aging. Over the last two decades our appreciation of the complexity of GPCR signaling systems has expanded their functional signaling repertoire. One such example of this is the incipient role of GPCRs and GPCR-interacting proteins in DNA damage and repair mechanisms. Emerging data now suggest that GPCRs could function as stress sensors for intracellular damage, e.g., oxidative stress. Given this role of GPCRs in the DNA damage response process, coupled to the effective history of drug targeting of these receptors, this suggests that one important future activity of GPCR therapeutics is the rational control of DNA damage repair systems.

## 1. Introduction

With the knowledge gained about mechanisms underlying health and disease, as well as improved living standards and sanitization, there has been a major increase in the global average lifespan [1]. The world health organization reported in 2015 that an estimated 900 million people were aged 60 or older. By 2050, this number is expected to increase to about two billion people [2]. Despite this positive result of improved healthcare, a major complication incurred with this increase in the size of the worldwide elderly population is the burgeoning prevalence of aging-related diseases including neurodegenerative disorders, cardiovascular diseases and diabetes mellitus [3]. Indeed, this has been borne out through multiple studies connecting age-related molecular pathologies and the incidence of these disorders [4,5,6]. These studies suggest that the aging process is an underlying cause for multiple diseases; however, aging itself is not considered a disorder, but a normal physiological process [3]. Pathological aging can be defined as a progressive deterioration of physiological functions, which will eventually lead to systemic dysfunction and death [3]. These alterations include metabolic dysfunction, genome instability, telomere attrition and oxidative stress [7,8]. A greater understanding of these processes should improve our capacity to prevent or treat age-related diseases [9].

Transmembrane heptahelical GPCRs represent perhaps the most studied and effective drug targets to date. Their near ubiquitous role in physiological processes, coupled to their capacity to recognize a wide diversity of impinging molecules, makes them ideal targets for pharmacotherapeutic design [10,11]. As a testament to the functional efficacy of targeting GPCRs in disease, 475 drugs (~34% of all drugs approved by the FDA, acting on over 108 unique GPCR targets) are currently clinically employed [11]. While currently dominating the realm of therapeutics, there is still a strong impetus for future GPCR-based drug design. There are over 300 new experimental drugs that are currently in clinical trials, of which ~20% target 66 previously unexploited GPCR systems. The major disease indications for GPCR modulators have shown a trend towards diabetes, obesity and Alzheimer disease (AD), all of which are strongly age-dependent disorders. While the majority of the worldwide drug design effort has been made using the concept of exploiting and controlling the G protein-dependent signaling modality of GPCRs, there is now a growing field of more ‘engineered efficacy’ therapeutics that can utilize alternative modes of non-G protein-mediated GPCR signaling [12,13,14]. The emergence of these new and diverse GPCR signaling modes expands our concepts of the types of signaling systems that can be controlled through GPCR modulation. In this review, we will investigate one of these new target systems that may hold the key to the future treatment of multiple age-related disorders [15,16,17,18], i.e., the DNA damage-response (DDR) system.

As life proceeds through the individual’s aging process, both endogenous (e.g., reactive oxygen species (ROS)) and environmental (e.g., ionizing radiation) stressors are constantly attacking DNA, causing structural damage [9]. Unrepaired DNA damage negatively affects genome replication and transcription, causing wide-scale chromosomal aberrations that disrupt critical cell functions such as energy metabolism and protein folding/management [19,20,21]. Given the importance of DNA-protective activity as an anti-aging strategy, coupled to the feasibility of GPCR druggability, the generation of GPCR-based DDR controlling agents holds considerable promise for improved treatments for both disorders of genomic aging such as Werner syndrome or ataxia-telangiectasia, as well as age-related disorders such as metabolic syndrome or Parkinson’s disease.

## 2. Aging, Metabolic Functionality, DNA Stability, Damage and Repair

### 2.1. Metabolic Dysfunction, Oxidative Damage and Aging

Systemic aging in humans is strongly associated with the accumulation of deleterious molecular perturbations that negatively affect the functionality of almost all cells, tissues and organs. This progressive and stochastic accumulation of molecular perturbations induces significant cellular signaling dysfunctions that affect multiple processes related to energy metabolism, cell survival, genomic instability (via sub-optimal damage responsivity and repair efficiency) and aberrant cellular replication.

The molecular control of the aging process has long been associated with the highly-conserved insulinotropic receptor signaling system. This metabolic system controls the effective uptake and metabolism of glucose as the primary energy source in the majority of higher organisms. The crucial role of this signaling system in aging has been evidenced by the demonstration of lifespan extension, in species ranging from nematodes to mice, by mutations affecting insulin receptor signaling [22,23,24,25,26]. These mutations affect several cellular functions that are negatively regulated by the insulin receptor and therefore typically observed under fasting conditions where little caloric intake was extant and a likelihood of insulin resistance was low. Concomitant with this, lifespan as well as ‘healthspan’ (i.e., period of life in which no overt pathophysiology is extant) extensions have also been induced by caloric restriction and intermittent fasting interventions [6,27,28]. Considering this evidence, the insulinotropic system represents perhaps the most critical system in organismal development and survival. This primacy is due to this system’s ability to generate the optimal adenosine triphosphate (ATP) yield from catabolized dietary carbohydrate sources. As with all biological systems, the perfect repetition of its function is subject to incremental failure and reduction of sensitivity over time, i.e., the age-dependent inevitability of ‘insulin resistance’ and ‘metabolic syndrome’ receptor systems [29,30]. Therefore, with increasing age, there is a prevalent system-wide reduction in the ability of the body to cope with stress, in part, due to a degradation of the efficiency of energy-generating (e.g., ATP) metabolic systems [29,31,32]. Disruption of the primary energy-synthesizing system, i.e., mitochondrial oxidative phosphorylation, leads to both ATP depletion (thus affecting electrical cellular excitability, proteolytic activities, transmembrane transport processes and kinase activity), as well as an increase in the deleterious effects of unregulated hyperglycemia, e.g., systemic inflammation, enhanced ROS generation, arterial stenosis, impaired tissue healing, neuronal damage and renal failure [33,34]. Hence, many characteristic factors of the aging process are linked to effective energy management, i.e., the generation of insulin resistance, disruptions to oxidative phosphorylation of glucose and changes in body fat composition [29].

### 2.2. Oxidative Aging and DNA Damage Responses

The inexorable generation of systemic metabolic dysfunction (linked to insulinotropic system aberration) induces a global imbalance between ROS and endogenous antioxidant pathways. This systemic perturbation results in an increased susceptibility of lipids, proteins and nucleic acids to oxidative radical attack and the creation of sustained oxidative damage [35]. The Harman free radical/oxidative stress theory stipulates that physiological iron and other metals in the body would cause ROS accumulation in cells as a by-product of normal redox reactions. ROS are natural signaling entities, generated as a by-product of a variety of pathways involved in aerobic metabolism [36]. ROS-mediated oxidative stress in turn causes DNA and cellular damage in aged cells and organisms, which could trigger cellular apoptosis [35,36,37]. Depending on the source of damage, DNA can be altered in different ways, including nucleotide alterations (mutation, substitution, deletion and insertion) and the creation of bulky adducts, single-strand breaks (SSBs) and double-strand breaks (DSBs) [38]. To guide accurate repair of these lesions, cells activate a highly nuanced signaling network (i.e., the DDR pathway) that: (i) detects the presence of DNA damage sites; (ii) transmits the detection of damage to coordinating signal transducers; (iii) stimulates the activation of cell-cycle checkpoint and DNA damage repair mechanisms [39,40,41]. There are currently four elucidated DDR mechanisms characterized in mammalian cells: base excision repair (BER); nucleotide excision repair (NER); homologous recombination (HR); and non-homologous end-joining (NHEJ) [9,42]. BER mainly corrects single lesions or small alterations of bases caused by ROS [42,43]. This pathway involves multiple steps, starting with recognition of the damaged DNA by a DNA glycosylase [44], followed by the activation of a pathway common to SSB repair, involving an apurinic/apyrimidinic (AP) endonuclease to generate the DNA 3′OH terminus [45]. The final repair steps involve a synthesis stage with a DNA polymerase, followed by sealing the DNA lesion via DNA ligase activity [45]. NER is a more complex process for removing bulky DNA lesions formed by exposure to radiation, chemicals or through protein-DNA adduct formation [42,44]. DSB repair is performed by either HR or NHEJ [42]. DSBs caused by exogenous stressors can be repaired by either of these pathways [46]. Damage produced by a malfunction of DNA replication forks is primarily, or even exclusively, repaired by HR [47]. HR-dependent DSB repair is initiated by forming 3′OH overhangs, which associate with Rad52 and subsequently with polymerized Rad51 [48]. NHEJ is initiated by the recognition and binding of the Ku heterodimer (Ku70 and Ku80) to the DSB [49,50]. This then serves as a scaffold to recruit other NHEJ factors to the damaged site, such as the DNA-dependent protein kinase (DNA-PKs) [50].

Stress-induced DNA damage is a routine process in cells; this damage can occur at the level of whole chromosome structures, as well as to exposed single- or double-strand entities. Chromosomal DNA stability is provided by nucleoprotein-DNA structures termed telomeres [51]. Mammalian telomeres are repetitive DNA sequences, which form a lariat-like structure by associating with the multimeric Shelterin protein complex (also known as the telosome) to shield the exposed ends of chromosomal DNA from damage [51,52,53]. Telomeres shorten progressively with each cell replication cycle [54], thus imposing a functional limit on the number of times a cell can safely divide. Significantly shortened telomeres trigger cellular senescence in normal cells, or genomic instability in pre-malignant cells, which contribute to numerous degenerative and aging-related diseases [55]. Multiple lines of research, from human, murine and in cellulo studies, have shown that oxidative stress is associated with accelerated telomere shortening and dysfunction [56,57,58,59,60,61,62,63]. Several mechanistic models have been proposed to explain how oxidative stress accelerates telomere shortening. One possibility is that oxidative stress triggers cell death and/or senescence, and as a compensation, the extant cells then undergo further recuperative divisions, leading to increased telomere shortening [55]. Another widely-appreciated model hypothesizes that ROS induce SSBs at telomeres directly, or as intermediates in lesion repair, leading to replication fork collapse and telomere loss [64].

Furthering the associations between metabolic dysfunction, aberrant DDR and advanced aging phenotypes, several classical DDR-associated diseases (Hutchinson-Gilford progeria, Werner and Cockayne syndromes and ataxia-telangiectasia) are linked to dysglycemic states and insulin resistance [65,66,67,68,69,70]. Given the strong linkage between insulinotropic decline, oxidative stress, DNA damage and advanced aging, it is clear that molecular interventions that are able to manipulate this signaling convergence beneficially may represent important future treatments for age-related diseases.

### 2.3. Metabolic-Clock Process Linked with DDR

It has recently been demonstrated that the cellular clock and circadian rhythm are disrupted in the aging process [71]. Circadian clock rhythms, present both within the whole-organism and at the single-cell level, underpin the everyday fluctuations in biochemical, behavioral and physiological functions of organisms [72,73,74]. These circadian signaling systems allow the organism to reliably repeat daily patterns of activity throughout its lifespan [75]. The daily rhythm of mammalian energy metabolism is also subject to the circadian clock system. So-called ‘clock genes’ (factors that constitute biological clock regulation) have been revealed not only to constitute the molecular clock of cells, but also to function as facilitators that regulate and interconnect circadian and metabolic functions. As circadian signals generated by clock genes regulate metabolic rhythms, it is therefore unsurprising that clock gene function is tightly coupled to glucose and lipid metabolism. Clock gene dysfunction has thus also been strongly associated with metabolic disorders including diabetes and obesity [76,77,78,79]. Changes in energy balance, in turn, conversely affect circadian clock functionality [80,81,82]. Recent research has demonstrated that the application of high-fat diets to mice increases the circadian period of their locomotor activity under constant dark conditions, suggesting molecular disruption to their suprachiasmatic nucleus clock that controls global somatic time measurement [83]. In addition, high-fat diet supplementation has been shown to disrupt the rhythmic expression of clock genes in peripheral tissues [84]. Alterations in temporal feeding patterns have also been shown to affect circadian clock gene activity in energy-regulatory peripheral tissues [85]. As we have described previously (Section 2.1), dysfunctional metabolic activity may be one of the prime triggers of the pathological aging process that is then associated with telomeric instability and DNA damage. To this end, it is unsurprising that clock gene factors can control and integrate metabolic sensation, day-to-day age assessment and DNA stability. Thus, components of circadian clock, such as Aryl hydrocarbon receptor nuclear translocator-like protein 1 (BMAL1-CLOCK), period circadian protein homolog 1 (PER1), period circadian protein homolog 2 (PER2), period circadian protein homolog 3 (PER3) and inactive tyrosine-protein kinase transmembrane receptor ROR (ROR1), are suggested to be involved in cellular response to genotoxic stress [72,86,87,88,89]. As cellular clocks not only regulate chronological aging, but also the rate/extent of metabolic dysfunction, telomere stability and DNA damage [90,91,92], it is unsurprising that clock functionality is now linked to many age-related disorders, e.g., dementia [93,94], glycemic/adiposity disorders [95] and premature aging diseases associated with attenuated DDR [65,66,67,68,69,70,96,97]. Therapies targeting clock regulation mechanisms have thus demonstrated promising effects on the treatment of aging-related diseases including metabolic syndrome and psycho-affective disorders [98,99,100].

In these initial sections (1 and 2), we have outlined how the seemingly impenetrably complex process of aging, with its strong association with DDR events, may be more effectively understood using signaling network-based concepts. Forming the first level of synergy between the GPCR and DDR systems, we have also detailed how the currently expanding range of GPCR signaling modalities also seems to operate at a network level. At the second level of GPCR-DDR synergy, we have also demonstrated that both of these systems interconnect via the observed metabolic dysfunctions in the aging process. At a third synergistic level, both GPCR and DDR systems, via the alteration of energy metabolism, conspire to accelerate aging pathologies via accumulated oxidative damage. In the final fourth level of GPCR-DDR synergy, we have shown that these two systems converge via their common roles in both circadian clock and metabolic regulation to create a coherent and pervasive role of GPCR-DDR functionality in the aging process. In the following sections, we shall further refine these observations and illustrate them with specific exemplary findings.

## 3. G Protein-Coupled Receptor Systems: Intersections with DNA Damage and Repair Processes

### 3.1. GPCR Signaling Diversity

The GPCR superfamily represents perhaps the most diverse group of transmembrane proteins in the human proteome [101]. GPCRs have evolved to provide cells with an incredibly nuanced sensory system for entities ranging from photons, small metabolites, chemical neurotransmitters, to complex glycoprotein hormones and exogenous animal toxins [102]. This unparalleled molecular diversity of GPCR sensitivity has allowed molecular pharmacologists to exploit these complex signaling systems rationally to combat a plethora of diseases.

GPCRs provide a simple, but highly flexible, mechanism to facilitate the signal transfer of the ‘message’ of the extracellular stimulator (i.e., the receptor ‘ligand’ in biomedical terms) to the intracellular milieu. Hence, the stimulated receptor entrains characteristic cell signaling cascade responses to generate a productive cellular response to the external input [103]. These versatile heptahelical receptors essentially function as ligand-activated guanine nucleotide exchange factors (GEFs) for heterotrimeric G proteins. G protein activation is initiated through ligand-driven changes in the tertiary structure of the heptahelical core that are then transmitted to the intracellular transmembrane loops and carboxyl terminus of the receptor. These conformational changes alter the ability of the receptor to interact with intracellular G proteins and catalyze the exchange of GDP for GTP on the heterotrimeric G protein α subunit. This nucleotide exchange promotes dissociation of the G protein αβγ subunit heterotrimer, releasing the GTP-bound α subunit and the free βγ subunit. The GTP-bound α subunit stimulates its cognate downstream effectors, e.g., adenylate cyclase or phospholipase C, conveying information about the presence of an extracellular stimulus to the intracellular environment. In addition to the Gα subunit, free βγ subunits also possess effector stimulatory activity, e.g., promotion of G protein-coupled receptor kinase binding to the receptor. This classical ‘G protein-centric’ view of GPCR function still holds true, yet data accumulated over the last decade have suggested that G protein signaling is not the only physiologically-relevant signaling pathway employed by these receptors [104,105,106,107,108]. The discovery of alternative therapeutically-tractable GPCR signaling pathways, such as the β-arrestin signaling pathway, suggests that additional drug design avenues may be fruitful. Luttrell et al. first demonstrated that β-arrestins interact with Src family kinases and couple beta adrenergic receptors to extracellular signal-regulated kinase 1/2 (ERK1/2) pathways [12]. β-arrestin molecules were primarily associated with GPCR internalization and degradation [12,109]. However, in addition to mediating endocytosis of GPCRs, β-arrestins have been demonstrated to scaffold a wide variety of signaling complexes associated with GPCR signaling cascades that can occur in parallel, or subsequent to, G protein turnover [104]. β-arrestins have subsequently been demonstrated to bind a wide variety of kinases, e.g., E3 ubiquitin ligases, phosphodiesterases and transcription factors [110]. More recently, it has been shown that activation of β-arrestin, through the β_2_-adrenergic receptor (β_2_AR), leads to increased DNA damage, p53 degradation and the promotion of apoptosis [111,112]. These data suggest that if the activation of β_2_AR could be biased to signal through a more ‘non-β-arrestin’ signaling mode, DNA damage repair could be promoted. Implicit with the additional complexity of GPCR signaling repertoires, it has been demonstrated that these additional GPCR transduction mechanisms are facilitated and specified by the creation of stable multiprotein complexes with the receptor [103,107]. These large multi-protein complexes likely represent highly stable, due to the need to regulate multiple protein-protein interactions, sub-structures that are often termed ‘receptorsomes’. Given the likely presence of both G protein and non-G protein GPCR signaling, it is likely that cellular responses to stimulatory ligands will comprise a range of signaling outcomes dictated by both G protein activity and the expression profile of additional proteins that help create stable receptorsome complexes.

In addition to the recent introduction of non-G protein signaling to the functional repertoire of GPCR activity, new theories associated with the enlarged variety and cellular spatial nature of receptor activity are redefining our future concepts of therapeutic development. From their initial discovery, GPCRs were classically considered to be only ligand responsive when expressed on the cell surface plasma membrane. In contrast to this plasma membrane expression, a large majority of the total cellular amount of receptor protein was thought to be held in a cytosolic ‘reserve’ as nascent GPCRs ready to replenish the ‘actively signaling’ plasma membrane forms. This classical view of receptor pharmacology is still valid, especially for rapid extracellular stimulator-based G protein activation. There is now considerable evidence however demonstrating that GPCRs can also signal from intracellular membranes such as endosomes, mitochondria, endoplasmic reticulum, Golgi apparatus and the nucleus [113,114]. This additional signaling capacity suggests that GPCRs also act as intracellular signal transducers for stimulatory factors generated inside the cell. With this concept in mind, it is thus feasible to propose that GPCRs can also act as sensors, at the molecular level, for agents that can directly or indirectly induce oxidative stress and/or DNA damage.

### 3.2. GPCR Functionality in the Context of Molecular Gerontology

A considerable proportion of the global mechanistic process of aging is driven by a degradation of metabolic function resulting in elevated oxidative stress and DNA damage. In recent years, it has been demonstrated that there is a complex neuroendocrine control network of inter-connected GPCR systems that regulate ‘neurometabolic’ activity. This convergence of GPCR-based systems bridges the functional domains of endocrine and neuronal systems in health and disease [115,116,117,118,119,120]. Here, we also posit that in addition to controlling the aging process via regulation of global metabolism, GPCR systems can also exert a trophic effect on DDR during normal and pathological aging.

Metabolically-driven aging is characterized by the accumulation of adverse changes in cells over time that attenuates global homeostatic energy control and augments the risk of developing nearly all diseases [121]. In addition to cellular/tissue damage caused by accumulated protein/DNA damage, molecular aging ‘programs’ (i.e., coherent and repeated pathological patterns of protein expression leading to stress-related damage) can also generate age-related increases in cellular senescence. Cell growth arrest and hyporesponsiveness to extrinsic stimuli via cell surface receptors, such as GPCRs, are hallmarks of senescent cells [122,123,124,125]. Cell senescence describes the process in which cells cease dividing, but do not enter an apoptotic state. These senescent cells possess distinct functional phenotypes, compared to normal cells, with respect to chromatin remodeling and protein secretory behavior [126,127,128]. The discovery of this ‘cell stasis’ process has been attributed to Hayflick and Moorhead [129] after they observed the phenomenon of the irreversible growth arrest of human diploid cell strains induced by extensive serial passaging in culture. This ‘replicative senescence’ is linked with telomeric degradation following each cell cycle. As we have discussed previously, this telomere attenuation [129,130] is strongly associated with DNA frailty. Rather than representing a functional ‘dead end’ of cell physiology, evidence gathered over recent years has demonstrated the importance of senescence-related signaling in processes such as embryonic development [131], wound healing/repair [132,133] and, most importantly, aging [134,135].

In addition to telomeric degradation, additional stressors have been shown to engender cellular senescence, e.g., certain DNA lesions and ROS attack [136,137], both of which are linked through the DDR signaling pathway. It is thought that senescence can be regulated via ATM or ATR (ataxia Telangiectasia Rad3 related) kinases that effectively block cell-cycle progression through the stabilization of p53 and transcriptional activation of the cyclin-dependent kinase (Cdk) inhibitor p21 [138]. Along with cell cycle arrest, the alteration of the functional cellular ‘secretome’ (i.e., the range of secreted proteins from a specific cell type) of the specific cell entering a senescent state is one of the characteristic features of this aging-associated state [139]. Profound chromatin remodeling represents one of the first steps in age-related senescence; this event causes a coherent cellular response involving elevation of transcript levels for pro-inflammatory cytokines, chemokines, cell-remodeling growth factors and proteases [140,141]. This modulatory secretory phenotype has now been codified as the senescence-associated secretory phenotype (SASP) [139,142,143]. SASP responses, like cell cycle arrest events, can also be dependent on protracted DNA damage signaling [143], caused by the feed-forward loops that can be generated between DDR signaling and ROS attack [144]. Interestingly, it has been demonstrated that SASP-associated activity is also strongly linked to modifications in GPCR functionality [145,146].

### 3.3. GPCR Signaling Systems and DNA Damage Repair

While the aging process and the accumulation of age-related damage seem inevitable facts of metabolic life, the strong involvement of GPCR-associated signaling cascades at many levels of this process provides a potentially important and effective drug-based mechanism for amelioration and/or retardation of this process [106,147,148,149]. Aging, as a molecular process, is clearly a slowly developing entity, coordinated by the interaction of multiple signaling systems across almost all somatic tissues over decades. This complexity makes it a troublesome process to target using conventional ‘monolithic target’ therapies, e.g., the failure of anti-amyloid therapies targeting age-related dementia [150]. In contrast, complex mechanistic disease systems may be more effectively targeted by therapeutics that possess multidimensional pharmacological efficacy profiles [151,152,153,154,155]. The discovery and development of the concept that GPCR systems can effectively target and regulate complex transcriptomic/proteomic responses via receptorsome-based non-G protein-dependent signaling [103] provides a feasible platform upon which multidimensional therapeutic interventions for aging can be created [13,14,106]. In their elegant manuscript, Watts and Strogatz [156] demonstrated that an optimal level of communication between entities, within any specific complex system, is facilitated by a level of organization where some nodes within the network possess a greater degree of regulatory connectivity compared to other nodes. In the case of molecular signaling networks in the aging process, it is likely therefore that some proteins possess more profound network-regulating functions than others [8]. These network-controlling factors have been termed ‘keystones’ or ‘hubs’ and are thought to provide a mechanism of dimensional condensation for highly complex cellular signaling systems. This network organization facilitates the rapid transfer of coherent biological/pathological perturbations across a complex series of nodes by making so-called ‘short cuts’ across the network. As such, the super-complex aging process networks can be controlled at a trophic keystone/hub level rather than by individual sensation/regulation at the individual node (protein or gene) level [157,158]. These keystones therefore likely connect and coordinate multiple discrete signaling cascades that synergize to regulate multifactorial somatic processes. Demonstrating the efficiency of organizing networks in this manner, it has been shown that even networks containing thousands of nodes require only the presence of surprisingly few (5–10) keystones to facilitate rapid transfer across large systems [156]. Targeting these trophic-level proteins, potentially via the recently discovered GPCR-based transcriptomic efficacy role, facilitates regulation of such complex disorders in a rational manner as opposed to the unfeasible proposal of therapeutic aging control at every molecular point in the network. In the next section, we will identify key components of complex GPCR signaling systems that possess strong functional roles in the aging-DDR process; by doing so, we hope to illuminate the potential for effective molecular interventions for neurometabolic aging pathologies.

#### 3.3.1. Heptahelical GPCRs and DNA Damage

##### Lysophosphatidic Acid Receptor

The GPCR heptahelical core still remains the primary target of therapeutic drug development, but it is clear from considerable research that the functionality of this core transmembrane protein is heavily modulated by accessory protein interactions in addition to the standard G protein associations. These accessory protein interactions have been shown to control receptor dimerization, linkage to non-receptor signaling adaptors and associations with other complex receptor systems [110,159,160,161]. The involvement of GPCR signaling systems in DDR pathways has received interest from multiple research groups recently. For example, LPA2 (lysophosphatidic acid G protein coupled receptor subtype 2) receptor stimulation has been shown to activate MAPK/ERK, PI3K/AKT and NF-κβ signaling, which leads to enhanced cells survival and repair of radiation injuries [162,163,164]. The activation of an NF-κβ-dependent, ATM-based signaling cascade in turn then controls the expression of the LPA2 receptor itself [165]. It has furthermore been shown that the activation of this receptor leads to the resolution of radiation-induced γH_2_AX lesions [166] and enhanced long-term survival of acutely-irradiated cells [167].

##### Dopamine D2 Receptor

Protein arginine methylation regulates diverse functions in eukaryotic cells, including gene expression, the DDR and circadian rhythms. Protein arginine methyltransferase 5 (PRMT5) has been shown to interact directly with and effect the methylation of the dopamine D2 receptor (D2R). This would therefore represent a potential new signaling pathway with which novel pharmacological agents could modulate GPCR signaling by changing the methylation status of key cell signaling associated with DDR responses [168]. In addition to this link between D2Rs and pro-aging mechanisms, therapeutic targeting of D2R-associated DNA damage effects may also yield the creation of novel anti-neoplastic agents [169]. The selective DR2 blocker thioridazine has been shown to induce apoptosis and autophagy in ovarian cancer cell lines, which may be attributed to an increased level of ROS with associated DNA damage. Thioridazine treatment also resulted in the augmented expression of various proteins associated with oxidative stress, including nuclear factor E2-related factor 2 (NFE2L2), a pivotal transcriptional factor involved in cellular responses to oxidative stress. Conversely, thioridazine treatment has been shown to reduce expression of heme oxygenase 1, NAPDH quinone dehydrogenase 1, hypoxia inducible factor-1α and phosphorylated protein kinase B (Akt-1), factors that together represent a concerted pro-DNA damage molecular phenotype.

##### CXCR4 Receptor

The chemokine receptor, CXCR4, has been strongly associated with the modulation of DDR-related activity and cell cycle control in the context of oncology. Small peptide antagonists, potentially acting via non-G protein-dependent signaling pathways, have been shown to possess anti-neoplastic activity via the activation of ‘mitotic catastrophe’, an event associated with a premature or inappropriate cellular entry into mitosis [170]. The experimental peptide antagonist (CTCE-9908) has been shown to induce multinucleation, cell cycle arrest and abnormal mitosis through the deregulation of DNA damage and spindle assembly checkpoint proteins. The chemokine receptor CXCR4 and its ligand, CXCL12, are critical factors supporting quiescence and bone marrow retention of hematopoietic stem cells (HSCs) during the aging process. Engineered disruption of CXCR4 receptor expression in mice has been demonstrated to induce an increase in the production of ROS in bone marrow. This elevated ROS activity was subsequently shown to induce apoptosis via enhanced p38 MAPK activation, increase DNA DSBs and apoptosis, leading to a marked reduction in HSC repopulating potential. Taken together, these multiple signaling activities result in an increased rate of bone aging [171].

##### Hydroxycarboxylic Acid (Lactate) Receptor

The primary metabolite lactate was originally considered to be a biomedical waste product of metabolism. Lactate has however been shown to possess important positive signaling roles, especially in the central nervous system (CNS). In the CNS, lactate is released by astrocytes in response to neuronal activation, after which it is taken up by neurons, oxidized to pyruvate and used for synthesizing acetyl-CoA to feed oxidative phosphorylation [172]. The discovery of a cognate GPCR for lactate (hydroxycarboxylic acid) receptor 1 (HCAR1) [173] further reinforced the importance of the lactate system in linking cellular metabolism with cognitive function and neuroprotective activity. The lactate GPCR system has subsequently been demonstrated to mediate in part the beneficial neurocognitive aspects of anti-aging interventions such as exercise [174,175]. HCAR1 activity has been implicated in lactate-related enhancement of DNA repair mechanisms in cells, via regulation of LIG4 (DNA ligase 4), NBS1 (Nijmegen breakage syndrome 1), APTX (aprataxin) and BRCA1 (BRCA1, DNA repair associated) expression, as well as an increase in DNA-PKcs activity [176,177]. In addition to controlling DDR mechanisms, the HCAR1 also appears to control the generation of chemoresistance to the DNA damaging agent doxorubicin, via a reflexive ABCB1 (ATP binding cassette subfamily B member 1) transporter upregulation in HeLa cells [178].

##### Melanocortin 1 Receptor

While a considerable degree of DNA damage can be induced during the aging process via ROS attack, the long-term exposure to solar ultraviolet radiation can also contribute to age-related genomic frailty. Recent research has shown that both melanocortin 1 (MC1R), as well as endothelin B (ENDBR) receptors play important roles in the constitutive regulation of melanocytes and their response to solar ultraviolet radiation [179]. Ligand-mediated activation of the MC1R has been shown to (i) effectively attenuate the extent of damage induced by oxidative stress events and (ii) augment the activity of DNA repair pathways. Specifically, α-MSH (alpha-melanocyte stimulating hormone)-mediated stimulation of MC1R results in the phosphorylation and activation of the DNA damage sensors ATM, ATR and DNA-PK [179,180]. Treatment with α-MSH has also been shown to increase the levels of Chk1 and Chk2 (checkpoint kinase 1 and 2), the immediate downstream targets of ATR and ATM, as well as the transcription factor p53 and γ-H_2_AX, the phosphorylated form of histone 2AX [179].

##### Angiotensin II Receptor

Emerging data have demonstrated the importance of maintaining effective aortic vascular compliance during the metabolic aging process [181]. The therapeutic attenuation of both vascular stiffening and hypertension in the elderly represent a potentially effective pro-longevity intervention strategy [182]. As advanced aging is commensurate with increased degrees of DNA damage, it is therefore unsurprising that GPCR-associated factors that have strong hemodynamic functions, such as angiotensin II (Ang II), are also important regulators of the DDR process. Activation of the Ang II-associated renin-angiotensin-aldosterone system leads to the formation of ROS. Ang II-mediated stimulation of renal cell lines can induce DNA damage via activation of the Ang II type 1 receptor (AT1R) [183]. AT1R-mediared activation of NADPH oxidase (Nox4 subunit-containing isoform) causes the production of ROS, resulting in the formation of DNA strand breaks and micronuclei induction. In addition to DNA-damaging effects on renal cell systems, Ang II also has been shown to induce oxidative DNA damage and to accelerate the onset of cellular senescence in vascular smooth muscle cells (VSMCs). This pro-aging activity was ultimately shown to occur via telomere-dependent and independent mechanisms [184].

#### 3.3.2. β-Arrestin Family Proteins

Human β-arrestins comprise a small family of cytosolic proteins originally studied for their role in the desensitization and intracellular trafficking of GPCRs. Despite this humble beginning, the β-arrestins (β-arrestin1 (ARRB1) and β-arrestin2 (ARRB2)) have emerged as key regulators of multiple signaling pathways involved in aging. By acting as cellular scaffolding proteins that link vital signaling pathway entities to GPCRs, β-arrestins can exert homeostatic and ligand-responsive allostatic control of intermediary cell metabolic events and long-term cellular functionality [185]. As mentioned previously, Luttrell et al. [12] first demonstrated that β-arrestin interacts with Src family kinases and couples the receptor to MAPK ERK1/2 pathways that are associated with the regulation of both oxidative DNA damage [186] and DNA damage-associated cellular senescence [187]. β-arrestins have subsequently been demonstrated to bind a wide variety of kinases, E3 ubiquitin ligases, phosphodiesterases and transcription factors [110,185]. This ability of β-arrestins to connect GPCRs with these diverse signaling factors has greatly expanded the functional repertoire of these receptors. With respect to a direct association with β-arrestin-mediated signaling and DNA damage/repair pathways, early work indicated that stimulation of beta2-adrenergic receptors (β_2_ARs) promoted dephosphorylation of β-arrestin2 and its suppression of NF-kappaB (NF-κB) activation. NF-κB activation in response to UV-induced DNA damage is vital to maintain an effective DDR response [112]. Subsequent research into this intersection between β-arrestin-mediated signaling and DNA damage demonstrated that in both murine/human cell lines, β-arrestin1, after association with the active β_2_AR, induces an Akt-1-mediated activation of the E3 ubiquitin ligase, MDM2. This β-arrestin-dependent activation of MDM2 promotes the direct binding of this ligase to p53, thus promoting its degradation resulting in a detrimental effect upon the integrity of DDR systems, finally leading to increases in nuclear γ-H_2_AX adducts [188]. The apparent ability of circulating catecholamine stimulants of the β_2_AR, e.g., epinephrine and norepinephrine, to trigger GPCR-β-arrestin-mediated pro-DNA damage effects led to subsequent testing of these findings in murine models of stress, in which an elevated catecholamine drive would be present. Using an underwater trauma model of stress, Sood et al. [189] found that there was a steady-state increase in the physical association of the β_2_AR, β-arrestin1 and p53 with MDM2, thus creating a pro-DNA damage state in the CNS. Reinforcing this finding, Hara et al. [111] demonstrated that pharmacological blockade of this β-arrestin1-dependent p53-MDM2 signaling system was effective in reducing the extent of DNA damage induced by an applied behavioral stress to mice. As β-arrestin1 interacts with nearly all GPCR family proteins, this DNA damage cascade is unlikely to be specific to the β_2_AR system; for example, a simple pro-DNA damage β-arrestin1-p53-MDM2 signaling paradigm has been demonstrated for the previously mentioned MC1R [190]. These data therefore potentially suggest that if the activation of β2AR could be biased to signal exclusively through a non-β-arrestin mediated signaling paradigm, a reparative DDR response could be promoted. While these data evidently link the β-arrestin1 signaling pathway to the generation of DNA damage, in cases where induction of DNA damage may be desired (i.e., in oncology chemotherapy), the specific drug manipulation of β-arrestin1 activity may be beneficial to enhance chemosensitivity to co-administered anti-neoplastic agents [191].

#### 3.3.3. G Protein-Coupled Receptor Kinases and Associated Proteins

As we have discussed, stressful stimulation of GPCR systems, e.g., via circulating norepinephrine, can lead to pro-DNA damaging events. Therefore, the molecular mechanisms that control the sensitivity/activity of GPCRs may also be an important nexus for controlling age-related DNA damage. In response to ligand stimulation, the vast majority of GPCRs are reflexively ‘cut-off’ from generating further G protein-dependent signals via a ‘desensitization’ of the receptor. This tachyphylactic response is typically mediated by the phosphorylation of the receptor by heterologous desensitization (via second messenger-dependent protein kinases such as protein kinase A) and/or homologous desensitization (via a selective phosphorylation through a G protein-coupled receptor kinase (GRK)). Within seconds of receptor stimulation, these kinases phosphorylate serine and threonine residues within the intracellular domains of GPCRs, thereby uncoupling the receptors from heterotrimeric G proteins [192,193,194]. In addition to mediating this reflexive phosphorylation of activated GPCRs, GRKs also control phosphorylation independent cellular responses via their ability to interact with a broad spectrum of proteins involved in signaling and trafficking, e.g., PI3K (phosphoinositide 3-kinase), clathrin, caveolin, RKIP (Raf kinase inhibitor protein), MEK (mitogen-activated protein kinase kinase), Akt (protein kinase B) and GIT (GRK-interacting transcript) proteins [195,196,197,198]. This scaffolding function of GRKs allows them to act as potential structural regulators that may control the organization of GPCR-based receptorsomes.

GRKs belong to a coherent family of associated proteins that all share at least a similar kinase activity. The GRK superfamily of related proteins can be subdivided into three main groups based on sequence homology: (i) rhodopsin kinase or visual GRK subfamily (GRK1 and GRK7); (ii) the β-adrenergic receptor kinases subfamily (GRK2/GRK3); (iii) the GRK4 subfamily (GRK4, GRK5 and GRK6). These kinases share certain characteristics, but are distinct enzymes with specific regulatory properties. GRK2, 3, 5 and 6 are ubiquitously expressed in mammalian tissues, whereas GRK1/7 (retina) and 4 (cerebellum, kidney, gonads) demonstrate more tissue-specific expression patterns [199,200,201]. With respect to a potential role of GRKs in the DDR realm, it was first noted that genomic reduction of GRK5 expression in osteosarcoma cells inhibited DNA damage-induced apoptosis via a p53-mediated mechanism [202]. It was subsequently demonstrated that p53 was a high-affinity substrate of GRK5 and its phosphorylation by this kinase led to its degradation and subsequent inhibition of the p53-dependent apoptotic response to genotoxic damage. This association of GRK5 with the DDR pathway was shown to be highly selective, as neither GRK2 nor GRK6 could mediate this p53 phosphorylation. Demonstrating the importance of this pathway, it has been shown that GRK5-deficient mice possess an elevated p53 expression level, leading to an elevated irradiation-induced apoptotic sensitivity. Commensurate with this functional role in DDR processes and cell damage, it has been demonstrated that GRK5 deficiency predisposes model organisms to age-related neurodegeneration, cognitive dysfunction and loss of synaptic plasticity [203,204,205]. In the cardiovascular setting, however, age-related pathologies have also been associated with elevated GRK5 expression [206].

As previously mentioned, many GRK-interacting proteins mediate other significant signaling functions. One of these proteins that possesses an important role in controlling DDR is the GRK-interacting transcript 2 (GIT2). GIT2 is a widely-expressed ADP-ribosylation factor GTPase-activating protein (Arf-GAP) [8,207,208,209]. GIT2 was identified as an important protein linked to several aspects of the complex neurometabolic aging process through latent semantic indexing (LSI)-based interrogation of high-dimensionality hypothalamic proteomic datasets gathered from longitudinal analysis of aging rats [8]. It was further demonstrated that an age-dependent elevation of the expression of GIT2 in the hypothalamus (as well as other brain regions) was found in non-human primates, as well as humans [8]. These findings were also supported by the demonstration of elevated expression levels of GIT2 in human neuronal cells exposed to increasing oxidative stress levels [37]. Additional investigations revealed that GIT2 interacts with many proteins involved in multiple signaling pathways linked to aging such as ATM, p53 and BRCA1. All of these proteins are involved in stress-responsive cascades and play important roles in cell cycle/DDR control, circadian clock regulation [157,210,211] and generation of SASP phenotypes in immune tissues [211]. Ectopic elevation of GIT2 expression in neuronal and non-neuronal tissues is able to attenuate the extent of DNA DSB damage induced by both ionizing radiation and chemotherapeutic DNA-damaging agents (cisplatin) [210]. Further reinforcing this permissive role of GIT2 in the aging process, it was shown that genomic deletion of GIT2 resulted in an accelerated rate of γ-H_2_AX lesion inclusion in central nervous cortex tissue in experimental mice [210]. In addition to the damage caused to brain tissues in GIT2 knockout (GIT2KO) mice, it was recently demonstrated that genomic deletion of GIT2 led to a significant co-reduction of multiple circadian clock-related mRNA transcripts in a broad range of immunological tissues including spleen, thymus and multiple lymph nodes [211]. This downregulation of GIT2 with associated clock-related proteins has been associated with premature aging (evidenced by accelerated thymic involution), the creation of a SASP-like phenotype and DDR functions [211]. These data together suggest that GIT2 may act as a functional connector between cellular senescence, clock regulation and DNA damage repair and as such could possess the capacity to alter the accumulation of age-related cellular damage. Therefore, GIT2 might represent a crucial therapeutic target to attenuate age-related metabolic decline. Classical therapeutic targets however are usually receptors, ion channels, kinases and phosphatases; hence, as GIT2 is a scaffolding protein, it does not represent a typical therapeutic target [212]. The demonstration that, in addition to regulating intermediary cell metabolism events, GPCRs can also effectively regulate the expression profiles of multiple signaling proteins via non-G protein-dependent functions [13,14,213] facilitates an important expanded capacity for drug development. Hence, it is likely that in the future, GPCRs can be employed to control the expression profile of specific non-canonical signaling proteins, e.g., GIT2 [13,107]. In this scenario, the GPCR target would be chosen for its capacity to control the expression of network-controlling regulators (e.g., GIT2) and their associated factors rather than just modulating a single protein target. Therefore, in order to target and control GIT2, it is imperative to find a GPCR that can modulate the function and expression of this scaffolding protein. To identify a GPCR strongly associated with GIT2, GIT2KO mice were used recently to investigate expression relationships across multiple tissues [212]. In GIT2KO mice, a consistently downregulated GPCR, the Relaxin 3 family peptide receptor (RXFP3), was found in the murine CNS, pancreas and liver [212]. The therapeutic control of this GPCR therefore may represent a facile system with which to control the expression profile of GIT2 in tissues and therefore regulate aging-related cellular damage in a trophic manner.

#### 3.3.4. Regulator of G Protein Signaling Proteins

The regulation of GPCR activity is highly complex and well controlled, with multiple layers of interconnected signaling pathways activated upon receptor stimulation that feedback to modulate receptor signaling. The most studied GPCR signal ‘conditioning’ mechanisms are mediated by GRKs and β-arrestins; however, an extra level of control is common to many GPCRs, as well, i.e., that exerted by the regulator of G protein-signaling (RGS) proteins [214]. RGS proteins control the activity of GPCRs via their ability to control heterotrimeric G protein signaling negatively by accelerating the Gα subunit GTP hydrolytic activity, thus helping to determine the magnitude and duration of the cellular response to GPCR stimulation [215]. It is interesting to note, however, given our current knowledge of non-G protein-dependent GPCR signaling, that indeed RGS proteins may conversely represent themselves as positive stimulators of these recently identified pathways.

Presently, there are thought to be at least twenty canonical RGS protein versions found in mammals [214]. These members of the RGS superfamily are divided into four subfamilies based on sequence homology and the presence and nature of additional non-RGS domains. With respect to the involvement of RGS proteins in the dynamics of DDR responses, early research indicated that disruptions to RGS protein (RGS16, RGSL1/RGSL2 (RGS-like proteins 1/2)) expression/functions were mediated in human breast carcinomas through DNA fragility within the HPC1 region in chromosome 1 [216]. As with many GPCR-system interactions with the DDR process, the cell cycle regulator p53 clearly exerts a trophic functional role, e.g., within immune cells, the cellular expression profile of RGS13 was demonstrated to be suppressed by prevailing p53 activity [217]. Reinforcing the importance of p53-mediated signaling associated with RGS protein functionality, Huang et al. [218] demonstrated that the anti-neoplastic agent doxorubicin activates ATM and p53 through an RGS6- and ROS-dependent signaling process. Interestingly, this ROS/RGS6-dependent ATM-activating mechanism was found to be functionally independent of actual physical DNA damage [218]. This RGS6-dependent ATM/p53 mechanism has also been shown to be relevant in myocardial apoptosis; this finding therefore introduces the potential to reduce the harmful cardiotoxic effects of human doxorubicin oncological treatment regimens in the future [219]. Further research investigating the role of RGS6 in ATM activation found that mammary epithelial cells (MECs), isolated from RGS6-null mice, demonstrated a deficit in ATM/p53 activation, ROS generation and apoptosis in response to the DNA damaging agent DMBA (7,12-dimethylbenza[α]anthracene), confirming that RGS6 was required for effective activation of the DDR in these cells [220]. These data suggested that RGS6 might be a potent natural inhibitor of breast cancer initiation and progress, thereby presenting a new capacity for future breast cancer treatment. An unexpected intersection between the RGS system and DDR responses was recently found by Sjögren et al. [221] during an unbiased genomic siRNA screening approach to uncover mechanisms that control proteasomal degradation pathways for RGS2. This research team was able to identify a novel E3 ligase complex containing cullin 4B (CUL4B), DNA damage binding protein 1 (DDB1) and F-box protein 44 (FBXO44) that mediates RGS2 protein degradation. DDB1 is a multifunctional DDR-associated factor initially isolated as a subunit of a heterodimeric complex that recognizes ultraviolet radiation-induced DNA lesions in the NER pathway [222]. Therefore, within this screen, it was clear that a functional link with the DDR system was evidenced by the presence of DDB1 in the RGS2-controlling interactome.

#### 3.3.5. Non-Canonical GPCR-Interacting Proteins

It is evident from the growing body of literature concerning the functional and effective intersections between the GPCR and DDR systems that future research into this paradigm will hopefully yield actionable therapeutic strategies to mitigate age-associated DNA damage and the age-related disorders this damage triggers. So far, we have shown that GPCRs themselves, β-arrestins, GRKs and their interacting proteins, as well as RGS proteins can play important regulatory roles in DNA-management processes. As we have stated before, however, the true functional spectrum of GPCR-system associated proteins, including ones likely to affect the stoichiometry of GPCR receptorsome structures, has yet to be conclusively mapped. Therefore, in this final section, we shall discuss the role(s) of other non-canonical GPCR-interacting factors that also control the functional intersection of GPCR and DDR signaling systems.

##### Regulated in Development and DNA Damage Responses 

The availability of cellular nutrients and prevalent metabolic energy levels are functionally detected by signaling mechanisms that involve the mTORC1 (mammalian target of rapamycin complex 1) kinase. In response to the presence or absence of these stimuli, mTORC1 can control cell growth and viability. The cellular ability to maintain energy homeostasis is tightly linked to a cells’ capacity to maintain DNA integrity and stability. To this end, the catalytic activity of mTORC1 can be inhibited by the absence of sufficient nutrients or via the sensation of cellular stressors through the responsive overexpression of REDD1 (regulated in development and DNA damage responses) [223]. REDD1 was initially identified as a crucial developmentally-regulated factor that connects p53 signaling to the cellular regulation of ROS-sensitivity, thus suggesting its role in DDR activities [224]. Researchers have recently shown that this mTORC1-regulatory protein demonstrates a strong functional link to GPCR-systems. Michel and co-workers [225] employed a quantitative BRET (bioluminescent resonance energy transfer)-based plasma membrane localization assay to screen for the ability of a panel of endogenously-expressed calcium-mobilizing GPCRs to induce plasma membrane translocation of REDD1. This research team demonstrated that REDD1 and its mTORC1-inhibitory motif participate in the GPCR-evoked dynamic interaction of REDD1 with the plasma membrane, thus identifying this novel DDR-associated protein as a new effector in GPCR signaling. Translocation to the plasma membrane appears to be an inactivation mechanism of REDD1 by GPCRs. This GPCR-mediated inactivation process is most likely via the resultant sequestration of REDD1’s functional mTORC1-inhibitory motif.

##### Fanconi Anemia A Protein

Fanconi anemia (FA) is a rare genetic disease resulting in impaired responses to DNA damage. Among FA patients, the majority develop cancer, most often acute myelogenous leukemia, and 90% develop bone marrow failure (the inability to produce blood cells) by the age of 40. Over two-thirds of FA patients present with congenital defects including: short stature; abnormalities of the skin, arms, head, eyes, kidneys, ears, developmental disabilities and infertility [226]. FA is the result of a genetic defect in a cluster of proteins responsible for DNA damage repair via homologous recombination and is considered to be a classical ‘genome instability’ disorder. FA is therefore formally defined as an acquired state that allows for an increased rate of spontaneous genetic mutations throughout each replicative cell cycle [227,228]. To date, 17 different Fanconi anemia proteins (FANC A, B, C, D1, D2, E, F, G, I, J, L, M, N, P, S and RAD51C, XPF) are currently known to exist; disruption of these can lead to the genomic instability characteristic of FA [228]. The FANCA protein is found to be responsible for approximately 64% of FA cases [229,230], suggesting that this specific FA protein holds a singular position in the maintenance of genome integrity. In addition to its role in genomic stability, FANCA has been shown to also be a key regulator of GPCR activity. Larder and co-workers [231] demonstrated that the expression levels of FANCA, in pituitary gonadotrope cell lines, were controlled by gonadotropin-releasing hormone (GnRH)-mediated stimulation of its cognate GPCR. Upon GnRH-induced expression of FANCA, it was shown to adopt an intracellular nucleocytoplasmic distribution pattern constitutively. Protracted GnRH receptor stimulation was shown to induce a nuclear accumulation of FANCA before eventually trafficking back to the cytoplasm via the nuclear export receptor CRM1 (chromosome region maintenance 1 protein homolog). FANCA was subsequently demonstrated to be vital in allowing GnRH to control the expression of the gonadotropin hormones, i.e., luteinizing and follicle-stimulating hormones. Regulating the transcriptional control of these two hormones offers a convincing explanation of the infertility issues found in FA patients. It was concluded from this study that FANCA could be considered as a novel signal transducer of the GnRH receptor.

##### Poly(ADP-ribose) Polymerase 1 Protein

The poly(ADP-ribose) polymerase (PARP1) protein is directly involved in the BER DDR pathway. PARP1 catalyzes the poly(ADP-ribosyl)ation of a number of acceptor proteins involved in the regulation of chromatin architecture, as well as DNA metabolism. This poly(ADP-ribosyl)ation tracks DNA damages and represents a crucial step in the sensory signaling pathway leading to the repair of DNA strand breaks [232,233,234,235]. Demonstrating the tight functional links between DDR and the aging process, it has been shown that the prevailing PARP1 activity, measured in the permeabilized mononuclear leukocytes of thirteen mammalian species (rat, guinea pig, rabbit, marmoset, sheep, pig, cattle, pygmy chimpanzee, horse, donkey, gorilla elephant and man), predictably correlates with the maximum lifespan of these species [236]. In recent years, the scope of functionality of DDR proteins, e.g., BRCA1, has expanded to include effective roles in age-related disorders of cognition such as AD [237]. Recent studies have also indicated that PARP1 may be a new nuclear target in AD-related signal transduction pathways [238]. Further studies into the PARP1 connection with AD have found that muscarinic acetylcholine (mAChR) GPCR stimulation can fully activate hippocampal PARP1 through a calcium mobilization-dependent and ROS independent process [239]. This cholinergic GPCR-dependent PARP1 activation was abolished by the administration of a pro-AD amyloidogenic peptide (Amyloid beta 25–35) to experimental mice. This toxic pathological peptide itself significantly stimulated PARP1 activity by inducing ROS-mediated DNA damage. These data suggest that toxic amyloid beta peptides can affect mAChR-dependent signal transduction to PARP1, probably via ROS interdiction and inhibition of ligand-induced calcium mobilization. PARP1 therefore effectively serves as a downstream effector of the mAChRs that form the prime functional target of current AD therapeutics such as the cholinesterase inhibitor Aricept^®^.

Further to the role of PARP1 in receptor-mediated protection of CNS DNA, it has been shown in human neuronal cells that the aging keystone GIT2 forms active complexes with both PARP1 and PARP2 in response to DNA-damaging stress caused by cisplatin treatment or ionizing radiation. The interaction of GIT2 served to enhance the signaling activity of PARP1 in these cells and likely contributed to the DNA-protecting activity of the GIT2 protein [210].

##### Angiotensin II Type 2 Receptor-Interacting Protein 

We have previously indicated that with respect to age-related cardiovascular pathophysiologies, the Ang II ligand-receptor system is an important player in this paradigm (Section 3.3.1). However, in addition to the role of angiotensin receptors in DDR processes, recent research has demonstrated that additional GPCR interacting proteins can also condition the output of this receptor system. Ang II can functionally interact in a selective manner with two major cell surface GPCRs, angiotensin type 1 receptor (AT1R) and angiotensin type 2 receptor (AT2R). As discussed previously, Ang II is closely associated with vascular diseases and vascular remodeling. Since vascular senescence plays a critical role in vascular aging and age-related vascular diseases, this pro-aging process can be functionally enhanced by AT1R stimulation [240,241]. Conversely, AT2R stimulation generates the opposite signaling output and can functionally antagonize AT1R-mediated vascular senescence [242]. It has been demonstrated that multiple non-G protein interacting partners synergize with the Ang II GPCRs to regulate this complex interplay with respect to cellular senescence control. For example, the AT1R–interacting protein (ATRAP) attenuates the ability of the AT1R to induce vascular senescence [243,244]. A direct binding partner of the AT2R, i.e., the AT2R-interacting protein (ATIP) [245], has been shown to control vascular senescence behavior, as well [246]. ATIP interaction with the AT2R appears to play an important role in AT2R control of the senescent process. Hence, Min and co-workers [246] investigated the functional mechanisms of this system in a transgenic murine system. Transgenic mice were created overexpressing the ATIP protein and were employed to derive primary VSMC cultures. Chronic Ang II stimulation of VSMCs from wild-type mice resulted in the increase of the DNA damage marker, 8-OHdG. This damaging effect of Ang II was significantly attenuated in the VSMCs of ATIP transgenic mice after similar treatment with Ang II. VSMCs of ATIP transgenic mice, in response to chronic Ang II stimulation, showed a greater elevation of the DNA repair factor methyl methanesulfonate-sensitive 2 (MMS2) levels compared to wild-type controls. Significantly less aortic 8-OHdG expression was found, along with a more potent elevation in MMS2 levels in the ATIP transgenic mice compared to controls following whole-body irradiation of wild-type and ATIP transgenic mice. Thus, the ATIP GPCR interacting protein was shown to possess the capacity to attenuate the extent of DNA damage while augmenting the degree of damage repair.

## 4. The GPCR-DDR Signaling Intersection and Its Potential Therapeutic Exploitation

**Figure 1:**
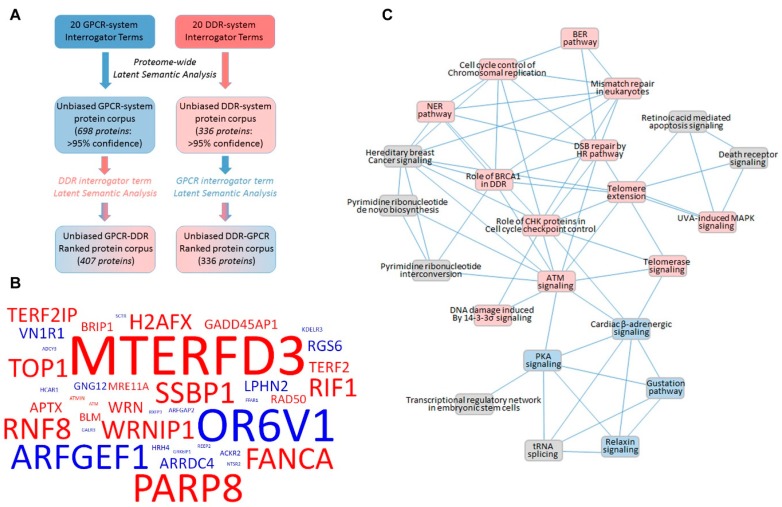
Unbiased informatics appraisal of functional intersection between GPCR and DNA damage-response (DDR) systems. (**A**) Protein identities, semantically associated with GPCR-related (blue) or DDR-related (red), were generated from whole proteome-wide datasets created from PubMed abstracts. The most strongly GPCR or DDR system-associated protein lists were then cross-interrogated using the opposing interrogator term list. (**B**) Wordcloud representation (using cosine similarity score values) of both GPCR- (blue text) and DDR-intersectional protein factors. The font size of the protein term is proportional to the cumulative cosine similarity score values across the multiple (17) interrogator terms. (**C**) Canonical signaling pathway analysis was applied to the combined GPCR or DDR-associated protein lists (Panel A) created using cross-interrogation. Displaying the pathways linked by common signaling proteins reveals the connections between DDR-associated (red) and GPCR-associated (blue) cellular signaling cascades.

In recent years, ever stronger connections have been observed between the functional realms of GPCR and DDR systems. Both signaling systems comprise a highly important and organized set of interacting proteins that together connect and coordinate physiological responses to multiple stressors experienced during an organism’s lifespan. To illustrate this important functional intersection in an unbiased manner, we employed latent semantic analysis [247,248] of biomedical text corpora (extracted from all available public texts at PubMed Central) using interrogator text terms associated with GPCR or DDR signaling systems (Table S1). This biomedical text interrogation yields protein lists with a measurable extent of scientific text association (cosine similarity score: ranging from 0.1 for the lowest to 1.0 for the strongest associations: [249]) between the interrogator terms and the identified proteins. To refine these GPCR- or DDR-associated proteins, we extracted the 95% percentile most strongly associated proteins (Table S2, GPCR; Table S3, DDR). To investigate the functional crossovers between these unbiased protein lists, we cross-interrogated the GPCR-associated protein list (Table S2) with the DDR interrogator terms (Table S1); this generated the protein list detailed in Table S4. In addition, we also cross-interrogated the DDR-associated protein list (Table S3) with the GPCR interrogator terms (Table S1), generating the protein list detailed in Table S5. This protocol therefore generated a list of proteins, identified using unbiased informatic text analysis, linking both GPCR and DDR systems (Figure 1A). For illustrative purposes, we created a wordcloud using 20 proteins from the DDR term interrogation of the GPCR list (Table S4, blue text) and 20 proteins from the GPCR term interrogation of the DDR list (Table S5, red text). In the resulting wordcloud (Figure 1B), the protein term size is proportional to the cumulative cosine similarity score (indicative of strength of GPCR/DDR association) for this protein. Within this cloud, many of the factors discussed in this review are evident (e.g., RXFP3, HCAR1, PARP, FANCA, etc.); details of the protein descriptions of these GPCR-DDR intersection factors are outlined in Table S6 (GPCR list, blue) and S7 (DDR list, red). In addition to this unbiased interaction between GPCR and DDR systems, we also employed canonical signaling pathway analysis (ingenuity pathway analysis (IPA)) of the combined protein lists from Tables S4 and S5. From this pathway-based annotation, a list of significantly regulated signaling cascades was generated (Table S8). Plotting the intersection (Figure 1C) between pathways that share the same proteins (>2 common factors) revealed that multiple connections were present between DDR-associated signaling (red) and GPCR-associated signaling (blue) systems. Therefore, using entirely unbiased semantic analysis of publicly-available biomedical texts, the viability of our posit that these two crucial signaling systems are functionally interconnected has been shown.

With a more advanced understanding of the therapeutically-tractable points of intersection between these two systems, it might be possible to create a novel series of drug-based strategies rationally to regulate genomic stability and the aging process. These multifunctional GPCR-DDR controllers may likely demonstrate the capacity to retard the onset of major debilitating age-associated diseases. It has been demonstrated over several decades that perhaps the most effective mechanism of drug development lies in the exploitation of GPCR signaling systems. Research into the nuances of GPCR signaling have revealed the potential for new avenues of therapeutic discovery based on the selective regulation of GPCR signaling. Historically, some of the earliest theories of GPCR signaling considered that the receptor exists in a simple two-state equilibrium between “on” or “off” states distinguished by their ability to trigger downstream responses [250]. Further advancement of this concept, via the creation of constitutively active mutant receptors, led to the widely accepted ternary and extended ternary complex models of GPCR activation [251,252]. Within these models, the mechanisms by which complex ligand signaling behaves could be better appreciated, i.e., stimulating GPCR ligands could alter this equilibrium in different ways and hence were classified as agonists, partial agonists, inverse agonists and antagonists [103,253]. Agonists provoke a maximal response of the GPCR, whereas partial agonists generate a submaximal response at saturating ligand concentrations. Classical antagonists were considered to simply lack all receptor efficacy, yet among these agents, many were found to possess inverse agonist activity, i.e., the ability to attenuate basal G protein activation status. Such simple ligand/drug classification has largely been relegated to historical interest since the advent of the demonstration of multiple signaling system coupling, ligand bias/agonist trafficking and non-G protein-dependent GPCR signaling [12,103,107,253]. Therefore, it is clear that with respect to GPCR signaling classification, there is likely to be a broad spectrum of multiple ‘on’ states at all times. It is our proposal that after protein translation and membrane insertion, that a single GPCR is never ‘off’ or inactive. In this paradigm, depending on the nature and type of GPCR receptorsomes present in the cell, ligands/drugs will possess an ability to stabilize/de-stabilize a percentage of these multiple ‘on’ states to mediate their cellular activity [106]. In addition to possessing this ‘spectrum’ functionality of signaling, the subcellular localization aspect of GPCR signaling has a profound impact upon future drug design, especially with respect to the molecular intersection between GPCR and DDR realms. Much of our knowledge of GPCR signaling is concerned with the analysis of ligand-dependent signals that emanate from stimulated cell surface receptors [254]; here, the activated receptors (stimulated via ligand stimulation or constitutive basal activation) can elicit a broad range of cellular responses depending on receptorsome formation and eventual subcellular trafficking or desensitization. This plasma membrane-focused signaling paradigm we can describe as ‘*Model 1*′ signaling. However, considerable emerging data suggest that actively signaling GPCRs are not solely associated with the plasma membrane. Instead, GPCR signaling can also emanate from various intracellular membrane structures and can display distinct signaling features such as diverse receptorsome structures, altered lipid environments or differential ‘stimulator’ sensitivities [113,121,255,256,257]. While the classical perspective that GPCRs can be activated at the plasma membrane and subsequently be transported to the intracellular membranes (*Model 1*) still holds true, it is now evident that GPCRs can be activated at intracellular membranes through intracellularly-synthesized stimulators, as well as membrane-permeable or even endocytosed receptor ligands [256]. To allow this intracellular signaling, it might be necessary that GPCRs are atypically inserted in the intracellular membranes [255] to allow cytoplasmic ligand/stimulator interactions [255]. In this case, GPCRs may be able to function as intracellular stress sensors, e.g., for ROS or lactate, and signal from inside the cell to the outside or to other cellular compartments; this differential signaling behavior we have codified as ‘*Model 2*′ signaling. It is interesting to note that the majority of our receptor activation theories (as well as drug design strategies) have been entirely based upon the *Model 1* concept. In addition, our molecular and structural appreciation of GPCR activation has also been driven from a *Model 1*-biased standpoint. One widely investigated aspect of *Model 1* signaling is the well-characterized rhodopsin-like receptor transmembrane helix 3 Asp-Arg-Tyr (DRY) motif [258] that is thought to control agonist-induced conformational changes in the receptor. Naturally occurring receptor mutations in the DRY-motif are considered to disrupt normal G protein-dependent signaling in rhodopsin-like GPCRs and increase the amount of intracellularly-retained receptor [259]. Does this specific combination of events then pre-dispose such mutated receptor forms to adopt a propensity for *Model 2* signaling? If so, then perhaps a re-adjustment of our concepts of receptor activation and ligand sensitivity will be important to potentially exploit the presence of these intracellular *Model 2* receptors. A more thorough study of such *Model 2* receptors may represent an important resource to identify potential GPCR sensors of damage that can then synergize productively with the DDR machinery to reduce age/metabolism-dependent DNA damage.

## 5. Conclusions

Taken together, our aggregated findings suggest that multiple components of the GPCR signaling system can modulate the activity of signaling proteins directly or indirectly involved in DNA damage and/or repair. As such, GPCR signaling systems may represent multifunctional sensors for DNA damaging insults, and their rational exploitation via novel drug design may facilitate our ability to augment DNA repair processes therapeutically. Thus, GPCR systems may have long evolved side-by-side with emerging DDR systems to act as sensors, and ameliorative effectors, for intracellular DNA damage and age-related stresses.

## Supplementary Materials

Supplementary materials can be found at http://www.mdpi.com/1422-0067/19/10/2919/s1.
