# Supplementary material for: G Protein-Coupled Receptor Systems as Crucial Regulators of DNA Damage Response Processes
Source: Int J Mol Sci. 2018 Sep 26;19(10):2919. doi: 10.3390/ijms19102919 (PMC6213947; doi:10.3390/ijms19102919)
Supplement: Supplementary file 1 [file ijms-19-02919-s001.pdf]

G Protein-Coupled Receptor Systems as Crucial Regulators of DNA Damage Response Processes

Supplementary Data

Table S1. Latent semantic indexing GPCR and DDR concept terms.

Table S2. GeneIndexer latent semantic data extraction of GPCR system associated proteins.

Table S3. GeneIndexer latent semantic data extraction of DDR system associated proteins.

Table S4. DDR concept term interrogation of the LSI-based GPCR system corpus.

Table S5. GPCR concept term interrogation of the LSI-based DDR system corpus.

Table S6. GPCR-associated factors from the LSI-generated GPCR-DDR interaction cloud.

Table S7. DDR-associated factors from the LSI-generated GPCR-DDR interaction cloud.

Table S8. Ingenuity signaling pathway analysis of the combined GPCR and DDR system LSI-based lists.

Table S1. Latent semantic indexing GPCR and DDR concept terms. GeneIndexer interrogator terms employed to generate semantically-associated protein lists associated with GPCR (G protein-coupled receptor) or DDR (DNA damage response/repair) systems.

GPCR System
GPCR
G protein-coupled receptor
Heptahelical receptor
Heptahelical

Serpentine receptor
7 transmembrane receptor
G protein
Arrestin
Beta arrestin
Beta-arrestin
RGS protein
Regulator of G protein signaling
GRK
G protein-coupled receptor kinase
GIT2
G protein-coupled receptor kinase interacting transcript 2

DDR System
DNA damage response
DNA damage
Double-strand break
DNA damage repair
Single-strand break
Base excision repair
Nucleotide excision repair
Homologous recombination
Non-homologous end-joining
DSB
SSB
Genotoxic stress
Genotoxic
DDR
Senescence
Cell cycle checkpoint
Cell cycle checkpoint

Table S2. GeneIndexer latent semantic data extraction of GPCR system-associated proteins. For each specific protein, identified by its official gene symbol, the cosine similarity score (at least >0.1, therefore indicating an implicit association) for the strength of latent semantic association with the GPCR-specific concept terms (from Table S1) is given. The sum of the specific concept occurrences, the average and median cosine similarity score and the average rank within the matrix are given for each specific protein.

Gene symbol	GPCR	G protein-coupled receptor	Heptahelical receptor	Heptahelical	Serpentine receptor	7 transmembrane receptor	G protein	Arrestin	Beta-arrestin	Beta-arrestin	RGS protein	Regulator of G protein signaling	GRK	G protein-coupled receptor kinase	GIT2	G protein-coupled receptor kinase interacting transcript 2	Sum of occurrences	Average score	Median score	Average rank
ARF6	0.404	0.39	0.372	0.372	0.118	0.162	0.419	0.35	0.354	0.378	0.175	0.223	0.362	0.395	0.442	0.547	16	0.3414375	0.372	382.625
GPR79	0.343	0.395	0.345	0.345	0.231	0.117	0.264	0.194	0.199	0.144	0.284	0.131	0.283	0.396	0.1	0.513	16	0.26775	0.2735	1442.8125
RXFP4	0.338	0.407	0.331	0.331	0.157	0.138	0.244	0.124	0.128	0.125	0.229	0.121	0.176	0.409	0.118	0.514	16	0.243125	0.2025	1522.1875
TRHR	0.555	0.551	0.529	0.528	0.475	0.145	0.214	0.415	0.422	0.446	0.236	0.107	0.479	0.552	0	0.561	15	0.414333333	0.475	1656.266667
GPR3	0.501	0.516	0.467	0.467	0.168	0.233	0.305	0.385	0.388	0.381	0.249	0.265	0.469	0.52	0	0.585	15	0.393266667	0.388	483.8666667
RGS7BP	0.493	0.585	0.436	0.436	0	0.105	0.315	0.199	0.204	0.152	0.778	0.177	0.484	0.587	0.152	0.624	15	0.3818	0.436	1059.866667
RXFP1	0.432	0.461	0.47	0.47	0.242	0.151	0.389	0.319	0.326	0.344	0.159	0.216	0.32	0.463	0	0.623	15	0.359	0.344	481.8666667
CYTH2	0.428	0.433	0.417	0.417	0	0.152	0.486	0.347	0.352	0.369	0.207	0.235	0.374	0.44	0.291	0.618	15	0.371066667	0.374	296.2666667
GPR142	0.407	0.521	0.43	0.43	0.223	0.118	0.211	0.159	0.162	0.152	0.472	0.128	0.297	0.522	0	0.541	15	0.3182	0.297	1671.8
P2RY14	0.384	0.467	0.343	0.342	0.138	0.238	0.249	0.15	0.154	0.136	0.446	0.232	0.307	0.468	0	0.502	15	0.303733333	0.307	825.8
GNB2L1	0.362	0.345	0.315	0.315	0	0.12	0.353	0.336	0.342	0.371	0.14	0.206	0.423	0.354	0.108	0.434	15	0.3016	0.342	820.3333333
LPHN2	0.332	0.374	0.308	0.308	0	0.234	0.484	0.195	0.201	0.16	0.309	0.271	0.26	0.379	0.128	0.656	15	0.3066	0.308	224.4666667
OXER1	0.295	0.335	0.251	0.251	0.114	0.186	0.311	0.125	0.129	0.13	0.23	0.256	0.19	0.338	0	0.453	15	0.2396	0.251	651.4
GPR27	0.29	0.34	0.266	0.265	0.144	0.168	0.479	0.15	0.156	0.134	0.198	0.266	0.203	0.345	0	0.64	15	0.2696	0.265	294.9333333
PDCL	0.282	0.351	0.269	0.269	0	0.116	0.482	0.11	0.115	0.11	0.384	0.223	0.31	0.358	0.104	0.544	15	0.268466667	0.269	615.3333333
GPR158	0.26	0.3	0.252	0.252	0	0.11	0.322	0.172	0.175	0.122	0.297	0.2	0.247	0.302	0.1	0.501	15	0.2408	0.252	1017.866667
GPR61	0.258	0.291	0.183	0.183	0.131	0.115	0.249	0.11	0.113	0.116	0.168	0.138	0.159	0.293	0	0.41	15	0.194466667	0.168	1673.533333
CYTH4	0.252	0.283	0.22	0.22	0	0.135	0.423	0.133	0.139	0.127	0.199	0.228	0.171	0.291	0.327	0.497	15	0.243	0.22	548.4666667
IPCEF1	0.25	0.244	0.2	0.2	0	0.112	0.481	0.183	0.188	0.188	0.123	0.255	0.197	0.253	0.306	0.502	15	0.245466667	0.2	544.4
AKAP6	0.249	0.295	0.191	0.191	0	0.159	0.496	0.127	0.134	0.142	0.213	0.251	0.274	0.305	0.107	0.507	15	0.242733333	0.213	411.8666667
GDPD2	0.246	0.288	0.262	0.262	0.639	0.181	0.329	0.159	0.163	0	0.156	0.199	0.143	0.292	0.156	0.454	15	0.261933333	0.246	779.6

ARF1	0.224	0.25	0.196	0.196	0	0.162	0.512	0.102	0.106	0.105	0.188	0.254	0.161	0.257	0.334	0.527	15	0.238266667	0.196	386.6666667
ARF5	0.22	0.24	0.213	0.213	0	0.125	0.423	0.156	0.16	0.161	0.166	0.21	0.199	0.246	0.574	0.47	15	0.251733333	0.213	649.1333333
RPLP2	0.217	0.233	0.184	0.184	0.115	0.189	0.372	0.117	0.122	0.124	0.144	0.251	0.192	0.236	0	0.418	15	0.206533333	0.189	587.8
ARF3	0.192	0.212	0.208	0.208	0	0.15	0.524	0.127	0.132	0.131	0.164	0.243	0.161	0.218	0.193	0.561	15	0.228266667	0.193	439
TMEM217	0.191	0.215	0.15	0.15	0	0.119	0.53	0.15	0.153	0.149	0.113	0.26	0.213	0.22	0.102	0.567	15	0.2188	0.153	595.4666667
IQSEC1	0.189	0.166	0.147	0.147	0	0.119	0.285	0.124	0.125	0.14	0.101	0.111	0.136	0.172	0.179	0.332	15	0.164866667	0.147	1947.133333
BBIP1	0.171	0.181	0.132	0.132	0	0.191	0.492	0.118	0.122	0.1	0.106	0.286	0.116	0.186	0.135	0.516	15	0.198933333	0.135	463.9333333
GRK5	0.828	0.821	0.631	0.631	0	0	0.163	0.648	0.658	0.65	0.429	0.101	0.874	0.822	0.199	0.627	14	0.577285714	0.6395	1896.071429
GRK6	0.82	0.853	0.653	0.653	0	0	0.188	0.589	0.593	0.604	0.494	0.115	0.812	0.854	0.171	0.681	14	0.577142857	0.6285	1693.214286
ADRBK1	0.783	0.843	0.685	0.685	0	0	0.141	0.492	0.497	0.52	0.703	0.102	0.876	0.844	0.251	0.624	14	0.574714286	0.6545	1964.642857
MCIDAS	0.672	0.566	0.602	0.602	0	0	0.225	0.774	0.775	0.814	0.147	0.165	0.681	0.567	0.13	0.553	14	0.5195	0.5845	1237.928571
AP1B1	0.584	0.478	0.521	0.521	0	0.118	0.324	0.673	0.675	0.714	0	0.155	0.547	0.482	0.16	0.55	14	0.464428571	0.521	1121.642857
GNB1	0.512	0.637	0.485	0.485	0	0	0.224	0.17	0.173	0.131	0.875	0.129	0.532	0.64	0.223	0.58	14	0.414	0.485	1474.714286
GNG2	0.512	0.632	0.482	0.482	0	0	0.179	0.141	0.144	0.133	0.882	0.118	0.531	0.633	0.208	0.538	14	0.401071429	0.482	1760.428571
PTAFR	0.508	0.491	0.483	0.483	0	0	0.271	0.443	0.446	0.477	0.211	0.172	0.445	0.496	0.119	0.46	14	0.393214286	0.453	1024.857143
PDE4D	0.495	0.464	0.433	0.433	0.134	0	0.308	0.494	0.499	0.521	0.143	0.13	0.509	0.468	0	0.463	14	0.392428571	0.4635	1154.071429
RGS8	0.495	0.618	0.47	0.47	0	0	0.173	0.135	0.138	0.127	0.882	0.119	0.502	0.618	0.155	0.527	14	0.387785714	0.47	1802.428571
OR1D2	0.463	0.444	0.442	0.442	0	0.251	0.427	0.401	0.407	0.413	0.252	0.243	0.427	0.445	0	0.598	14	0.403928571	0.427	238.5
PPP1R9B	0.457	0.438	0.412	0.412	0	0	0.247	0.426	0.428	0.416	0.166	0.119	0.445	0.442	0.155	0.461	14	0.358857143	0.421	1484.285714
NTSR1	0.456	0.46	0.427	0.426	0.107	0.116	0.268	0.385	0.389	0.41	0	0.11	0.349	0.462	0	0.525	14	0.349285714	0.3995	1677.571429
CLTC	0.452	0.389	0.382	0.382	0	0.156	0.467	0.502	0.505	0.533	0	0.222	0.438	0.394	0.259	0.584	14	0.404642857	0.416	343.5714286
UTS2R	0.421	0.466	0.388	0.388	0	0.114	0.264	0.274	0.278	0.29	0.109	0.131	0.313	0.469	0	0.486	14	0.313642857	0.3015	1594.642857
AKAP12	0.399	0.374	0.196	0.196	0	0	0.385	0.191	0.202	0.226	0.199	0.191	0.417	0.383	0.113	0.449	14	0.280071429	0.214	653.8571429
GNB5	0.394	0.483	0.353	0.353	0	0.103	0.323	0.231	0.236	0	0.661	0.171	0.389	0.486	0.152	0.587	14	0.351571429	0.353	1148.142857
ACKR4	0.39	0.372	0.347	0.347	0.157	0	0.185	0.36	0.363	0.379	0.166	0.129	0.34	0.372	0	0.374	14	0.305785714	0.3535	1758.142857
GPR33	0.388	0.361	0.329	0.329	0.146	0.112	0.275	0.359	0.363	0.345	0	0.144	0.317	0.361	0	0.484	14	0.308071429	0.337	1436.285714
GRK6P1	0.385	0.397	0.323	0.323	0	0	0.221	0.302	0.305	0.298	0.249	0.12	0.394	0.399	0.106	0.493	14	0.308214286	0.314	1617.428571
UNC80	0.374	0.385	0.318	0.318	0	0.123	0.198	0.303	0.305	0.337	0.13	0.103	0.296	0.388	0	0.44	14	0.287	0.3115	2039.214286
RGS11	0.37	0.437	0.299	0.299	0	0.102	0.246	0.26	0.262	0	0.612	0.142	0.339	0.438	0.108	0.5	14	0.315285714	0.299	1695.285714
HRH2	0.354	0.426	0.34	0.339	0	0.101	0.19	0.142	0.146	0.15	0.235	0.104	0.253	0.428	0	0.429	14	0.259785714	0.244	2224

AP2M1	0.348	0.295	0.311	0.311	0	0.144	0.353	0.391	0.395	0.42	0	0.174	0.335	0.299	0.107	0.443	14	0.309	0.323	911.6428571
HRH4	0.347	0.394	0.31	0.31	0	0.169	0.281	0.198	0.202	0.207	0.207	0.2	0.244	0.398	0	0.499	14	0.283285714	0.2625	901.2857143
LPAR1	0.341	0.433	0.292	0.292	0	0.109	0.378	0.103	0.108	0	0.543	0.22	0.302	0.439	0.188	0.504	14	0.303714286	0.297	789.7857143
FPR1	0.325	0.356	0.269	0.269	0	0	0.29	0.175	0.181	0.189	0.215	0.14	0.251	0.361	0.126	0.416	14	0.2545	0.26	1259.857143
OR51D1	0.319	0.328	0.257	0.257	0.106	0	0.278	0.218	0.223	0.228	0.161	0.166	0.246	0.33	0	0.439	14	0.254	0.2515	1107.071429
OR52I1	0.319	0.328	0.257	0.257	0.106	0	0.278	0.218	0.223	0.228	0.161	0.166	0.246	0.33	0	0.439	14	0.254	0.2515	1108.071429
OR52M1	0.319	0.328	0.257	0.257	0.106	0	0.278	0.218	0.223	0.228	0.161	0.166	0.246	0.33	0	0.439	14	0.254	0.2515	1105.071429
OR52I2	0.319	0.328	0.257	0.257	0.106	0	0.278	0.218	0.223	0.228	0.161	0.166	0.246	0.33	0	0.439	14	0.254	0.2515	1109.071429
OR6V1	0.319	0.328	0.257	0.257	0.106	0	0.278	0.218	0.223	0.228	0.161	0.166	0.246	0.33	0	0.439	14	0.254	0.2515	1106.071429
TM2D2	0.316	0.363	0.296	0.296	0	0.102	0.231	0.173	0.176	0.203	0.159	0.1	0.226	0.367	0	0.37	14	0.241285714	0.2285	2171.285714
RXFP2	0.292	0.321	0.349	0.349	0.307	0.139	0.25	0.25	0.254	0.262	0	0.167	0.224	0.322	0	0.471	14	0.282642857	0.277	1269.071429
GPR52	0.282	0.309	0.218	0.218	0	0.165	0.414	0.151	0.158	0.132	0.197	0.215	0.22	0.313	0	0.586	14	0.255571429	0.218	524
GPR53P	0.282	0.309	0.218	0.218	0	0.165	0.414	0.151	0.158	0.132	0.197	0.215	0.22	0.313	0	0.586	14	0.255571429	0.218	523
AP2A1	0.277	0.266	0.264	0.264	0	0.154	0.36	0.284	0.287	0.299	0	0.177	0.264	0.269	0.108	0.436	14	0.264928571	0.2675	882.1428571
RXFP3	0.266	0.331	0.258	0.258	0.168	0.169	0.27	0.101	0.106	0.102	0	0.133	0.127	0.334	0	0.475	14	0.221285714	0.2135	1457.642857
GPR18	0.265	0.291	0.196	0.196	0	0.113	0.229	0.14	0.144	0.145	0.16	0.123	0.215	0.293	0	0.377	14	0.206214286	0.196	1971.285714
RGS21	0.261	0.329	0.269	0.269	0	0.168	0.269	0.107	0.111	0.117	0.315	0.207	0.235	0.332	0	0.409	14	0.242714286	0.265	1033.071429
PHLDB2	0.26	0.23	0.238	0.238	0	0.105	0.34	0.272	0.277	0.295	0	0.184	0.259	0.237	0.202	0.382	14	0.251357143	0.2485	1161.642857
GPR12	0.257	0.307	0.232	0.232	0	0.344	0.521	0.117	0.123	0.108	0.259	0.456	0.205	0.315	0	0.57	14	0.289	0.258	192.5714286
AP2S1	0.244	0.299	0.308	0.308	0	0.123	0.309	0.181	0.183	0.218	0	0.139	0.203	0.302	0.138	0.417	14	0.240857143	0.231	1409.785714
GPR34	0.243	0.273	0.193	0.193	0	0.167	0.395	0.18	0.183	0.117	0.197	0.194	0.192	0.277	0	0.523	14	0.237642857	0.1935	670
SCTR	0.242	0.306	0.248	0.248	0.161	0.191	0.351	0.114	0.12	0.13	0	0.197	0.148	0.311	0	0.486	14	0.232357143	0.2195	740.5714286
PDE4C	0.239	0.274	0.243	0.243	0	0.115	0.45	0.198	0.202	0.183	0.149	0.156	0.229	0.28	0	0.483	14	0.246	0.234	968
RPL23AP1	0.233	0.297	0.252	0.252	0	0.128	0.202	0.153	0.16	0.16	0.171	0.153	0.211	0.298	0	0.372	14	0.217285714	0.2065	1787.928571
TCEAL3	0.23	0.21	0.197	0.197	0	0.116	0.435	0.231	0.235	0.236	0	0.213	0.194	0.216	0.164	0.525	14	0.242785714	0.2145	705.6428571
GPR21	0.22	0.243	0.176	0.176	0	0.149	0.358	0.151	0.156	0.129	0.132	0.296	0.178	0.248	0	0.461	14	0.2195	0.177	610.6428571
ARFIP1	0.214	0.228	0.199	0.199	0	0	0.39	0.206	0.208	0.221	0.145	0.156	0.272	0.237	0.317	0.364	14	0.239714286	0.2175	957.5714286
PDE4B	0.209	0.25	0.198	0.198	0	0.113	0.402	0.155	0.162	0.153	0.129	0.177	0.231	0.258	0	0.404	14	0.217071429	0.198	1024.142857
PDE8A	0.206	0.239	0.181	0.181	0	0.11	0.392	0.158	0.163	0.145	0.124	0.239	0.203	0.246	0	0.451	14	0.217	0.192	785
VN2R1P	0.205	0.277	0.173	0.173	0	0.11	0.202	0.116	0.118	0.117	0.168	0.103	0.177	0.278	0	0.391	14	0.186285714	0.173	2282

FLNA	0.192	0.194	0.17	0.17	0	0.193	0.291	0.181	0.184	0.196	0	0.239	0.161	0.199	0.22	0.361	14	0.210785714	0.1935	946.2857143
RAB22A	0.189	0.172	0.113	0.113	0	0.336	0.323	0.131	0.135	0.135	0	0.307	0.144	0.174	0.109	0.341	14	0.194428571	0.158	910.1428571
WDR66	0.187	0.166	0.107	0.107	0.132	0.115	0.24	0.138	0.145	0.13	0	0.14	0.155	0.168	0	0.303	14	0.1595	0.1425	2147.071429
ANKRD13C	0.187	0.236	0.186	0.186	0	0.198	0.437	0.106	0.112	0.103	0.137	0.256	0.126	0.241	0	0.459	14	0.212142857	0.1865	462.5
CHMP4B	0.187	0.181	0.131	0.131	0	0.186	0.494	0.164	0.167	0.18	0	0.262	0.17	0.187	0.113	0.483	14	0.216857143	0.1805	435.8571429
CLTA	0.185	0.175	0.14	0.14	0	0.128	0.368	0.194	0.197	0.208	0	0.15	0.208	0.178	0.132	0.374	14	0.198357143	0.1815	1302.357143
GPR108	0.182	0.209	0.178	0.178	0	0.159	0.52	0.116	0.12	0.11	0.153	0.21	0.158	0.213	0	0.563	14	0.219214286	0.178	561.2142857
GUCA1C	0.181	0.176	0.107	0.107	0	0.158	0.273	0.345	0.347	0.112	0.107	0.182	0.16	0.177	0	0.364	14	0.199714286	0.1765	1393.785714
WDR65	0.179	0.187	0.148	0.148	0	0.164	0.506	0.105	0.112	0.101	0.219	0.285	0.16	0.193	0	0.512	14	0.215642857	0.1715	414.2142857
NECAP2	0.178	0.162	0.144	0.144	0	0.11	0.341	0.135	0.137	0.156	0	0.144	0.129	0.165	0.124	0.376	14	0.174642857	0.144	1571.714286
DENND1B	0.178	0.16	0.148	0.148	0	0.107	0.454	0.217	0.224	0.197	0	0.202	0.163	0.162	0.171	0.459	14	0.213571429	0.1745	884.6428571
CLTCL1	0.176	0.163	0.153	0.153	0	0.127	0.406	0.183	0.186	0.187	0	0.191	0.181	0.165	0.116	0.432	14	0.201357143	0.1785	940.7857143
TBC1D24	0.172	0.176	0.154	0.154	0	0.104	0.328	0.144	0.145	0.155	0	0.139	0.183	0.178	0.212	0.357	14	0.185785714	0.1635	1653.142857
CLINT1	0.171	0.164	0.136	0.136	0	0.149	0.45	0.152	0.155	0.161	0	0.193	0.162	0.169	0.128	0.422	14	0.196285714	0.1615	810.0714286
AP1G1	0.167	0.16	0.139	0.139	0	0.181	0.445	0.158	0.161	0.172	0	0.203	0.154	0.165	0.143	0.438	14	0.201785714	0.163	682.8571429
SMAP1	0.166	0.156	0.106	0.106	0	0.118	0.344	0.179	0.181	0.141	0	0.173	0.145	0.16	0.236	0.382	14	0.185214286	0.163	1312.714286
CLTB	0.164	0.149	0.127	0.127	0	0.107	0.388	0.184	0.187	0.19	0	0.124	0.18	0.155	0.109	0.352	14	0.181642857	0.1595	1681.071429
AP1S1	0.162	0.158	0.139	0.139	0	0.164	0.418	0.149	0.152	0.162	0	0.212	0.152	0.164	0.166	0.411	14	0.196285714	0.162	738.3571429
SCYL2	0.159	0.16	0.119	0.119	0	0.14	0.401	0.127	0.13	0.132	0	0.275	0.133	0.164	0.118	0.424	14	0.185785714	0.1365	733.0714286
DNM2	0.156	0.175	0.115	0.115	0	0.153	0.39	0.109	0.111	0.116	0	0.196	0.137	0.182	0.195	0.382	14	0.180857143	0.1545	963.6428571
SNAP91	0.154	0.154	0.118	0.118	0	0.19	0.502	0.132	0.136	0.145	0	0.195	0.148	0.159	0.104	0.431	14	0.191857143	0.151	747
PPP1CB	0.141	0.148	0.143	0.143	0	0	0.445	0.115	0.118	0.117	0.1	0.233	0.135	0.162	0.168	0.403	14	0.183642857	0.143	666.4285714
AP1M1	0.136	0.123	0.117	0.117	0	0.138	0.267	0.138	0.139	0.154	0	0.111	0.131	0.126	0.101	0.293	14	0.149357143	0.1335	2286.785714
AP1S2	0.134	0.126	0.117	0.117	0	0.175	0.418	0.135	0.137	0.143	0	0.186	0.127	0.131	0.18	0.395	14	0.180071429	0.136	953.3571429
AP1M2	0.132	0.134	0.121	0.12	0	0.216	0.432	0.113	0.116	0.128	0	0.23	0.129	0.139	0.104	0.413	14	0.1805	0.1305	730.2142857
AP1S3	0.124	0.117	0.103	0.103	0	0.146	0.343	0.127	0.129	0.136	0	0.161	0.12	0.122	0.205	0.333	14	0.162071429	0.128	1514.142857
ADRBK2	0.71	0.713	0.668	0.668	0	0	0.179	0.585	0.588	0.611	0.359	0	0.716	0.714	0.139	0.602	13	0.557846154	0.611	1046.769231
AP2B1	0.699	0.572	0.624	0.624	0	0	0.236	0.832	0.833	0.875	0	0.113	0.662	0.574	0.137	0.551	13	0.564	0.624	1635.461538
OR2AG1	0.662	0.584	0.59	0.59	0	0	0.239	0.728	0.732	0.75	0.122	0.116	0.643	0.585	0	0.575	13	0.532	0.59	1601.692308
RBM26	0.604	0.517	0.529	0.529	0	0	0.15	0.737	0.737	0.692	0.132	0	0.621	0.517	0.103	0.477	13	0.488076923	0.529	1250.538462

RGS3	0.444	0.554	0.406	0.406	0.122	0.103	0.339	0	0	0	0.761	0.235	0.385	0.558	0.245	0.612	13	0.397692308	0.406	865.5384615
RGS16	0.43	0.543	0.436	0.436	0	0	0.254	0.131	0.133	0	0.823	0.185	0.412	0.546	0.151	0.544	13	0.386461538	0.43	1106.076923
RGS7	0.421	0.518	0.4	0.4	0	0	0.274	0.171	0.174	0	0.777	0.141	0.423	0.521	0.137	0.555	13	0.377846154	0.4	1297.538462
GNGT2	0.416	0.441	0.339	0.339	0	0	0.188	0.447	0.45	0.254	0.393	0	0.415	0.442	0.12	0.465	13	0.362230769	0.415	1064.307692
TMEM181	0.407	0.354	0.362	0.362	0	0.117	0.277	0.446	0.449	0.448	0	0.163	0.383	0.354	0	0.463	13	0.352692308	0.362	1345.153846
EIF3J	0.406	0.329	0.365	0.365	0	0	0.396	0.494	0.497	0.519	0	0.199	0.381	0.333	0.113	0.514	13	0.377769231	0.381	543.6923077
RGS1	0.402	0.508	0.393	0.392	0.124	0.108	0.319	0	0	0	0.687	0.229	0.358	0.511	0.199	0.567	13	0.369	0.392	933.3076923
LPHN1	0.398	0.513	0.443	0.442	0.16	0.126	0.277	0	0	0	0.828	0.144	0.397	0.514	0.156	0.533	13	0.379307692	0.398	1454.076923
PDXP	0.378	0.334	0.329	0.329	0	0	0.174	0.448	0.449	0.477	0	0.121	0.346	0.335	0.405	0.328	13	0.342538462	0.335	2095.769231
GRK4	0.362	0.414	0.27	0.27	0	0	0.117	0.233	0.236	0.227	0.23	0	0.457	0.415	0.119	0.361	13	0.285461538	0.27	1544.461538
RGS9	0.341	0.36	0.236	0.236	0	0	0.242	0.364	0.367	0.18	0.282	0.124	0.307	0.362	0	0.451	13	0.296307692	0.307	1577.307692
P2RY1	0.314	0.381	0.301	0.301	0	0.114	0.127	0.126	0.128	0.141	0.361	0	0.268	0.382	0	0.336	13	0.252307692	0.301	1835.692308
BPNT1	0.313	0.287	0.291	0.291	0	0.164	0.265	0.328	0.331	0.363	0	0.197	0.298	0.291	0	0.386	13	0.292692308	0.291	1139.076923
DENND1C	0.306	0.275	0.274	0.274	0	0	0.355	0.346	0.349	0.37	0	0.209	0.28	0.279	0.285	0.457	13	0.312230769	0.285	595
WDR26	0.3	0.363	0.268	0.268	0	0	0.326	0.107	0.112	0.113	0.499	0.193	0.337	0.367	0	0.47	13	0.286384615	0.3	814.1538462
HCAR2	0.29	0.332	0.264	0.263	0	0	0.253	0.171	0.175	0.171	0.222	0.189	0.241	0.337	0	0.358	13	0.251230769	0.253	1250.538462
PDC	0.283	0.28	0.203	0.203	0	0	0.312	0.351	0.355	0.2	0.167	0.12	0.283	0.285	0	0.411	13	0.265615385	0.283	1396.230769
SLC6A7	0.279	0.261	0.173	0.172	0	0.131	0.228	0.153	0.163	0.163	0	0	0.243	0.261	0.16	0.381	13	0.212923077	0.173	1263.692308
FPR2	0.276	0.309	0.244	0.244	0	0	0.224	0.123	0.127	0.136	0.115	0.116	0.16	0.311	0	0.361	13	0.211230769	0.224	1975.615385
ZSWIM8	0.271	0.368	0.299	0.299	0.101	0.147	0.266	0	0	0	0.722	0.197	0.292	0.368	0.122	0.446	13	0.299846154	0.292	1187.307692
CDRT15P2	0.271	0.368	0.299	0.299	0.101	0.147	0.266	0	0	0	0.722	0.197	0.292	0.368	0.122	0.446	13	0.299846154	0.292	1189.307692
NRG3-AS1	0.271	0.368	0.299	0.299	0.101	0.147	0.266	0	0	0	0.722	0.197	0.292	0.368	0.122	0.446	13	0.299846154	0.292	1188.307692
FAM65C	0.271	0.368	0.299	0.299	0.101	0.147	0.266	0	0	0	0.722	0.197	0.292	0.368	0.122	0.446	13	0.299846154	0.292	1186.307692
GPR26	0.257	0.285	0.209	0.209	0	0	0.352	0.141	0.145	0.145	0.172	0.154	0.205	0.289	0	0.432	13	0.230384615	0.209	1029.307692
ANKRD30BL	0.248	0.34	0.272	0.272	0.124	0.161	0.326	0	0	0	0.634	0.218	0.276	0.341	0.107	0.485	13	0.292615385	0.272	816.6153846
FRMPD4	0.247	0.237	0.335	0.335	0	0	0.229	0.19	0.191	0.206	0.127	0	0.195	0.24	0.151	0.352	13	0.233461538	0.229	1143.307692
INPP5A	0.241	0.264	0.222	0.222	0	0	0.326	0.252	0.254	0.204	0.127	0.12	0.238	0.268	0	0.395	13	0.241	0.241	1400.461538
WLS	0.24	0.232	0.199	0.199	0.102	0	0.145	0.252	0.253	0.277	0	0.153	0.205	0.233	0	0.281	13	0.213153846	0.232	2277.846154
OR8D4	0.235	0.264	0.19	0.19	0	0.161	0.218	0.131	0.135	0.144	0.141	0	0.213	0.266	0	0.405	13	0.207153846	0.19	1185.538462
C6ORF25	0.233	0.2	0.164	0.164	0	0.132	0.239	0.168	0.172	0.192	0	0.157	0.189	0.204	0	0.278	13	0.191692308	0.189	2047.076923

YIPF6	0.219	0.218	0.204	0.204	0	0.17	0.18	0.228	0.23	0.235	0	0.177	0.202	0.219	0	0.323	13	0.216076923	0.218	1848.230769
PDE2A	0.214	0.269	0.167	0.167	0	0.19	0.339	0.118	0.124	0	0.206	0.205	0.186	0.274	0	0.402	13	0.220076923	0.205	871.9230769
SLC16A8	0.205	0.197	0.159	0.159	0	0.134	0.248	0.29	0.292	0.181	0	0.103	0.188	0.199	0	0.346	13	0.207769231	0.197	2159
OR1D5	0.203	0.234	0.182	0.181	0	0.175	0.439	0.121	0.126	0	0.146	0.206	0.154	0.238	0	0.537	13	0.226307692	0.182	617.6153846
GPR113	0.203	0.24	0.176	0.176	0	0	0.327	0.124	0.128	0.117	0.136	0.225	0.155	0.244	0	0.472	13	0.209461538	0.176	775.6923077
ANO7	0.201	0.187	0.214	0.214	0	0.19	0.334	0.218	0.222	0.233	0	0.214	0.196	0.187	0	0.403	13	0.231769231	0.214	809.3076923
CATSPERG	0.201	0.216	0.232	0.232	0	0.173	0	0.221	0.224	0.24	0.112	0.111	0.199	0.216	0	0.283	13	0.204615385	0.216	1556.384615
SSNA1	0.197	0.182	0.165	0.165	0	0.155	0.508	0.211	0.217	0.228	0	0.219	0.178	0.189	0	0.47	13	0.237230769	0.197	528.2307692
NALCN	0.197	0.223	0.12	0.12	0	0.151	0.183	0.121	0.123	0.136	0	0.134	0.111	0.224	0	0.336	13	0.167615385	0.136	2284.307692
ACKR2	0.197	0.213	0.174	0.174	0	0.116	0.235	0.159	0.163	0.167	0	0.163	0.146	0.215	0	0.34	13	0.189384615	0.174	1885.692308
CETN4P	0.185	0.184	0.109	0.109	0	0	0.318	0.272	0.275	0.109	0.136	0.137	0.174	0.191	0	0.355	13	0.196461538	0.184	1524.307692
AP2A2	0.178	0.179	0.195	0.195	0	0.163	0.412	0.175	0.178	0.189	0	0.188	0.162	0.184	0	0.396	13	0.214923077	0.184	830.3076923
AAK1	0.174	0.189	0.132	0.132	0	0.155	0.41	0.148	0.152	0.157	0	0.212	0.172	0.195	0	0.434	13	0.204769231	0.172	739
EPN1	0.174	0.171	0.122	0.122	0	0.11	0.377	0.111	0.114	0.149	0	0.203	0.134	0.174	0	0.389	13	0.180769231	0.149	1172.615385
WDR20	0.174	0.187	0.161	0.161	0	0	0.384	0.102	0.104	0.117	0.232	0.25	0.175	0.191	0	0.442	13	0.206153846	0.175	589.9230769
GPR111	0.171	0.205	0.14	0.14	0	0.177	0.335	0.114	0.119	0.119	0	0.186	0.126	0.206	0	0.421	13	0.189153846	0.171	1052.076923
REP15	0.168	0.169	0.104	0.104	0	0.17	0.455	0.116	0.121	0.133	0	0.252	0.149	0.174	0	0.42	13	0.195	0.168	613.5384615
GPR75	0.168	0.205	0.135	0.135	0	0.203	0.382	0.115	0.119	0	0.164	0.209	0.128	0.208	0	0.483	13	0.204153846	0.168	750.3076923
AFTPH	0.164	0.158	0.131	0.13	0	0.125	0.385	0.132	0.134	0.142	0	0.194	0.125	0.163	0	0.42	13	0.184846154	0.142	1065.461538
PDE4A	0.162	0.196	0.191	0.191	0	0	0.324	0.129	0.134	0.136	0.105	0.158	0.156	0.204	0	0.332	13	0.186	0.162	1401.153846
VPS25	0.157	0.151	0.118	0.118	0	0.191	0.391	0.139	0.14	0.167	0	0.298	0.12	0.154	0	0.397	13	0.195461538	0.154	655.6153846
AP4B1	0.153	0.138	0.122	0.122	0	0.136	0.361	0.177	0.18	0.182	0	0.134	0.138	0.14	0	0.39	13	0.182538462	0.14	1526.769231
ZNF43	0.148	0.17	0.133	0.133	0	0	0.381	0.126	0.13	0.113	0.107	0.224	0.165	0.177	0	0.409	13	0.185846154	0.148	774.5384615
GPR115	0.146	0.189	0.139	0.139	0	0.149	0.312	0.101	0.104	0.113	0	0.166	0.108	0.191	0	0.377	13	0.171846154	0.146	1421.076923
PSD	0.146	0.163	0.12	0.12	0	0.124	0.366	0	0	0.102	0.1	0.202	0.118	0.168	0.479	0.341	13	0.196076923	0.146	1272.461538
INPP5J	0.145	0.151	0.125	0.125	0	0	0.291	0.113	0.117	0.128	0	0.194	0.133	0.158	0.181	0.311	13	0.167076923	0.145	1451.230769
SDS	0.144	0.151	0.152	0.152	0	0.108	0.431	0.164	0.167	0.147	0	0.164	0.125	0.154	0	0.379	13	0.187538462	0.152	1293
TCEAL4	0.141	0.135	0.141	0.141	0.27	0	0.226	0.116	0.119	0.132	0	0.115	0.124	0.139	0	0.27	13	0.159153846	0.139	2440.846154
PAIP2	0.141	0.141	0.109	0.109	0	0.108	0.594	0.118	0.122	0.141	0	0.297	0.109	0.148	0	0.525	13	0.204769231	0.141	757.3076923
PITPNM3	0.139	0.177	0.113	0.113	0	0	0.325	0.132	0.137	0	0.162	0.204	0.118	0.184	0.279	0.392	13	0.190384615	0.162	1047.384615

HIST2H2BD	0.138	0.157	0.141	0.141	0.299	0.113	0.299	0.187	0.189	0	0	0.141	0.11	0.159	0	0.361	13	0.187307692	0.157	1829.538462
SYNRG	0.132	0.132	0.111	0.111	0	0.139	0.439	0.124	0.128	0.135	0	0.205	0.122	0.137	0	0.407	13	0.178615385	0.132	944.3846154
ARRDC4	0.127	0.121	0.114	0.114	0	0.126	0.257	0.148	0.151	0.136	0	0.155	0.111	0.122	0	0.277	13	0.150692308	0.127	2234.461538
Mar-02	0.609	0.482	0.463	0.463	0	0	0.196	0.651	0.656	0.716	0	0.137	0.577	0.481	0	0.461	12	0.491	0.4815	1742.25
GDA	0.52	0.432	0.446	0.446	0	0	0.308	0.603	0.604	0.608	0	0.115	0.481	0.435	0	0.517	12	0.459583333	0.4635	1387.333333
USP20	0.423	0.326	0.31	0.31	0	0	0.237	0.429	0.433	0.483	0	0.174	0.38	0.327	0	0.372	12	0.350333333	0.3495	1407.916667
GPSM3	0.412	0.517	0.415	0.415	0	0	0.27	0	0.102	0	0.761	0.159	0.375	0.519	0.192	0.536	12	0.389416667	0.4135	1260.166667
GPSM1	0.403	0.514	0.373	0.373	0	0.111	0.374	0	0	0	0.741	0.226	0.35	0.519	0.183	0.619	12	0.398833333	0.3735	787.75
GTF3A	0.392	0.338	0.331	0.331	0	0	0.281	0.441	0.444	0.442	0	0.169	0.342	0.34	0	0.453	12	0.358666667	0.341	1113.333333
RGS4	0.392	0.526	0.42	0.42	0	0.105	0.26	0	0	0	0.787	0.169	0.376	0.527	0.148	0.541	12	0.38925	0.406	1589.416667
LPAR2	0.363	0.47	0.332	0.332	0	0.24	0.335	0	0	0	0.667	0.349	0.335	0.475	0.179	0.507	12	0.382	0.342	459.8333333
GNG3	0.349	0.467	0.375	0.374	0	0.126	0.416	0	0	0	0.643	0.242	0.329	0.471	0.137	0.632	12	0.380083333	0.3745	566.8333333
USP33	0.339	0.264	0.271	0.271	0	0	0.234	0.356	0.36	0.396	0	0.173	0.298	0.265	0	0.335	12	0.296833333	0.2845	1563.833333
S1PR5	0.322	0.427	0.288	0.288	0	0.167	0.353	0	0	0	0.471	0.262	0.27	0.432	0.198	0.529	12	0.333916667	0.305	532.75
CCER1	0.31	0.256	0.256	0.256	0	0	0.218	0.362	0.363	0.379	0	0.142	0.289	0.257	0	0.385	12	0.289416667	0.273	1759.916667
GPR56	0.304	0.285	0.269	0.269	0	0	0.27	0.311	0.313	0.333	0	0.152	0.293	0.286	0	0.373	12	0.288166667	0.2895	1440.583333
ZNF418	0.299	0.255	0.269	0.269	0	0	0.175	0.328	0.328	0.35	0	0.159	0.307	0.257	0	0.321	12	0.276416667	0.284	2016.083333
ZNF322	0.297	0.257	0.263	0.263	0	0	0.177	0.324	0.324	0.342	0	0.16	0.306	0.259	0	0.323	12	0.274583333	0.28	1990.083333
GPR139	0.294	0.379	0.287	0.287	0.108	0.142	0.327	0	0	0	0.475	0.225	0.272	0.381	0	0.537	12	0.3095	0.2905	811.4166667
APLNR	0.28	0.328	0.23	0.23	0	0	0.197	0.218	0.221	0.239	0	0.128	0.207	0.33	0	0.343	12	0.245916667	0.23	2109.916667
GNG5P1	0.275	0.357	0.286	0.286	0	0.125	0.343	0	0	0	0.522	0.129	0.278	0.358	0.109	0.551	12	0.301583333	0.286	1495.583333
RGS9BP	0.274	0.262	0.161	0.161	0	0	0.184	0.437	0.438	0.167	0.169	0	0.25	0.263	0	0.332	12	0.258166667	0.256	1464
GPR85	0.268	0.264	0.24	0.24	0	0	0.345	0.23	0.233	0.228	0	0.202	0.223	0.267	0	0.452	12	0.266	0.24	734.75
PPP2R4	0.262	0.236	0.229	0.228	0	0	0.567	0.286	0.291	0.315	0	0.304	0.248	0.252	0	0.489	12	0.308916667	0.274	164
NTS	0.257	0.297	0.229	0.229	0	0	0.287	0.168	0.171	0.175	0	0.11	0.167	0.301	0	0.421	12	0.234333333	0.229	1696.916667
P2RY12	0.253	0.291	0.202	0.202	0	0	0.114	0.12	0.124	0.132	0.204	0	0.208	0.292	0	0.249	12	0.19925	0.203	2175.166667
TMEM237	0.25	0.217	0.15	0.15	0	0	0.256	0.449	0.45	0.223	0	0.139	0.248	0.219	0	0.386	12	0.261416667	0.2355	1672.75
PDE8B	0.25	0.299	0.367	0.367	0.575	0.112	0.194	0	0	0	0.367	0.103	0.154	0.3	0	0.373	12	0.288416667	0.2995	2550.666667
AMPD2	0.25	0.362	0.307	0.306	0	0.103	0.352	0	0	0	0.663	0.181	0.272	0.364	0.135	0.53	12	0.31875	0.3065	1205.25
RAB11FIP1	0.244	0.285	0.215	0.214	0	0.21	0.409	0	0	0	0.21	0.228	0.16	0.291	0.105	0.519	12	0.2575	0.2215	557.3333333

ADRA1B	0.239	0.312	0.223	0.222	0	0	0.115	0.131	0.137	0.125	0.202	0	0.317	0.313	0	0.272	12	0.217333333	0.2225	2032.5
GRK7	0.239	0.234	0.12	0.12	0	0	0.159	0.425	0.426	0.132	0.128	0	0.234	0.235	0	0.281	12	0.22775	0.234	1862.166667
RCVRN	0.237	0.242	0.127	0.127	0	0	0.226	0.364	0.366	0.117	0.18	0	0.263	0.244	0	0.318	12	0.23425	0.2395	1355.833333
S1PR3	0.237	0.336	0.196	0.195	0	0.129	0.289	0	0	0	0.312	0.213	0.218	0.341	0.353	0.39	12	0.267416667	0.263	1188.666667
OPN5	0.235	0.255	0.16	0.16	0	0	0.147	0.305	0.307	0.116	0.179	0	0.196	0.255	0	0.31	12	0.21875	0.2155	1741.416667
PDE10A	0.234	0.305	0.241	0.241	0.374	0.109	0.266	0	0	0	0.321	0.112	0.172	0.307	0	0.41	12	0.257666667	0.2535	2094
NTSR2	0.226	0.251	0.231	0.231	0	0.106	0.243	0.111	0.113	0.109	0	0	0.108	0.254	0	0.369	12	0.196	0.2285	1502.25
ADAP1	0.219	0.274	0.156	0.156	0.104	0	0.432	0	0	0	0.198	0.19	0.117	0.282	0.184	0.441	12	0.229416667	0.194	737.25
GPR174	0.218	0.219	0.176	0.176	0	0	0.157	0.135	0.137	0.132	0.137	0	0.162	0.22	0	0.258	12	0.17725	0.169	1994.25
IMPAD1	0.218	0.258	0.239	0.239	0.101	0.134	0.243	0	0	0	0.359	0.193	0.22	0.259	0	0.352	12	0.234583333	0.239	1622.166667
HTR4	0.217	0.252	0.176	0.176	0	0.119	0.236	0.111	0.118	0	0	0.109	0.145	0.254	0	0.366	12	0.189916667	0.176	2395.75
GUCY2F	0.214	0.207	0.118	0.118	0	0	0.216	0.399	0.402	0.147	0	0.103	0.198	0.208	0	0.345	12	0.222916667	0.2075	2338.5
GBF1	0.213	0.223	0.109	0.109	0	0.182	0.52	0	0	0	0.139	0.272	0.151	0.23	0.239	0.512	12	0.241583333	0.218	423.4166667
DHX8	0.207	0.179	0.183	0.183	0	0	0.457	0.245	0.248	0.256	0	0.2	0.191	0.182	0	0.509	12	0.253333333	0.2035	550.75
TRNAV21	0.205	0.177	0.177	0.177	0	0	0.279	0.23	0.233	0.233	0	0.153	0.208	0.179	0	0.329	12	0.215	0.2065	1637.083333
TRNAV32	0.205	0.177	0.177	0.177	0	0	0.279	0.23	0.233	0.233	0	0.153	0.208	0.179	0	0.329	12	0.215	0.2065	1639.083333
TRNAR2	0.205	0.177	0.177	0.177	0	0	0.279	0.23	0.233	0.233	0	0.153	0.208	0.179	0	0.329	12	0.215	0.2065	1638.083333
EMR2	0.204	0.229	0.152	0.152	0.13	0.227	0.37	0	0	0	0.162	0.227	0.125	0.232	0	0.441	12	0.220916667	0.2155	709.5833333
SPHK2	0.204	0.272	0.147	0.147	0	0.124	0.431	0	0	0	0.25	0.27	0.187	0.28	0.118	0.415	12	0.237083333	0.227	701.5
S1PR2	0.203	0.293	0.168	0.168	0	0.136	0.306	0	0	0	0.311	0.235	0.186	0.298	0.227	0.372	12	0.241916667	0.231	1085.083333
ELTD1	0.202	0.205	0.149	0.149	0	0.122	0.193	0.113	0.115	0	0	0.142	0.12	0.207	0	0.291	12	0.167333333	0.149	2622.333333
S1PR1	0.202	0.29	0.17	0.17	0	0.136	0.309	0	0	0	0.249	0.208	0.182	0.295	0.24	0.366	12	0.23475	0.224	1191.166667
GPR89A	0.2	0.255	0.186	0.186	0.43	0.31	0.24	0	0	0	0.186	0.232	0.102	0.257	0	0.36	12	0.245333333	0.236	1291.5
ZNF326	0.199	0.165	0.19	0.19	0	0	0.236	0.223	0.225	0.237	0	0.135	0.175	0.166	0	0.289	12	0.2025	0.1945	2163.833333
PLEK	0.199	0.236	0.139	0.139	0	0.131	0.486	0	0	0	0.19	0.232	0.199	0.247	0.201	0.479	12	0.239833333	0.2	626.3333333
C16ORF89	0.198	0.258	0.26	0.26	0.607	0.151	0.234	0	0	0	0.364	0.142	0.147	0.257	0	0.372	12	0.270833333	0.2575	1875.833333
OR51E1	0.197	0.232	0.173	0.173	0	0.173	0.35	0	0.1	0	0.136	0.248	0.139	0.234	0	0.434	12	0.21575	0.185	767.3333333
CYTH3	0.196	0.221	0.174	0.174	0	0.143	0.463	0	0	0	0.165	0.263	0.141	0.229	0.262	0.5	12	0.24425	0.2085	498.8333333
NSF	0.195	0.164	0.118	0.118	0	0.103	0.221	0.177	0.18	0.185	0	0	0.183	0.165	0	0.289	12	0.174833333	0.1785	1972.166667
CYTH1	0.189	0.213	0.157	0.156	0	0.131	0.32	0	0	0	0.178	0.182	0.129	0.219	0.219	0.379	12	0.206	0.1855	1358.25

STARD3NL	0.188	0.162	0.131	0.131	0	0	0.281	0.167	0.174	0.178	0	0.141	0.161	0.165	0	0.321	12	0.183333333	0.166	1838.083333
SNX21	0.185	0.221	0.133	0.133	0	0.132	0.473	0	0	0	0.128	0.29	0.122	0.229	0.261	0.497	12	0.233666667	0.203	560.6666667
SPHK1	0.184	0.253	0.131	0.131	0	0.117	0.451	0	0	0	0.241	0.285	0.176	0.262	0.132	0.433	12	0.233	0.2125	692.5833333
GPR144	0.182	0.17	0.193	0.193	0	0	0.183	0.157	0.159	0.158	0	0.122	0.145	0.17	0	0.331	12	0.18025	0.17	2405.25
GPR32P1	0.182	0.2	0.182	0.182	0	0	0.368	0.103	0.108	0	0.133	0.159	0.177	0.203	0	0.455	12	0.204333333	0.182	1101.25
RAPGEF3	0.179	0.243	0.115	0.115	0	0.118	0.346	0	0	0	0.153	0.197	0.156	0.251	0.172	0.326	12	0.197583333	0.1755	1449.25
DGKE	0.177	0.224	0.141	0.14	0	0.112	0.441	0	0	0	0.322	0.23	0.173	0.232	0.105	0.39	12	0.223916667	0.2005	944.9166667
AGAP1	0.177	0.194	0.137	0.136	0	0.143	0.508	0	0	0	0.179	0.249	0.118	0.203	0.228	0.475	12	0.228916667	0.1865	565.6666667
GPR82	0.172	0.179	0.142	0.141	0	0	0.262	0.118	0.12	0.118	0	0.196	0.13	0.18	0	0.3	12	0.1715	0.157	1648.666667
PIP5K1C	0.171	0.196	0.134	0.134	0	0.127	0.415	0	0	0	0.149	0.245	0.137	0.206	0.317	0.406	12	0.21975	0.1835	801.1666667
SNX27	0.17	0.198	0.13	0.129	0	0.195	0.422	0	0	0	0.124	0.228	0.137	0.204	0.224	0.438	12	0.216583333	0.1965	657
DENND1A	0.165	0.189	0.236	0.236	0.445	0	0.206	0.129	0.131	0.134	0	0	0.112	0.19	0	0.309	12	0.206833333	0.1895	1529.75
NAT8L	0.165	0.161	0.13	0.13	0	0	0.362	0.146	0.148	0.147	0	0.129	0.132	0.166	0	0.361	12	0.181416667	0.1475	1542.666667
OXR1	0.165	0.171	0.171	0.171	0	0	0.268	0.188	0.19	0.193	0	0.137	0.123	0.172	0	0.309	12	0.188166667	0.1715	1950.916667
ARFGAP1	0.162	0.192	0.131	0.131	0	0.156	0.53	0	0	0	0.207	0.247	0.138	0.199	0.186	0.497	12	0.231333333	0.189	522.5
CATSPERB	0.161	0.165	0.145	0.145	0	0	0.191	0.158	0.162	0.166	0	0.143	0.162	0.166	0	0.272	12	0.169666667	0.162	2461.5
GPR50	0.16	0.181	0.177	0.177	0.137	0	0.239	0.113	0.117	0	0	0.144	0.112	0.182	0	0.361	12	0.175	0.1685	1918.166667
OR4E1	0.16	0.173	0.127	0.127	0	0.175	0.359	0.141	0.146	0	0	0.21	0.12	0.177	0	0.463	12	0.198166667	0.1665	909.5833333
OR52Z1	0.16	0.173	0.127	0.127	0	0.175	0.359	0.141	0.146	0	0	0.21	0.12	0.177	0	0.463	12	0.198166667	0.1665	908.5833333
OR10J4	0.16	0.173	0.127	0.127	0	0.175	0.359	0.141	0.146	0	0	0.21	0.12	0.177	0	0.463	12	0.198166667	0.1665	910.5833333
OR5AC1	0.16	0.173	0.127	0.127	0	0.175	0.359	0.141	0.146	0	0	0.21	0.12	0.177	0	0.463	12	0.198166667	0.1665	911.5833333
COX7B	0.159	0.134	0.148	0.148	0	0	0.277	0.191	0.194	0.197	0	0.137	0.156	0.137	0	0.322	12	0.183333333	0.1575	1919.583333
ESRRAP2	0.159	0.182	0.149	0.149	0	0	0.18	0.104	0.106	0	0.208	0.14	0.189	0.182	0	0.346	12	0.1745	0.1695	2276.083333
ESRRAP1	0.159	0.182	0.149	0.149	0	0	0.18	0.104	0.106	0	0.208	0.14	0.189	0.182	0	0.346	12	0.1745	0.1695	2277.083333
ARFGAP3	0.155	0.173	0.116	0.116	0	0.159	0.474	0	0	0	0.171	0.226	0.114	0.179	0.181	0.452	12	0.209666667	0.172	689.0833333
RAB4A	0.155	0.174	0.104	0.104	0	0.291	0.351	0	0	0	0.112	0.279	0.151	0.179	0.171	0.358	12	0.202416667	0.1725	904.8333333
RAB11A	0.154	0.194	0.123	0.123	0	0.253	0.396	0	0	0	0.143	0.274	0.126	0.199	0.132	0.412	12	0.21075	0.174	642.1666667
PDE11A	0.152	0.177	0.186	0.186	0	0.105	0.269	0.108	0.11	0	0.166	0.146	0	0.18	0	0.347	12	0.177666667	0.1715	2122.916667
RNU3P1	0.151	0.147	0.136	0.136	0	0	0.28	0.161	0.165	0.167	0	0.127	0.187	0.149	0	0.353	12	0.179916667	0.156	1876.416667
PRKAR2B	0.15	0.202	0.154	0.154	0.343	0.141	0.4	0	0	0	0.156	0.161	0.156	0.209	0	0.382	12	0.217333333	0.1585	1203.583333

COPZ2	0.149	0.195	0.143	0.143	0	0.178	0.533	0	0	0	0.25	0.245	0.158	0.2	0.146	0.517	12	0.238083333	0.1865	466.4166667
COPG1	0.148	0.179	0.124	0.124	0	0.193	0.483	0	0	0	0.172	0.23	0.133	0.185	0.249	0.486	12	0.2255	0.182	549.6666667
TCEA1	0.147	0.116	0.103	0.103	0	0	0.385	0.141	0.144	0.15	0	0.184	0.115	0.12	0	0.433	12	0.178416667	0.1425	1099.083333
GOLPH3	0.147	0.132	0	0	0	0.155	0.476	0.152	0.158	0.119	0	0.287	0.103	0.139	0.11	0.454	12	0.202666667	0.1495	602.5
GNG13	0.147	0.212	0.168	0.168	0	0.143	0.293	0	0	0	0.291	0.143	0.134	0.214	0.106	0.394	12	0.201083333	0.168	1726.083333
RAB5A	0.147	0.171	0.106	0.106	0	0.209	0.457	0	0	0	0.111	0.254	0.12	0.175	0.172	0.432	12	0.205	0.1715	626.1666667
EPS15	0.146	0.147	0	0	0	0.123	0.419	0.102	0.105	0.123	0	0.205	0.109	0.151	0.116	0.429	12	0.18125	0.1345	1067.25
SNX33	0.146	0.147	0	0	0	0.154	0.516	0.103	0.108	0.115	0	0.237	0.125	0.156	0.17	0.405	12	0.1985	0.1505	687
ARFGEF2	0.146	0.169	0.114	0.114	0	0.174	0.468	0	0	0	0.158	0.237	0.126	0.175	0.164	0.456	12	0.208416667	0.1665	644
ARFGEF1	0.144	0.163	0.121	0.121	0	0.178	0.573	0	0	0	0.194	0.264	0.122	0.171	0.135	0.489	12	0.222916667	0.167	510.0833333
ARFGAP2	0.144	0.17	0.102	0.102	0	0.106	0.357	0	0	0	0.144	0.184	0.105	0.175	0.208	0.363	12	0.18	0.157	1568.5
BEST4	0.143	0.159	0.102	0.101	0	0.362	0.228	0.218	0.22	0	0	0.276	0.117	0.159	0	0.326	12	0.200916667	0.1885	1512.833333
ARL1	0.142	0.17	0.127	0.127	0	0.121	0.445	0	0	0	0.247	0.179	0.12	0.174	0.186	0.437	12	0.20625	0.172	1109.666667
RALGDS	0.141	0.157	0.113	0.113	0	0	0.195	0.139	0.14	0.141	0	0.116	0.124	0.163	0	0.223	12	0.147083333	0.1405	3007.333333
MTMR14	0.137	0.166	0.112	0.112	0	0.348	0.369	0	0	0	0.231	0.471	0.136	0.172	0.13	0.38	12	0.230333333	0.169	740.75
TBC1D8	0.136	0.132	0.117	0.117	0	0	0.229	0.136	0.138	0.149	0	0.13	0.114	0.134	0	0.232	12	0.147	0.135	2719.583333
MIR342	0.135	0.122	0.114	0.114	0	0	0.16	0.146	0.148	0.157	0	0.122	0.116	0.124	0	0.245	12	0.141916667	0.1295	3076
SPATC1	0.131	0.129	0.115	0.115	0	0	0.308	0.139	0.141	0.135	0	0.19	0.141	0.135	0	0.342	12	0.168416667	0.137	1424.166667
LOC440683	0.131	0.187	0.127	0.127	0.115	0.164	0.448	0	0	0	0.126	0.215	0.118	0.191	0	0.532	12	0.20675	0.1475	736.5833333
WDR44	0.131	0.165	0.103	0.103	0	0.149	0.412	0	0	0	0.139	0.17	0.117	0.17	0.21	0.414	12	0.19025	0.157	1192.833333
PYGB	0.13	0.144	0.139	0.139	0	0	0.32	0.116	0.122	0.103	0	0.121	0.121	0.147	0	0.313	12	0.159583333	0.1345	1979.333333
ILKAP	0.129	0.143	0.133	0.132	0	0	0.405	0	0.103	0.109	0	0.235	0.101	0.155	0.311	0.374	12	0.194166667	0.138	813.75
EPHA10	0.129	0.103	0	0	0	0.139	0.167	0.15	0.152	0.161	0	0.137	0.133	0.107	0.152	0.222	12	0.146	0.1445	3209.416667
DNM1	0.129	0.148	0	0	0	0.114	0.375	0.101	0.103	0.111	0	0.152	0.133	0.153	0.12	0.357	12	0.166333333	0.131	1686.833333
SGSM2	0.129	0.157	0.112	0.112	0	0.1	0.362	0	0	0	0.177	0.157	0.126	0.163	0.136	0.342	12	0.17275	0.1465	1858.416667
APPL2	0.122	0.148	0.122	0.122	0	0.13	0.468	0	0	0	0.107	0.283	0.103	0.156	0.117	0.461	12	0.194916667	0.126	795.25
DUSP16	0.12	0.115	0.103	0.103	0	0	0.311	0.146	0.147	0.156	0	0.188	0.102	0.129	0	0.228	12	0.154	0.1375	2004.5
AP1G2	0.119	0.123	0.102	0.102	0	0.157	0.407	0	0	0.107	0	0.178	0.109	0.128	0.143	0.394	12	0.172416667	0.1255	1253.416667
ITCH	0.115	0.126	0.119	0.119	0	0	0.315	0.103	0.105	0.133	0	0.233	0.106	0.131	0	0.323	12	0.160666667	0.1225	1332.666667
MAP3K5	0.114	0.113	0.107	0.107	0	0	0.36	0.126	0.127	0.128	0	0.25	0.101	0.127	0	0.284	12	0.162	0.1265	1321.5

RAB26	0.114	0.178	0.123	0.123	0	0.118	0.399	0	0	0	0.158	0.171	0.105	0.182	0.178	0.407	12	0.188	0.1645	1344.333333
POTEF	0.101	0.105	0.105	0.104	0	0	0.325	0.102	0.106	0.107	0	0.146	0	0.108	0.149	0.403	12	0.155083333	0.1065	1717.333333
ARR3	0.754	0.645	0.674	0.674	0.113	0	0	0.962	0.963	0.91	0	0	0.758	0.645	0	0.5	11	0.690727273	0.674	57
TMEM66	0.642	0.519	0.573	0.573	0	0	0.165	0.808	0.809	0.825	0	0	0.629	0.518	0	0.448	11	0.591727273	0.573	1274.909091
SLC9A5	0.537	0.452	0.51	0.51	0	0	0.12	0.676	0.676	0.718	0	0	0.551	0.452	0	0.396	11	0.508909091	0.51	1566.090909
GNAI1	0.448	0.578	0.457	0.457	0	0	0.176	0	0	0	0.931	0.123	0.42	0.579	0.142	0.497	11	0.437090909	0.457	2168.363636
UGT3A2	0.433	0.345	0.397	0.397	0	0	0.195	0.552	0.553	0.586	0	0	0.429	0.346	0	0.39	11	0.420272727	0.397	1225.090909
RIC8A	0.431	0.523	0.436	0.436	0	0	0.241	0	0	0	0.822	0.165	0.428	0.525	0.175	0.495	11	0.425181818	0.436	1444.909091
RGS2	0.429	0.556	0.446	0.446	0	0	0.274	0	0	0	0.764	0.167	0.397	0.559	0.113	0.557	11	0.428	0.446	1282.727273
GNA14	0.413	0.555	0.414	0.414	0	0	0.198	0	0	0	0.849	0.121	0.379	0.558	0.142	0.484	11	0.411545455	0.414	2084.090909
GNAO1	0.412	0.549	0.453	0.453	0	0	0.172	0	0	0	0.925	0.106	0.406	0.55	0.134	0.496	11	0.423272727	0.453	2360.181818
GNA15	0.412	0.551	0.448	0.448	0	0	0.163	0	0	0	0.94	0.116	0.426	0.552	0.142	0.461	11	0.423545455	0.448	2317
GNAZ	0.388	0.53	0.406	0.406	0	0	0.251	0	0	0	0.876	0.154	0.37	0.532	0.132	0.514	11	0.414454545	0.406	1504.636364
GGACT	0.385	0.328	0.379	0.379	0	0	0.126	0.486	0.486	0.518	0	0	0.366	0.329	0	0.299	11	0.371	0.379	1882.363636
LPAR3	0.382	0.505	0.351	0.351	0	0	0.3	0	0	0	0.742	0.205	0.356	0.51	0.166	0.5	11	0.397090909	0.356	891.3636364
GNAI3	0.379	0.509	0.408	0.408	0	0	0.145	0	0	0	0.923	0.103	0.345	0.509	0.132	0.438	11	0.390818182	0.408	2576.909091
GNB4	0.375	0.494	0.398	0.398	0	0	0.213	0	0	0	0.77	0.135	0.361	0.495	0.188	0.522	11	0.395363636	0.398	1860.818182
PLCB3	0.374	0.494	0.348	0.348	0	0	0.273	0	0	0	0.667	0.165	0.352	0.499	0.165	0.461	11	0.376909091	0.352	1307.363636
GUCA1B	0.368	0.316	0.251	0.251	0	0	0.159	0.624	0.625	0.364	0	0	0.356	0.317	0	0.352	11	0.362090909	0.352	1548
ZNF513	0.367	0.355	0.285	0.285	0	0	0	0.51	0.51	0.337	0.124	0	0.388	0.355	0	0.326	11	0.349272727	0.355	417.2727273
GNA13	0.367	0.517	0.374	0.374	0	0	0.174	0	0	0	0.916	0.129	0.395	0.52	0.168	0.413	11	0.395181818	0.374	2188.363636
PLCB2	0.363	0.487	0.324	0.324	0	0	0.246	0	0	0	0.71	0.148	0.391	0.491	0.221	0.443	11	0.377090909	0.363	1595.454545
RIC8B	0.362	0.457	0.425	0.425	0	0	0.203	0	0	0	0.821	0.144	0.341	0.457	0.152	0.449	11	0.385090909	0.425	1875.363636
GNA12	0.357	0.491	0.376	0.376	0	0	0.186	0	0	0	0.88	0.139	0.366	0.493	0.155	0.426	11	0.385909091	0.376	2028.181818
RGS5	0.345	0.439	0.345	0.345	0	0	0.244	0	0	0	0.639	0.166	0.305	0.441	0.147	0.472	11	0.353454545	0.345	1468.545455
PLCB1	0.329	0.442	0.35	0.35	0	0	0.261	0	0	0	0.678	0.15	0.318	0.446	0.166	0.428	11	0.356181818	0.35	1534.545455
RGSL1	0.321	0.42	0.289	0.289	0	0.116	0.245	0	0	0	0.637	0.181	0.286	0.422	0	0.489	11	0.335909091	0.289	1639.545455
RGS18	0.318	0.427	0.333	0.333	0	0.103	0.306	0	0	0	0.662	0.257	0.33	0.433	0	0.504	11	0.364181818	0.333	1069.272727
GUCA1A	0.315	0.275	0.21	0.21	0	0	0.184	0.56	0.561	0.299	0	0	0.298	0.276	0	0.342	11	0.320909091	0.298	1480.454545
GRK1	0.314	0.276	0.172	0.172	0	0	0.119	0.534	0.535	0.249	0	0	0.317	0.277	0	0.265	11	0.293636364	0.276	2162.181818

RGS13	0.313	0.351	0.241	0.241	0	0	0.214	0	0	0	0.444	0.153	0.246	0.354	0.145	0.34	11	0.276545455	0.246	2007.909091
GAGE2A	0.308	0.421	0.331	0.331	0	0	0.141	0	0	0	0.86	0.134	0.346	0.421	0.132	0.398	11	0.347545455	0.331	2369.363636
NDUFAF5	0.307	0.425	0.35	0.35	0	0	0.18	0	0	0	0.85	0.126	0.335	0.425	0.116	0.418	11	0.352909091	0.35	2226
GNB2	0.303	0.402	0.312	0.312	0	0	0.275	0	0	0	0.587	0.155	0.283	0.404	0.13	0.517	11	0.334545455	0.312	1408.545455
RGS14	0.301	0.423	0.315	0.315	0	0	0.303	0	0	0	0.715	0.16	0.313	0.427	0.123	0.469	11	0.351272727	0.315	1238.636364
FFAR2	0.288	0.349	0.165	0.165	0	0	0.282	0	0	0	0.28	0.163	0.189	0.352	0.14	0.357	11	0.248181818	0.28	1544.454545
GAGE2C	0.287	0.395	0.317	0.317	0	0	0.173	0	0	0	0.783	0.142	0.313	0.395	0.114	0.407	11	0.331181818	0.317	2144
RGS20	0.281	0.402	0.312	0.312	0	0	0.202	0	0	0	0.652	0.132	0.274	0.403	0.11	0.415	11	0.317727273	0.312	2082.636364
UGT3A1	0.28	0.226	0.25	0.25	0	0	0.203	0.353	0.355	0.374	0	0	0.275	0.226	0	0.32	11	0.282909091	0.275	1469.090909
FFAR3	0.28	0.365	0.228	0.228	0	0.105	0.31	0	0	0	0.405	0.192	0.226	0.371	0	0.425	11	0.285	0.28	1401.454545
ADCY10	0.275	0.383	0.267	0.267	0	0.351	0.314	0	0	0	0.453	0.367	0.251	0.387	0	0.472	11	0.344272727	0.351	579.5454545
RGS22	0.275	0.38	0.295	0.295	0	0	0.14	0	0	0	0.755	0.117	0.305	0.38	0.102	0.327	11	0.306454545	0.295	2779.363636
RGS19	0.273	0.368	0.284	0.284	0	0	0.323	0	0	0	0.665	0.168	0.274	0.375	0.108	0.426	11	0.322545455	0.284	1157.909091
KANK2	0.272	0.387	0.294	0.294	0	0	0.218	0	0	0	0.764	0.156	0.316	0.39	0.134	0.419	11	0.331272727	0.294	1756.909091
GPR20	0.269	0.355	0.26	0.259	0	0.193	0.232	0	0	0	0.239	0.196	0.183	0.357	0	0.438	11	0.271	0.259	1406.363636
S1PR4	0.264	0.342	0.208	0.208	0	0	0.206	0	0	0	0.374	0.131	0.21	0.345	0.288	0.375	11	0.268272727	0.264	2120.545455
FRMPD1	0.262	0.336	0.295	0.295	0	0	0.408	0	0	0	0.525	0.204	0.222	0.341	0.122	0.552	11	0.323818182	0.295	600.2727273
CSTF2T	0.262	0.371	0.289	0.289	0	0	0.297	0	0	0	0.705	0.177	0.292	0.372	0.113	0.482	11	0.331727273	0.292	1162.545455
TULP3	0.259	0.323	0.207	0.207	0	0	0.341	0	0	0	0.49	0.176	0.266	0.327	0.102	0.433	11	0.284636364	0.266	1061.636364
DGKZ	0.255	0.337	0.206	0.206	0	0	0.503	0	0	0	0.446	0.281	0.238	0.349	0.223	0.507	11	0.322818182	0.281	206.4545455
FPR3	0.25	0.273	0.19	0.19	0	0	0.183	0.164	0.169	0.176	0	0	0.186	0.275	0	0.317	11	0.215727273	0.19	1656
ARHGEF1	0.249	0.326	0.197	0.197	0	0	0.249	0	0	0	0.507	0.169	0.259	0.331	0.137	0.37	11	0.271909091	0.249	1623.363636
LPAR5	0.246	0.357	0.252	0.252	0	0.14	0.306	0	0	0	0.291	0.193	0.155	0.361	0	0.457	11	0.273636364	0.252	1176
RAP2B	0.238	0.302	0.187	0.187	0	0	0.313	0	0	0	0.403	0.163	0.173	0.306	0.152	0.425	11	0.259	0.238	1270.272727
GADL1	0.236	0.183	0.251	0.251	0	0	0.275	0.276	0.279	0.288	0	0	0.178	0.183	0	0.303	11	0.245727273	0.251	1219.636364
GNG5P2	0.236	0.304	0.233	0.233	0	0.111	0.291	0	0	0	0.421	0.192	0.215	0.308	0	0.492	11	0.276	0.236	1409.181818
ARHGEF25	0.234	0.317	0.235	0.235	0	0	0.158	0	0	0	0.609	0.151	0.243	0.319	0.243	0.302	11	0.276909091	0.243	2447.545455
GNAT1	0.232	0.215	0.119	0.119	0	0	0	0.445	0.445	0.142	0.107	0	0.211	0.214	0	0.218	11	0.224272727	0.214	1152.727273
PRKD1	0.232	0.281	0.157	0.157	0	0	0.475	0	0	0	0.371	0.232	0.251	0.297	0.129	0.379	11	0.269181818	0.251	557.6363636
ANAPC16	0.231	0.199	0.203	0.203	0	0	0.248	0.367	0.37	0.288	0	0	0.247	0.2	0	0.308	11	0.260363636	0.247	1318.636364

ADRA1A	0.23	0.292	0.205	0.205	0	0	0	0.151	0.157	0.143	0.124	0	0.294	0.292	0	0.247	11	0.212727273	0.205	902.9090909
PREX1	0.23	0.257	0.127	0.127	0	0	0.31	0	0	0	0.2	0.212	0.189	0.265	0.415	0.341	11	0.243	0.23	1187.909091
P2RY13	0.227	0.304	0.204	0.203	0	0.156	0.163	0	0	0	0.322	0.163	0.181	0.306	0	0.312	11	0.231	0.204	2418.090909
RASA3	0.227	0.327	0.231	0.231	0	0	0.208	0	0	0	0.603	0.127	0.208	0.332	0.134	0.315	11	0.267545455	0.231	2392.272727
GALR3	0.225	0.347	0.24	0.24	0	0.106	0.28	0	0	0	0.36	0.142	0.149	0.348	0	0.496	11	0.266636364	0.24	1904.727273
GALR2	0.223	0.32	0.218	0.218	0	0.111	0.239	0	0	0	0.244	0.129	0.138	0.322	0	0.451	11	0.237545455	0.223	2237.545455
NMUR1	0.217	0.297	0.201	0.201	0	0.133	0.283	0	0	0	0.24	0.181	0.131	0.3	0	0.431	11	0.237727273	0.217	1459.272727
OR13J1	0.215	0.217	0.168	0.168	0	0	0.108	0.172	0.175	0.176	0	0	0.193	0.218	0	0.285	11	0.190454545	0.176	2210.818182
MTNR1A	0.214	0.259	0.235	0.235	0.33	0	0.191	0	0	0	0.161	0.121	0.134	0.261	0	0.352	11	0.226636364	0.235	2411.181818
MIR138-2	0.214	0.288	0.225	0.225	0	0	0.222	0	0	0	0.557	0.187	0.182	0.289	0.108	0.392	11	0.262636364	0.225	1640.363636
GNG10	0.213	0.295	0.264	0.264	0	0.143	0.42	0	0	0	0.346	0.207	0.134	0.299	0	0.506	11	0.281	0.264	713.3636364
DGKB	0.209	0.254	0.151	0.151	0	0	0.386	0	0	0	0.187	0.18	0.148	0.262	0.126	0.458	11	0.228363636	0.187	950.7272727
ADRA1D	0.208	0.293	0.207	0.207	0	0	0	0.115	0.121	0.109	0.161	0	0.281	0.294	0	0.262	11	0.205272727	0.207	838.4545455
GPR42	0.208	0.266	0.184	0.184	0	0.137	0.288	0	0	0	0.298	0.177	0.178	0.268	0	0.382	11	0.233636364	0.208	1517.909091
P2RY10	0.206	0.27	0.222	0.222	0	0	0.156	0	0.101	0.105	0.185	0	0.175	0.271	0	0.286	11	0.199909091	0.206	1975.636364
MCHR1	0.206	0.276	0.194	0.194	0	0.102	0.196	0	0	0	0.142	0.117	0.101	0.277	0	0.369	11	0.197636364	0.194	2848.818182
CSTF2	0.204	0.283	0.213	0.213	0	0	0.446	0	0	0	0.549	0.201	0.229	0.286	0.111	0.552	11	0.298818182	0.229	597
P2RY2	0.202	0.266	0.146	0.146	0	0.166	0.183	0	0	0	0.191	0.148	0.138	0.269	0	0.288	11	0.194818182	0.183	2608.545455
ADCY3	0.202	0.278	0.181	0.181	0	0.165	0.365	0	0	0	0.241	0.206	0.186	0.283	0	0.454	11	0.249272727	0.206	838.2727273
GPR183	0.196	0.223	0.129	0.129	0	0.144	0.296	0	0	0	0.145	0.204	0.137	0.227	0	0.347	11	0.197909091	0.196	1486.363636
MMADHC	0.195	0.255	0.198	0.198	0	0.109	0.359	0	0	0	0.472	0.171	0.212	0.257	0	0.414	11	0.258181818	0.212	1376.090909
PLCE1	0.195	0.224	0.132	0.132	0	0	0.262	0	0	0	0.197	0.159	0.131	0.232	0.109	0.307	11	0.189090909	0.195	2013.454545
GNAT2	0.194	0.218	0.117	0.117	0	0	0.166	0.302	0.304	0	0.25	0	0.172	0.218	0	0.29	11	0.213454545	0.218	1937.727273
P2RY4	0.193	0.268	0.18	0.18	0	0.168	0.169	0	0	0	0.196	0.146	0.146	0.269	0	0.318	11	0.203	0.18	2525.727273
GLP2R	0.189	0.218	0.156	0.156	0	0	0	0.131	0.133	0.151	0	0.101	0.129	0.219	0	0.222	11	0.164090909	0.156	2233
ADCY4	0.189	0.256	0.262	0.262	0	0.13	0.233	0	0	0	0.3	0.18	0.151	0.258	0	0.381	11	0.236545455	0.256	1819.363636
IGHV1-3	0.189	0.279	0.228	0.228	0	0.104	0.176	0	0	0	0.479	0	0.207	0.28	0.101	0.334	11	0.236818182	0.228	2083.272727
PPAN-P2RY11	0.188	0.258	0.175	0.175	0	0.159	0.319	0	0	0	0.231	0.191	0.127	0.261	0	0.431	11	0.228636364	0.191	1170.818182
FFAR4	0.182	0.195	0.155	0.155	0	0	0.179	0.14	0.142	0.149	0	0	0.15	0.196	0	0.244	11	0.171545455	0.155	2133.909091
PRLHR	0.182	0.237	0.18	0.18	0.237	0.127	0.283	0	0	0	0	0.171	0.101	0.24	0	0.406	11	0.213090909	0.182	1643.636364

GPR156	0.182	0.213	0.195	0.195	0	0.213	0.373	0	0	0	0.115	0.271	0.135	0.218	0	0.473	11	0.234818182	0.213	606.0909091
ADAMTS18	0.181	0.209	0.165	0.165	0	0.101	0.275	0	0	0	0.343	0.131	0.159	0.209	0	0.345	11	0.207545455	0.181	2383.272727
BAI2	0.181	0.215	0.153	0.153	0	0.159	0.453	0	0	0	0.152	0.22	0.128	0.219	0	0.481	11	0.228545455	0.181	663.4545455
GNG12	0.181	0.208	0.164	0.164	0	0.122	0.464	0	0	0	0.213	0.188	0.101	0.216	0	0.444	11	0.224090909	0.188	1013.818182
P2RY6	0.181	0.261	0.155	0.155	0	0.174	0.214	0	0	0	0.239	0.173	0.145	0.264	0	0.316	11	0.207	0.181	2071.545455
PIP5K1A	0.18	0.204	0.148	0.148	0	0	0.331	0	0	0	0.175	0.192	0.171	0.212	0.345	0.345	11	0.222818182	0.192	1222.181818
PDE1B	0.177	0.231	0.115	0.114	0	0.144	0.349	0	0	0	0.118	0.183	0.13	0.236	0	0.437	11	0.203090909	0.177	1267.818182
RNU6V	0.177	0.233	0.214	0.214	0	0.2	0.434	0	0	0	0.319	0.319	0.138	0.237	0	0.572	11	0.277909091	0.233	339.3636364
RAP1GAP	0.177	0.244	0.131	0.131	0	0	0.376	0	0	0	0.28	0.237	0.116	0.252	0.248	0.425	11	0.237909091	0.244	705.1818182
LDLRAP1	0.176	0.156	0.199	0.199	0	0	0.144	0.213	0.214	0.219	0	0	0.162	0.157	0	0.199	11	0.185272727	0.199	2561.363636
GPR35	0.176	0.195	0.146	0.146	0	0.132	0.259	0	0	0	0.152	0.147	0.138	0.198	0	0.301	11	0.180909091	0.152	2321.363636
EPN2	0.174	0.168	0	0	0	0.122	0.407	0.113	0.118	0.131	0	0.185	0.146	0.172	0	0.425	11	0.196454545	0.168	1171.363636
GUCY1A2	0.174	0.247	0.162	0.162	0	0.103	0.279	0	0	0	0.287	0.15	0.126	0.25	0	0.365	11	0.209545455	0.174	2100.818182
GPR68	0.172	0.22	0.158	0.158	0	0.107	0.334	0	0	0	0.218	0.195	0.13	0.225	0	0.337	11	0.204909091	0.195	1600.818182
OR2B2	0.171	0.19	0.191	0.191	0	0.14	0.387	0	0	0	0.173	0.195	0.101	0.195	0	0.489	11	0.220272727	0.191	1001.545455
LPHN3	0.169	0.203	0.154	0.153	0	0.156	0.35	0	0	0	0.176	0.183	0.162	0.207	0	0.396	11	0.209909091	0.176	1202.545455
PIP5K1B	0.169	0.205	0.12	0.12	0	0	0.303	0	0	0	0.223	0.186	0.153	0.213	0.225	0.331	11	0.204363636	0.205	1473.727273
SGPP1	0.167	0.197	0.1	0.1	0	0.168	0.329	0	0	0	0.247	0.236	0.142	0.201	0	0.314	11	0.200090909	0.197	1300.454545
GNG4	0.166	0.206	0.145	0.145	0	0	0.282	0	0	0	0.242	0.151	0.143	0.211	0.129	0.33	11	0.195454545	0.166	1863.454545
GPR22	0.164	0.195	0.18	0.18	0.147	0	0.344	0	0	0	0.116	0.164	0.138	0.198	0	0.362	11	0.198909091	0.18	1362.454545
C14ORF23	0.163	0.135	0.13	0.13	0	0	0.168	0.194	0.194	0.184	0	0	0.118	0.136	0	0.218	11	0.160909091	0.163	2450
CUEDC1	0.163	0.135	0.13	0.13	0	0	0.168	0.194	0.194	0.184	0	0	0.118	0.136	0	0.218	11	0.160909091	0.163	2451
C4ORF19	0.163	0.135	0.13	0.13	0	0	0.168	0.194	0.194	0.184	0	0	0.118	0.136	0	0.218	11	0.160909091	0.163	2452
PLCH1	0.159	0.182	0.133	0.133	0	0	0.122	0.107	0.109	0.12	0	0	0.112	0.184	0	0.197	11	0.141636364	0.133	2789.272727
ADCY7	0.157	0.216	0.154	0.154	0	0.163	0.382	0	0	0	0.219	0.249	0.15	0.225	0	0.351	11	0.22	0.216	873.6363636
PKD1L2	0.156	0.18	0.103	0.103	0.104	0.103	0.201	0	0	0	0	0.125	0.122	0.181	0	0.307	11	0.153181818	0.125	3111.909091
FCHO1	0.156	0.208	0.151	0.151	0	0	0.336	0	0	0	0.225	0.177	0.213	0.21	0.185	0.401	11	0.219363636	0.208	1178.727273
INTU	0.152	0.199	0.128	0.128	0	0.116	0.317	0	0	0	0.296	0.244	0.166	0.201	0	0.408	11	0.214090909	0.199	1228
PIGG	0.15	0.193	0.117	0.117	0.158	0.125	0.403	0	0	0	0.122	0.177	0	0.198	0	0.372	11	0.193818182	0.158	1343.545455
PDE3A	0.15	0.212	0.114	0.114	0	0.25	0.403	0	0	0	0.119	0.335	0.161	0.221	0	0.364	11	0.222090909	0.212	692.3636364

KCNJ14	0.15	0.235	0.179	0.178	0	0.225	0.308	0	0	0	0.271	0.177	0.107	0.237	0	0.43	11	0.227	0.225	1255.090909
MCOLN2	0.149	0.216	0.108	0.108	0	0.157	0.304	0	0	0	0.164	0.147	0.181	0.219	0	0.411	11	0.196727273	0.164	1698.545455
GPR65	0.149	0.185	0.119	0.119	0	0.114	0.34	0	0	0	0.124	0.192	0.102	0.191	0	0.343	11	0.179818182	0.149	1647.636364
PEX11G	0.149	0.202	0.15	0.15	0	0.141	0.365	0	0	0	0.231	0.193	0.142	0.205	0	0.432	11	0.214545455	0.193	1097.363636
ZMYND19	0.149	0.208	0.201	0.201	0	0.155	0.402	0	0	0	0.14	0.234	0.102	0.213	0	0.493	11	0.227090909	0.201	728.0909091
PPP1R9A	0.148	0.169	0.12	0.12	0	0	0.324	0	0	0	0.153	0.133	0.153	0.175	0.146	0.401	11	0.185636364	0.153	1714.545455
GRM6	0.145	0.165	0.112	0.112	0	0	0.129	0.264	0.264	0	0.116	0	0.115	0.165	0	0.263	11	0.168181818	0.145	2412
OR10J5	0.144	0.182	0.135	0.135	0	0.152	0.229	0	0	0	0.139	0.111	0.136	0.183	0	0.383	11	0.175363636	0.144	2486.636364
ACSM1	0.141	0.188	0.148	0.148	0	0.109	0.34	0	0	0	0.307	0.154	0.131	0.191	0	0.381	11	0.203454545	0.154	1766.545455
PDE1C	0.138	0.181	0.107	0.107	0	0.129	0.322	0	0	0	0.122	0.208	0.114	0.186	0	0.38	11	0.181272727	0.138	1449
ZDHHC7	0.138	0.171	0.167	0.167	0	0	0.2	0	0.102	0	0.134	0.118	0.157	0.172	0	0.297	11	0.165727273	0.167	2760.454545
ARF4	0.137	0.158	0.132	0.132	0	0.15	0.481	0	0	0	0.139	0.221	0	0.164	0.16	0.442	11	0.210545455	0.158	779.2727273
KIAA0368	0.136	0.178	0.114	0.114	0	0.185	0.597	0	0	0	0.191	0.292	0.11	0.184	0	0.538	11	0.239909091	0.184	442.5454545
VAC14	0.135	0.159	0.113	0.113	0	0.14	0.499	0	0	0	0.172	0.313	0.104	0.166	0	0.447	11	0.214636364	0.159	646.9090909
ASAP2	0.133	0.15	0.127	0.127	0	0	0.347	0	0	0	0.104	0.173	0.129	0.16	0.536	0.36	11	0.213272727	0.15	1398
COPA	0.131	0.16	0.137	0.137	0	0.235	0.476	0	0	0	0	0.255	0.102	0.166	0.162	0.466	11	0.220636364	0.162	544.8181818
PAIP1	0.131	0.124	0	0	0	0	0.56	0.114	0.12	0.128	0	0.217	0.141	0.13	0.109	0.475	11	0.204454545	0.13	697.1818182
SEC24A	0.13	0.124	0	0	0	0.337	0.47	0.127	0.131	0.128	0	0.39	0.111	0.128	0	0.422	11	0.227090909	0.13	502.7272727
MYO16	0.13	0.164	0.104	0.104	0	0.161	0.506	0	0	0	0.104	0.267	0.112	0.169	0	0.467	11	0.208	0.161	658.3636364
FCHO2	0.129	0.172	0.131	0.131	0	0	0.347	0	0	0	0.214	0.2	0.172	0.175	0.183	0.361	11	0.201363636	0.175	1149.818182
PDE7A	0.126	0.196	0.13	0.13	0	0.119	0.342	0	0	0	0.116	0.177	0.107	0.201	0	0.364	11	0.182545455	0.13	1630
NOL12	0.126	0.149	0.118	0.118	0	0	0.429	0	0	0	0.124	0.18	0.12	0.154	0.168	0.432	11	0.192545455	0.149	1041.909091
AP4M1	0.124	0.128	0	0	0	0.175	0.476	0.133	0.137	0.131	0	0.203	0.12	0.132	0	0.461	11	0.201818182	0.133	799.5454545
LOC647323	0.123	0.181	0.133	0.133	0	0	0.257	0	0	0	0.307	0.178	0.126	0.183	0.139	0.312	11	0.188363636	0.178	1899.272727
RFX8	0.123	0.181	0.133	0.133	0	0	0.257	0	0	0	0.307	0.178	0.126	0.183	0.139	0.312	11	0.188363636	0.178	1898.272727
SEC24D	0.121	0.177	0.117	0.117	0	0.24	0.342	0	0	0	0.106	0.259	0.109	0.18	0	0.382	11	0.195454545	0.177	976
SLC38A2	0.119	0.169	0.111	0.111	0	0.139	0.425	0	0	0	0.123	0.232	0.102	0.173	0	0.385	11	0.189909091	0.139	1001.727273
KALRN	0.118	0.159	0.102	0.102	0	0	0.277	0	0	0	0.244	0.147	0.108	0.164	0.296	0.33	11	0.186090909	0.159	2040.090909
PPP1CC	0.117	0.156	0.123	0.123	0	0	0.481	0	0	0	0.268	0.258	0.126	0.168	0.104	0.428	11	0.213818182	0.156	613.9090909
AASS	0.114	0.16	0.131	0.131	0.188	0	0.36	0	0	0	0.107	0.194	0.106	0.161	0	0.374	11	0.184181818	0.16	1206.636364

SLC9A9	0.114	0.147	0.101	0.101	0	0.178	0.233	0	0	0	0.143	0.16	0.1	0.149	0	0.285	11	0.155545455	0.147	2471.636364
RAB32	0.114	0.158	0.129	0.129	0	0.137	0.31	0	0	0	0.276	0.2	0.122	0.162	0	0.307	11	0.185818182	0.158	1710.272727
GMPR	0.11	0.11	0.106	0.106	0	0	0.381	0.126	0.13	0	0	0.134	0.101	0.113	0	0.292	11	0.155363636	0.113	2065
KCNT2	0.109	0.184	0.132	0.132	0.117	0.228	0.174	0	0	0	0.19	0.168	0	0.185	0	0.318	11	0.176090909	0.174	2383.545455
AKAP3	0.106	0.158	0.1	0.1	0	0.105	0.4	0	0	0	0.194	0.167	0.169	0.166	0	0.341	11	0.182363636	0.166	1760.363636
ATP9A	0.105	0.138	0.115	0.115	0	0.375	0.337	0	0	0	0.269	0.417	0.107	0.139	0	0.364	11	0.225545455	0.139	976.5454545
SGSM1	0.101	0.135	0.116	0.116	0	0	0.346	0	0	0	0.133	0.181	0.111	0.139	0.145	0.356	11	0.170818182	0.135	1479.545455
GBA3	0	0.105	0.105	0.105	0	0.171	0.311	0.108	0.109	0.113	0	0.108	0	0.106	0	0.275	11	0.146909091	0.108	2666
ARRB2	0.778	0.636	0.709	0.709	0	0	0	0.937	0.937	0.995	0	0	0.756	0.635	0	0.455	10	0.7547	0.7325	35.5
ARRB1	0.774	0.633	0.697	0.697	0	0	0	0.961	0.961	0.988	0	0	0.748	0.633	0	0.455	10	0.7547	0.7225	35.7
OR7C2	0.655	0.497	0.485	0.486	0	0	0	0.709	0.714	0.769	0	0	0.624	0.495	0	0.352	10	0.5786	0.5605	244.2
SAG	0.518	0.426	0.377	0.377	0	0	0	0.794	0.794	0.561	0	0	0.502	0.426	0	0.352	10	0.5127	0.464	259.7
ARRDC3	0.407	0.322	0.365	0.365	0	0	0	0.49	0.492	0.522	0	0	0.398	0.322	0	0.295	10	0.3978	0.3815	529.5
GNAI2	0.384	0.516	0.421	0.421	0	0	0.141	0	0	0	0.892	0	0.341	0.516	0.118	0.45	10	0.42	0.421	1633.6
KLHL12	0.359	0.315	0.335	0.335	0	0	0	0.45	0.45	0.487	0	0	0.347	0.315	0	0.263	10	0.3656	0.341	716.8
TNFAIP8	0.307	0.424	0.344	0.344	0	0	0.205	0	0	0	0.82	0.158	0.284	0.425	0	0.433	10	0.3744	0.344	1898.5
MSANTD3	0.293	0.408	0.311	0.311	0	0	0.143	0	0	0	0.642	0.102	0.263	0.41	0	0.372	10	0.3255	0.311	2930.2
ADCY5	0.273	0.348	0.29	0.29	0	0	0.24	0	0	0	0.437	0.128	0.243	0.351	0	0.4	10	0.3	0.29	2061.2
LPAR4	0.266	0.339	0.236	0.236	0	0	0.278	0	0	0	0.334	0.189	0.216	0.343	0	0.419	10	0.2856	0.272	1288
RGS10	0.261	0.333	0.249	0.249	0	0	0.323	0	0	0	0.484	0.161	0.242	0.34	0	0.415	10	0.3057	0.292	1275.4
ADCY6	0.251	0.346	0.281	0.28	0	0	0.289	0	0	0	0.415	0.159	0.222	0.352	0	0.43	10	0.3025	0.285	1442.4
FFAR1	0.244	0.312	0.218	0.217	0	0	0.342	0	0	0	0.296	0.21	0.166	0.319	0	0.403	10	0.2727	0.27	897.3
HCAR3	0.23	0.318	0.193	0.193	0	0	0.219	0	0	0	0.163	0.143	0.172	0.32	0	0.395	10	0.2346	0.206	2138.2
GNAQP1	0.227	0.32	0.22	0.22	0	0	0.289	0	0	0	0.509	0.128	0.242	0.322	0	0.447	10	0.2924	0.2655	1752.5
GPR151	0.218	0.236	0.184	0.184	0	0	0	0.155	0.157	0.16	0	0	0.14	0.235	0	0.283	10	0.1952	0.184	790.9
GPR141	0.212	0.273	0.258	0.258	0.206	0	0	0.132	0.133	0.112	0	0	0	0.272	0	0.291	10	0.2147	0.235	699.2
GPR135	0.212	0.273	0.258	0.258	0.206	0	0	0.132	0.133	0.112	0	0	0	0.272	0	0.291	10	0.2147	0.235	700.2
PTH1R	0.211	0.217	0.285	0.285	0	0	0	0.143	0.143	0.168	0	0	0.107	0.217	0	0.186	10	0.1962	0.1985	1392.7
NKPD1	0.207	0.278	0.194	0.194	0	0	0.177	0	0	0	0.351	0	0.173	0.28	0.11	0.293	10	0.2257	0.2005	2021.7
TTC1	0.206	0.264	0.269	0.269	0	0	0.277	0	0	0	0.516	0.13	0.18	0.266	0	0.377	10	0.2754	0.2675	1921.6

HRH3	0.204	0.285	0.164	0.164	0	0.112	0.186	0	0	0	0.124	0	0.137	0.287	0	0.346	10	0.2009	0.175	2111.6
PITPNA	0.199	0.278	0.2	0.2	0	0	0.346	0	0	0	0.456	0.179	0.221	0.284	0	0.359	10	0.2722	0.2495	1203.3
RGS6	0.198	0.26	0.18	0.18	0	0	0.225	0	0	0	0.389	0.131	0.176	0.261	0	0.397	10	0.2397	0.2115	2218.6
ARHGEF12	0.198	0.259	0.172	0.172	0	0	0.21	0	0	0	0.471	0.148	0.175	0.265	0	0.318	10	0.2388	0.204	2394.8
NUCB1	0.197	0.261	0.216	0.216	0	0	0.366	0	0	0	0.454	0.156	0.168	0.263	0	0.415	10	0.2712	0.2385	1212.9
LHCGR	0.189	0.239	0.308	0.307	0.735	0.123	0.122	0	0	0.101	0	0	0	0.24	0	0.296	10	0.266	0.2395	2491.1
GALR1	0.189	0.272	0.176	0.176	0	0	0.207	0	0	0	0.141	0.107	0.109	0.273	0	0.39	10	0.204	0.1825	2660.7
GNGT1	0.186	0.165	0.224	0.224	0	0	0.127	0.182	0.183	0	0.188	0	0	0.166	0	0.249	10	0.1894	0.1845	2552.1
GNG8	0.186	0.256	0.198	0.198	0	0	0.322	0	0	0	0.328	0.196	0.169	0.26	0	0.485	10	0.2598	0.227	1035.5
TSNAX	0.186	0.251	0.184	0.184	0	0	0.35	0	0	0	0.343	0.176	0.151	0.254	0	0.453	10	0.2532	0.2185	1097
C1ORF27	0.186	0.237	0.315	0.315	0	0	0.193	0	0	0	0.333	0.14	0.146	0.238	0	0.35	10	0.2453	0.2375	2422.3
GPR17	0.186	0.228	0.128	0.128	0	0	0.3	0	0	0	0.188	0.176	0.141	0.23	0	0.365	10	0.207	0.187	1548.6
RBM27	0.185	0.161	0.162	0.162	0	0	0	0.224	0.224	0.211	0	0	0.19	0.161	0	0.21	10	0.189	0.1875	1276
RLN3	0.184	0.251	0.181	0.18	0.249	0.135	0.275	0	0	0	0	0.112	0	0.253	0	0.429	10	0.2249	0.2165	2279.1
RIEG2	0.184	0.226	0.188	0.188	0	0	0.202	0	0	0	0.325	0.135	0.178	0.228	0	0.298	10	0.2152	0.195	2672.4
ADCY2	0.18	0.204	0.248	0.248	0	0	0.189	0	0	0	0.266	0.11	0.109	0.206	0	0.301	10	0.2061	0.205	3019.2
GPSM2	0.178	0.227	0.209	0.209	0	0	0.325	0	0	0	0.412	0.166	0.134	0.231	0	0.415	10	0.2506	0.218	1316.1
QRFPR	0.175	0.256	0.154	0.154	0	0.105	0.277	0	0	0	0	0.144	0.108	0.257	0	0.41	10	0.204	0.1645	2272.2
HCAR1	0.172	0.234	0.203	0.203	0	0	0.318	0	0	0	0.21	0.242	0.132	0.238	0	0.378	10	0.233	0.222	990.2
GNG7	0.171	0.235	0.129	0.129	0	0	0.251	0	0	0	0.209	0.149	0.134	0.239	0	0.352	10	0.1998	0.19	2104.2
GPRC6A	0.169	0.237	0.19	0.19	0	0.106	0.282	0	0	0	0.11	0.226	0	0.241	0	0.375	10	0.2126	0.208	1697.1
GPR87	0.169	0.2	0.143	0.143	0	0	0.296	0	0	0	0.18	0.199	0.145	0.202	0	0.325	10	0.2002	0.1895	1544.9
PCP2	0.165	0.196	0.177	0.177	0	0	0.225	0	0	0	0.313	0.128	0.132	0.199	0	0.295	10	0.2007	0.1865	2669.8
KLHL3	0.164	0.24	0.168	0.168	0	0	0.257	0	0	0	0.315	0.181	0.159	0.24	0	0.339	10	0.2231	0.2105	1783.7
PTPRU	0.16	0.2	0.191	0.191	0	0	0.269	0	0	0	0.355	0.138	0.118	0.203	0	0.343	10	0.2168	0.1955	2100.7
TAAR2	0.158	0.192	0.166	0.166	0.134	0	0.177	0	0.101	0	0	0.155	0	0.192	0	0.305	10	0.1746	0.166	2686.6
LCS1	0.157	0.139	0.12	0.119	0	0	0	0.125	0.126	0.133	0	0	0.101	0.14	0	0.18	10	0.134	0.1295	1675.2
RGS12	0.156	0.227	0.202	0.202	0	0	0.251	0	0	0	0.29	0.185	0.104	0.23	0	0.36	10	0.2207	0.2145	1733.4
ADCY1	0.155	0.193	0.19	0.19	0	0.156	0.244	0	0	0	0.214	0.181	0	0.195	0	0.346	10	0.2064	0.1915	1966.9
RAB11FIP2	0.153	0.173	0	0	0	0.175	0.398	0	0	0	0.105	0.205	0.114	0.177	0.156	0.407	10	0.2063	0.174	1020.1

LTB4R	0.152	0.159	0.112	0.112	0	0	0.124	0	0.102	0.107	0	0	0.127	0.16	0	0.187	10	0.1342	0.1255	3152.4
PDE6H	0.152	0.173	0	0	0	0	0.2	0.285	0.286	0	0.155	0.102	0.123	0.175	0	0.284	10	0.1935	0.174	3169.2
SUCNR1	0.151	0.202	0.113	0.113	0	0	0.238	0	0	0	0.151	0.151	0.112	0.205	0	0.275	10	0.1711	0.151	2595.1
ARL6	0.151	0.172	0	0	0	0.182	0.441	0	0	0	0.134	0.299	0.101	0.175	0.125	0.481	10	0.2261	0.1735	570.3
RAB6A	0.145	0.148	0	0	0	0.169	0.453	0	0	0	0.125	0.211	0.118	0.152	0.132	0.43	10	0.2083	0.15	893.1
KIF3A	0.142	0.123	0	0	0	0.114	0.331	0.158	0.161	0	0	0.243	0.111	0.127	0	0.342	10	0.1852	0.15	1566.7
WDR27	0.142	0.184	0.128	0.128	0	0	0.443	0	0	0	0.267	0.214	0.153	0.189	0	0.432	10	0.228	0.1865	712
CNGA4	0.141	0.19	0	0	0	0.235	0.321	0.138	0.143	0	0.119	0.211	0	0.194	0	0.399	10	0.2091	0.192	1151.7
EMR4P	0.14	0.169	0.101	0.1	0.143	0.209	0.294	0	0	0	0	0.149	0	0.17	0	0.351	10	0.1826	0.159	1991
TSPAN11	0.14	0.168	0.118	0.118	0	0.205	0.49	0	0	0	0.118	0.237	0	0.173	0	0.455	10	0.2222	0.1705	634.2
COQ4	0.14	0.198	0.168	0.168	0	0	0.243	0	0	0	0.402	0.123	0.158	0.2	0	0.363	10	0.2163	0.183	2336.4
GPR64	0.139	0.18	0.162	0.162	0.239	0.179	0.25	0	0	0	0	0.149	0	0.182	0	0.373	10	0.2015	0.1795	2084.7
GPR180	0.138	0.148	0.143	0.143	0.185	0	0.134	0.109	0.111	0	0	0	0	0.149	0	0.152	10	0.1412	0.143	3277.9
MAMDC4	0.137	0.151	0.132	0.132	0	0.277	0.434	0	0	0	0	0.263	0.101	0.155	0	0.425	10	0.2207	0.153	635.5
ADCY8	0.136	0.199	0.111	0.111	0	0.119	0.338	0	0	0	0.152	0.185	0	0.204	0	0.393	10	0.1948	0.1685	1608.9
SLC9A3R1	0.135	0.154	0.119	0.119	0	0.296	0.147	0	0	0	0	0.266	0.114	0.155	0	0.189	10	0.1694	0.1505	3035.7
KDELR3	0.133	0.138	0	0	0	0.26	0.423	0.12	0.125	0	0	0.274	0.102	0.142	0	0.399	10	0.2116	0.14	681
QTRT1	0.132	0.183	0.158	0.158	0	0	0.29	0	0	0	0.342	0.105	0.146	0.186	0	0.308	10	0.2008	0.1705	2505.6
SLC13A5	0.131	0.18	0.129	0.129	0	0.129	0.401	0	0	0	0.194	0.184	0	0.184	0	0.397	10	0.2058	0.182	1304.4
CNGA2	0.131	0.201	0.122	0.122	0	0.214	0.275	0	0	0	0.123	0.184	0	0.204	0	0.391	10	0.1967	0.1925	1639.4
OR7E24	0.13	0.167	0.125	0.124	0	0.113	0.35	0	0	0	0	0.188	0.104	0.169	0	0.461	10	0.1931	0.1485	1548.4
GUCY2GP	0.128	0.194	0.155	0.155	0	0.206	0.382	0	0	0	0.159	0.229	0	0.199	0	0.438	10	0.2245	0.1965	787.8
TRNAA2	0.128	0.197	0.151	0.151	0	0	0.214	0	0	0	0.438	0.103	0.162	0.197	0	0.268	10	0.2009	0.1795	3168.8
TRNAA3	0.128	0.197	0.151	0.151	0	0	0.214	0	0	0	0.438	0.103	0.162	0.197	0	0.268	10	0.2009	0.1795	3167.8
SLC9A3R2	0.126	0.157	0.127	0.127	0	0.316	0.208	0	0	0	0	0.288	0.102	0.16	0	0.249	10	0.186	0.1585	2260
RBAK	0.126	0.181	0.139	0.139	0	0	0.278	0	0	0	0.337	0.171	0.118	0.183	0	0.373	10	0.2045	0.176	1723.7
ARHGEF39	0.125	0.181	0.131	0.131	0	0	0.108	0	0	0	0.385	0.116	0.138	0.182	0	0.196	10	0.1693	0.1345	4130.7
SLC6A16	0.123	0.168	0.133	0.132	0	0.286	0.564	0	0	0	0.119	0.318	0	0.174	0	0.573	10	0.259	0.171	394.1
GRASP	0.123	0.14	0.147	0.147	0	0.117	0.266	0	0	0	0	0.141	0	0.147	0.147	0.313	10	0.1688	0.147	2695.3
RALB	0.123	0.142	0	0	0	0	0.294	0	0	0.105	0.119	0.204	0.148	0.148	0.222	0.278	10	0.1783	0.148	1856.1

PROKR1	0.122	0.209	0.151	0.151	0	0.103	0.223	0	0	0	0.172	0.162	0	0.211	0	0.342	10	0.1846	0.167	2640.4
GIT2	0.122	0.137	0.105	0.105	0	0	0.18	0	0	0	0	0.116	0.109	0.145	0.798	0.227	10	0.2044	0.1295	3676.4
PEX26	0.12	0.131	0.109	0.109	0	0.177	0.484	0	0	0.104	0	0.306	0	0.135	0	0.453	10	0.2128	0.133	611
SAR1B	0.12	0.152	0.118	0.118	0	0.153	0.326	0	0	0	0.22	0.204	0	0.155	0	0.3	10	0.1866	0.154	1717.3
PI4K2A	0.12	0.154	0	0	0	0.11	0.433	0	0	0	0.121	0.158	0.122	0.159	0.113	0.35	10	0.184	0.138	1829.5
SPACA3	0.118	0.13	0.113	0.113	0.426	0.204	0.357	0	0	0	0	0.151	0	0.134	0	0.364	10	0.211	0.1425	1692.2
KCNJ4	0.118	0.158	0.109	0.109	0	0.196	0.277	0	0	0	0.134	0.141	0	0.162	0	0.338	10	0.1742	0.1495	2265.5
PRD	0.118	0.108	0	0	0	0	0.23	0.143	0.144	0.126	0	0.106	0.105	0.114	0	0.219	10	0.1413	0.122	3567.8
PQBP4	0.117	0.102	0	0	0	0	0.356	0.116	0.119	0.112	0	0.135	0.101	0.105	0	0.348	10	0.1611	0.1165	2023.6
VPS26B	0.117	0.143	0	0	0	0.15	0.514	0	0	0	0.12	0.248	0.105	0.149	0.101	0.507	10	0.2154	0.146	780.2
PALM	0.116	0.15	0	0	0	0.123	0.337	0.13	0.134	0	0.122	0.145	0	0.153	0	0.373	10	0.1783	0.1395	2009.2
CNGA1	0.115	0.15	0	0	0	0.183	0.259	0.231	0.234	0	0.105	0.16	0	0.153	0	0.332	10	0.1922	0.1715	2147.2
DACT3	0.114	0	0.103	0.103	0	0	0.222	0.148	0.152	0.153	0	0.221	0.123	0	0	0.242	10	0.1581	0.15	2381.4
CHST9	0.114	0.154	0.198	0.197	0.499	0.171	0.285	0	0	0	0	0.113	0	0.156	0	0.389	10	0.2276	0.184	2251.3
DLG2-AS1	0.114	0.122	0.103	0.103	0	0.112	0.296	0	0	0	0.108	0.142	0	0.124	0	0.375	10	0.1599	0.118	2479.9
MYPOP	0.114	0.114	0	0	0	0.103	0.279	0.213	0.216	0	0	0.117	0.126	0.116	0	0.288	10	0.1686	0.1215	3133.8
ARHGAP36	0.114	0.18	0.102	0.102	0	0	0.201	0	0	0	0.157	0.152	0	0.183	0.145	0.262	10	0.1598	0.1545	2947.3
GPR6	0.113	0.135	0.127	0.127	0.237	0.108	0.199	0	0	0	0.101	0	0	0.137	0	0.267	10	0.1551	0.131	2672.5
GGCT	0.112	0.113	0.11	0.11	0	0	0.186	0.105	0.109	0.102	0	0	0	0.114	0	0.252	10	0.1313	0.111	2541.2
MPP1	0.112	0.119	0.101	0.101	0	0.164	0.436	0	0	0	0.105	0.17	0	0.124	0	0.458	10	0.189	0.1215	1349.9
FLNB	0.112	0.111	0.114	0.114	0.177	0	0.183	0	0	0	0	0.11	0	0.113	0.109	0.231	10	0.1374	0.1135	3821.8
INPP4A	0.112	0.168	0	0	0	0.138	0.453	0	0	0	0.114	0.276	0.122	0.175	0.123	0.413	10	0.2094	0.153	812.1
PLCH2	0.111	0.172	0.103	0.103	0	0	0.288	0	0	0	0.202	0.125	0.116	0.178	0	0.3	10	0.1698	0.1485	2500.3
RLN2	0.109	0.144	0.132	0.131	0.127	0.136	0.4	0	0	0	0	0.189	0	0.147	0	0.412	10	0.1927	0.14	1288.3
YIF1A	0.109	0.123	0.113	0.113	0	0.142	0.417	0	0	0	0.134	0.14	0	0.126	0	0.396	10	0.1813	0.13	1766.4
RAPGEF2	0.109	0.168	0.118	0.118	0	0	0.297	0	0	0	0.178	0.196	0	0.171	0.124	0.325	10	0.1804	0.1695	1722.3
RELL2	0.107	0.149	0.143	0.143	0	0	0.385	0	0	0	0.129	0.165	0	0.154	0.112	0.38	10	0.1867	0.146	1447.9
RELL1	0.107	0.149	0.143	0.143	0	0	0.385	0	0	0	0.129	0.165	0	0.154	0.112	0.38	10	0.1867	0.146	1448.9
TMEM33	0.106	0.127	0.113	0.112	0	0.242	0.31	0	0	0.105	0	0.17	0	0.128	0	0.38	10	0.1793	0.1275	1711.5
GDPD5	0.106	0.126	0.136	0.136	0.231	0	0.344	0	0	0	0.111	0.107	0	0.13	0	0.297	10	0.1724	0.133	2476.7

ATP6V1E2	0.106	0.158	0.143	0.143	0	0.184	0.326	0	0	0	0.124	0.162	0	0.16	0	0.358	10	0.1864	0.159	1729.3
REXO1	0.106	0.102	0	0	0	0	0.418	0.103	0.105	0.108	0	0.213	0.113	0.108	0	0.386	10	0.1762	0.108	1104.8
POLR2J	0.106	0.13	0.117	0.117	0	0	0.461	0	0	0	0.134	0.237	0.126	0.134	0	0.436	10	0.1998	0.132	777.9
NETO2	0.105	0.146	0.108	0.108	0	0.157	0.371	0	0	0	0.159	0.183	0	0.149	0	0.43	10	0.1916	0.153	1363.3
VSNL1	0.104	0.148	0.136	0.136	0	0.101	0.377	0	0	0	0.142	0.152	0	0.153	0	0.343	10	0.1792	0.145	2115.5
DNM3	0.102	0.126	0.127	0.127	0	0.147	0.431	0	0	0	0	0.217	0	0.131	0.141	0.431	10	0.198	0.136	1047.8
TPST2	0.101	0.132	0.131	0.131	0.337	0.181	0.278	0	0	0	0	0.151	0	0.134	0	0.319	10	0.1895	0.1425	2242.9
LCA5L	0.101	0.111	0	0	0.104	0.204	0.34	0.219	0.222	0	0	0.234	0	0.115	0	0.363	10	0.2013	0.2115	1233.9
SERPINA7	0	0.109	0.107	0.106	0.195	0.106	0.316	0	0	0	0.135	0.116	0	0.11	0	0.31	10	0.161	0.113	2908.1
IGHV4-59	0	0.145	0.156	0.156	0.208	0.11	0.214	0	0	0	0.192	0.127	0	0.147	0	0.287	10	0.1742	0.156	3230.5
SHANK2	0	0.135	0.123	0.122	0	0.375	0.353	0	0	0	0.106	0.345	0	0.139	0.142	0.405	10	0.2245	0.1405	883.1
GNA11	0.377	0.513	0.419	0.419	0	0	0	0	0	0	0.956	0	0.415	0.513	0.142	0.373	9	0.458555556	0.419	274.4444444
GNAQ	0.374	0.507	0.411	0.411	0	0	0	0	0	0	0.961	0	0.418	0.507	0.138	0.362	9	0.454333333	0.411	316.5555556
HRH1	0.283	0.36	0.228	0.228	0	0	0.148	0	0	0	0.192	0	0.177	0.361	0	0.375	9	0.261333333	0.228	1924.666667
GNG5	0.267	0.298	0.249	0.249	0	0	0.237	0	0	0	0.32	0	0.181	0.3	0	0.387	9	0.276444444	0.267	1311.888889
GNAL	0.265	0.316	0.24	0.24	0	0	0.145	0	0	0	0.342	0	0.189	0.317	0	0.352	9	0.267333333	0.265	2009.222222
PLCB4	0.254	0.344	0.311	0.311	0	0	0.192	0	0	0	0.522	0	0.254	0.346	0	0.334	9	0.318666667	0.311	1746.555556
NPFFR2	0.223	0.303	0.186	0.186	0.105	0	0	0	0	0	0.193	0	0.189	0.303	0	0.311	9	0.222111111	0.193	681.3333333
UTS2B	0.219	0.301	0.179	0.179	0	0	0.22	0	0	0	0	0.106	0.145	0.304	0	0.353	9	0.222888889	0.219	2897.888889
GPR55	0.213	0.194	0	0	0	0	0.105	0.105	0.107	0.101	0	0	0.106	0.196	0	0.216	9	0.149222222	0.107	3236.777778
OPRL1	0.2	0.292	0.191	0.191	0	0	0.147	0	0	0	0.163	0	0.122	0.293	0	0.339	9	0.215333333	0.191	2155.111111
TBXA2R	0.192	0.295	0.224	0.224	0	0	0.105	0	0	0	0.404	0	0.128	0.296	0	0.285	9	0.239222222	0.224	2626.555556
GPRIN1	0.185	0.249	0.211	0.211	0	0	0.129	0	0	0	0.493	0	0.174	0.25	0	0.245	9	0.238555556	0.211	2745.666667
KCNJ5	0.183	0.265	0.173	0.173	0	0	0.143	0	0	0	0.334	0	0.167	0.265	0	0.33	9	0.225888889	0.183	2190.555556
AGTRAP	0.18	0.247	0.206	0.206	0	0	0.159	0	0	0	0.148	0.117	0	0.25	0	0.245	9	0.195333333	0.206	3759.888889
MRGPRX3	0.174	0.216	0.141	0.141	0	0	0.192	0	0	0	0.167	0	0.11	0.217	0	0.286	9	0.182666667	0.174	2232.222222
KCNJ3	0.17	0.242	0.157	0.157	0	0	0.122	0	0	0	0.275	0	0.16	0.242	0	0.292	9	0.201888889	0.17	2554.555556
GPBAR1	0.169	0.208	0.113	0.113	0	0.241	0.213	0	0	0	0	0.275	0	0.212	0	0.274	9	0.202	0.212	2219.222222
MCHR2	0.16	0.204	0.153	0.153	0.133	0.143	0.201	0	0	0	0	0	0	0.205	0	0.338	9	0.187777778	0.16	2048.444444
RASD1	0.158	0.2	0.119	0.119	0	0	0.309	0	0	0	0.245	0.161	0	0.205	0	0.323	9	0.204333333	0.2	1962.222222

FSHR	0.157	0.217	0.275	0.275	0.567	0.111	0.107	0	0	0	0	0	0	0.218	0	0.289	9	0.246222222	0.218	2998.444444
PDE6G	0.157	0.176	0	0	0	0	0.175	0.298	0.3	0	0.134	0	0.126	0.178	0	0.272	9	0.201777778	0.176	2389.888889
RAB43	0.156	0.15	0	0	0	0.138	0.422	0	0	0	0.139	0.191	0	0.156	0.126	0.396	9	0.208222222	0.156	1287.666667
CPM	0.154	0.137	0.137	0.137	0	0.111	0.229	0	0	0	0	0.1	0	0.138	0	0.251	9	0.154888889	0.137	4057.333333
CD97	0.15	0.171	0	0	0.133	0.188	0.338	0	0	0	0.1	0.205	0	0.174	0	0.401	9	0.206666667	0.174	1272.111111
SNX1	0.149	0.187	0	0	0	0.176	0.403	0	0	0	0	0.221	0.104	0.192	0.158	0.414	9	0.222666667	0.187	904.6666667
MKNK2	0.149	0.157	0	0	0	0	0.433	0	0	0	0.106	0.254	0.147	0.17	0.1	0.327	9	0.204777778	0.157	1030.777778
AKAP11	0.145	0.193	0	0	0	0.134	0.502	0	0	0	0.151	0.214	0.194	0.203	0	0.434	9	0.241111111	0.194	844.6666667
INSC	0.144	0.185	0.112	0.112	0	0	0.428	0	0	0	0.277	0.22	0	0.19	0	0.458	9	0.236222222	0.19	749.6666667
PDE6D	0.144	0.152	0	0	0	0	0.175	0.31	0.311	0	0.13	0	0.118	0.153	0	0.233	9	0.191777778	0.153	2708.555556
NMUR2	0.143	0.192	0.155	0.155	0	0.113	0.214	0	0	0	0	0.156	0	0.195	0	0.333	9	0.184	0.156	2950
MTNR1B	0.14	0.174	0.133	0.133	0.157	0	0.171	0	0	0	0	0.109	0	0.175	0	0.268	9	0.162222222	0.157	3747.888889
GPR4	0.138	0.191	0.11	0.11	0	0	0.29	0	0	0	0.159	0.157	0	0.197	0	0.296	9	0.183111111	0.159	2320.555556
CLUL1	0.137	0.161	0	0	0	0.121	0.366	0.222	0.225	0	0	0.182	0	0.163	0	0.441	9	0.224222222	0.182	1483.111111
AZGP1P2	0.137	0.164	0	0	0.108	0.143	0.367	0	0	0	0.125	0.163	0	0.165	0	0.434	9	0.200666667	0.163	1592.555556
AZGP1P1	0.137	0.164	0	0	0.108	0.143	0.367	0	0	0	0.125	0.163	0	0.165	0	0.434	9	0.200666667	0.163	1593.555556
PDE9A	0.136	0.169	0.113	0.113	0	0.148	0.261	0	0	0	0	0.16	0	0.173	0	0.323	9	0.177333333	0.16	2493.666667
RAB21	0.134	0.138	0	0	0	0.15	0.41	0	0	0	0.106	0.226	0	0.143	0.174	0.385	9	0.207333333	0.15	1108.333333
CHMP4A	0.134	0.14	0	0	0	0.145	0.479	0	0	0	0	0.221	0.105	0.145	0.118	0.444	9	0.214555556	0.145	950.4444444
MBOAT2	0.133	0.174	0.102	0.102	0	0	0.254	0	0	0	0.231	0	0.129	0.176	0	0.265	9	0.174	0.174	2063.777778
GPR110	0.132	0.19	0.163	0.163	0.115	0.102	0.166	0	0	0	0	0	0	0.191	0	0.287	9	0.167666667	0.163	2902.333333
TMED2	0.132	0.215	0.156	0.156	0	0.142	0.278	0	0	0	0	0.135	0	0.217	0	0.363	9	0.199333333	0.156	2412
RINL	0.13	0.155	0	0	0	0.114	0.336	0	0	0	0.173	0.218	0	0.159	0.343	0.342	9	0.218888889	0.173	1688.111111
MAS1	0.128	0.199	0.192	0.192	0	0	0.169	0	0	0	0.153	0.106	0	0.201	0	0.248	9	0.176444444	0.192	3877.111111
SLC25A42	0.127	0.18	0.128	0.128	0	0	0.225	0	0	0	0.193	0	0.149	0.181	0	0.255	9	0.174	0.18	2257.555556
GIT1	0.127	0.144	0.103	0.103	0	0	0.278	0	0	0	0	0.166	0	0.155	0.644	0.262	9	0.220222222	0.155	2560.333333
CABP4	0.126	0.121	0	0	0	0	0.246	0.281	0.284	0	0	0.118	0.101	0.123	0	0.29	9	0.187777778	0.126	3112.222222
NCS1	0.126	0.177	0	0	0	0.129	0.401	0	0	0	0.188	0.166	0.102	0.182	0	0.388	9	0.206555556	0.177	1593.666667
AAMP	0.125	0.193	0.142	0.142	0	0	0.319	0	0	0	0.243	0.165	0	0.197	0	0.366	9	0.210222222	0.193	1700.555556
OR6A2	0.125	0.172	0.167	0.167	0	0	0.286	0	0	0	0.134	0.112	0	0.175	0	0.377	9	0.190555556	0.167	2455.666667

PDE1A	0.125	0.18	0	0	0	0.134	0.398	0	0	0	0.155	0.222	0.141	0.189	0	0.373	9	0.213	0.18	1157.444444
GPR123	0.124	0.148	0.114	0.113	0	0.145	0.317	0	0	0	0	0.182	0	0.149	0	0.385	9	0.186333333	0.148	1808.222222
SLC6A18	0.123	0.18	0.14	0.14	0	0.214	0.254	0	0	0	0	0.148	0	0.183	0	0.306	9	0.187555556	0.18	2560.111111
PITPNC1	0.123	0.184	0	0	0	0.131	0.462	0	0	0	0.193	0.253	0.103	0.188	0	0.443	9	0.231111111	0.188	796.5555556
GIPC1	0.123	0.169	0	0	0	0.163	0.275	0	0	0	0.187	0.139	0.125	0.172	0	0.347	9	0.188888889	0.169	2421.222222
PRSS22	0.121	0	0.113	0.113	0	0	0.258	0.151	0.153	0.158	0	0	0.103	0	0	0.235	9	0.156111111	0.151	2195
REEP2	0.121	0.165	0.119	0.119	0	0.114	0.242	0	0	0	0	0.161	0	0.167	0	0.328	9	0.170666667	0.161	2836.444444
BEST3	0.121	0.153	0	0	0	0.291	0.345	0.123	0.126	0	0	0.259	0	0.158	0	0.333	9	0.212111111	0.158	1168.444444
UNC119	0.12	0.125	0	0	0	0	0.266	0.208	0.21	0	0	0.116	0.107	0.131	0	0.285	9	0.174222222	0.131	3022.777778
RAB1A	0.12	0.168	0	0	0	0.189	0.44	0	0	0	0.158	0.24	0.145	0.172	0	0.433	9	0.229444444	0.172	683.5555556
LOC100129726	0.119	0.129	0.136	0.136	0.414	0	0	0.127	0.129	0	0	0	0	0.13	0	0.194	9	0.168222222	0.13	1694.888889
OR1G1	0.119	0.148	0.136	0.136	0	0.127	0.242	0	0	0	0	0.118	0	0.15	0	0.315	9	0.165666667	0.136	3253.666667
VPS26A	0.118	0.169	0	0	0	0.148	0.506	0	0	0	0.11	0.258	0.1	0.173	0	0.489	9	0.230111111	0.169	696.5555556
DYNC1LI2	0.118	0.106	0	0	0	0.123	0.437	0	0	0	0	0.276	0.103	0.115	0.128	0.454	9	0.206666667	0.123	1072.666667
CHM	0.116	0.111	0	0	0	0.107	0.294	0.204	0.209	0	0	0.124	0	0.112	0	0.309	9	0.176222222	0.124	3119.555556
KCTD16	0.115	0.159	0.14	0.14	0	0.151	0.285	0	0	0	0	0.127	0	0.161	0	0.377	9	0.183888889	0.151	2479.333333
PPAP2C	0.115	0.153	0	0	0	0.165	0.442	0	0	0	0.125	0.221	0.11	0.159	0	0.406	9	0.210666667	0.159	941.4444444
KIAA1033	0.115	0.138	0	0	0	0.159	0.539	0	0	0	0	0.267	0.125	0.144	0.125	0.508	9	0.235555556	0.144	646
TRIO	0.115	0.152	0	0	0	0	0.163	0	0	0	0.278	0.134	0.102	0.154	0.338	0.208	9	0.182666667	0.154	3968.444444
GPRC5B	0.114	0.132	0.115	0.114	0	0.118	0.24	0	0	0	0	0.112	0	0.133	0	0.296	9	0.152666667	0.118	3598.333333
BRSK1	0.114	0.106	0	0	0	0	0.391	0	0.1	0.113	0	0.216	0	0.118	0.198	0.287	9	0.182555556	0.118	1572.111111
RASD2	0.113	0.153	0.105	0.105	0.185	0	0.164	0	0	0	0.131	0	0	0.154	0	0.26	9	0.152222222	0.153	2743.555556
IFT52	0.113	0.166	0	0	0.104	0.211	0.266	0	0	0	0.144	0.199	0	0.169	0	0.349	9	0.191222222	0.169	1866.777778
PPP5C	0.113	0.162	0	0	0	0	0.301	0	0	0	0.239	0.151	0.126	0.168	0.144	0.31	9	0.190444444	0.162	2252.666667
GPHB5	0.112	0.135	0.204	0.204	0.853	0.113	0.109	0	0	0	0	0	0	0.136	0	0.204	9	0.23	0.136	3736
NLN	0.112	0.14	0.109	0.109	0	0.146	0.348	0	0	0	0	0.115	0	0.142	0	0.351	9	0.174666667	0.14	2500.555556
VN1R1	0.112	0.162	0.111	0.111	0	0	0.173	0	0	0	0.159	0	0.103	0.163	0	0.279	9	0.152555556	0.159	2580
TNFAIP8L1	0.111	0.153	0.119	0.119	0	0	0.119	0	0	0	0.31	0	0.106	0.154	0	0.178	9	0.152111111	0.119	3577.111111
COPE	0.111	0.13	0	0	0	0.213	0.562	0	0	0	0.109	0.275	0	0.137	0.137	0.479	9	0.239222222	0.137	579.2222222
CABP5	0.11	0.138	0	0	0	0.106	0.321	0.156	0.159	0	0	0.153	0	0.143	0	0.358	9	0.182666667	0.153	2374.666667

HSD3BP2	0.109	0.157	0.166	0.166	0.425	0	0.119	0	0	0	0.128	0	0	0.158	0	0.248	9	0.186222222	0.158	2987.444444
RAB35	0.109	0.127	0	0	0	0.11	0.387	0	0	0	0.119	0.197	0	0.132	0.196	0.341	9	0.190888889	0.132	1816.777778
VN1R4	0.108	0.17	0.117	0.117	0	0.113	0.175	0	0	0	0.203	0	0	0.169	0	0.304	9	0.164	0.169	2725.888889
VN1R5	0.108	0.17	0.117	0.117	0	0.113	0.175	0	0	0	0.203	0	0	0.169	0	0.304	9	0.164	0.169	2726.888889
VN1R2	0.108	0.17	0.117	0.117	0	0.113	0.175	0	0	0	0.203	0	0	0.169	0	0.304	9	0.164	0.169	2727.888889
VN1R3	0.108	0.17	0.117	0.117	0	0.113	0.175	0	0	0	0.203	0	0	0.169	0	0.304	9	0.164	0.169	2728.888889
GABRR1	0.108	0.129	0.115	0.115	0	0.176	0.208	0	0	0	0	0.134	0	0.132	0	0.275	9	0.154666667	0.132	3421.222222
TBC1D5	0.108	0.117	0	0	0	0.125	0.458	0	0	0	0	0.203	0.122	0.122	0.112	0.42	9	0.198555556	0.122	1339
GPR161	0.107	0.129	0.128	0.128	0	0.108	0.228	0	0	0	0	0.133	0	0.13	0	0.291	9	0.153555556	0.129	3571.555556
PIP4K2A	0.107	0.131	0	0	0	0.124	0.329	0	0	0	0.151	0.176	0.119	0.137	0	0.345	9	0.179888889	0.137	2070
GPHA2	0.106	0.135	0.186	0.186	0.858	0.118	0.111	0	0	0	0	0	0	0.136	0	0.209	9	0.227222222	0.136	3666
GPRC5C	0.106	0.13	0.112	0.112	0	0.15	0.281	0	0	0	0	0.121	0	0.131	0	0.316	9	0.162111111	0.13	2938.111111
RAB11FIP3	0.106	0.116	0	0	0	0.119	0.343	0	0	0	0	0.215	0.104	0.125	0.258	0.363	9	0.194333333	0.125	1725
NRSN2	0.105	0.126	0.171	0.171	0.832	0.116	0.154	0	0	0	0	0	0	0.127	0	0.21	9	0.223555556	0.154	3459
TBC1D3G	0.105	0.104	0	0	0	0	0.265	0.12	0.121	0.13	0	0.176	0	0.106	0	0.286	9	0.157	0.121	2535
TBC1D3H	0.105	0.104	0	0	0	0	0.265	0.12	0.121	0.13	0	0.176	0	0.106	0	0.286	9	0.157	0.121	2534
TBC1D3B	0.105	0.104	0	0	0	0	0.265	0.12	0.121	0.13	0	0.176	0	0.106	0	0.286	9	0.157	0.121	2536
LOC653380	0.105	0.104	0	0	0	0	0.265	0.12	0.121	0.13	0	0.176	0	0.106	0	0.286	9	0.157	0.121	2533
MTMR6	0.105	0.126	0	0	0	0.186	0.473	0	0	0	0.121	0.306	0	0.135	0.122	0.406	9	0.22	0.135	724.6666667
IQGAP3	0.105	0.15	0	0	0	0	0.303	0	0	0	0.19	0.222	0.112	0.154	0.182	0.315	9	0.192555556	0.182	1673.555556
NMB	0.104	0.179	0.1	0	0.197	0.12	0.199	0	0	0	0	0.117	0	0.18	0	0.34	9	0.170666667	0.179	3469
SLC24A1	0.104	0.148	0	0	0	0.208	0.345	0.158	0.161	0	0	0.185	0	0.151	0	0.398	9	0.206444444	0.161	1396.333333
MAPK10	0.104	0.103	0	0	0	0	0.223	0.134	0.135	0.128	0	0.127	0	0.114	0	0.195	9	0.140333333	0.128	3919
GGA2	0.102	0.104	0	0	0	0.137	0.422	0	0	0	0	0.163	0.106	0.107	0.104	0.393	9	0.182	0.107	1812.222222
RAB11B	0.101	0.134	0	0	0	0.572	0.419	0	0	0	0.114	0.542	0	0.139	0.13	0.404	9	0.283888889	0.139	717.6666667
PI4KA	0.1	0.162	0.117	0.117	0	0.133	0.417	0	0	0	0	0.168	0	0.168	0	0.369	9	0.194555556	0.162	1646.555556
CABIN1	0.1	0.124	0.179	0.179	0	0	0.334	0	0	0	0.113	0.194	0	0.128	0	0.353	9	0.189333333	0.179	1619.333333
SLC5A5	0	0.115	0.153	0.153	0.714	0.159	0.222	0	0	0	0	0.133	0	0.118	0	0.259	9	0.225111111	0.153	3386.666667
RLN1	0	0.149	0.122	0.122	0.115	0.134	0.364	0	0	0	0	0.178	0	0.151	0	0.394	9	0.192111111	0.149	1613.333333
MVK	0	0.127	0.137	0.137	0.296	0.13	0.332	0	0	0	0	0.165	0	0.129	0	0.317	9	0.196666667	0.137	2168.666667

GLUD1P5	0	0.11	0.107	0.106	0.146	0.131	0.393	0	0	0	0	0.137	0	0.113	0	0.372	9	0.179444444	0.131	2145.555556
KCNIP1	0	0.119	0.118	0.118	0.204	0.229	0.325	0	0	0	0	0.165	0	0.122	0	0.393	9	0.199222222	0.165	1741.888889
CGB1	0	0.111	0.149	0.149	0.372	0.123	0.288	0	0	0	0	0.112	0	0.113	0	0.352	9	0.196555556	0.149	2935.666667
T1560	0	0.113	0.154	0.154	0.641	0.114	0.219	0	0	0	0	0.145	0	0.115	0	0.314	9	0.218777778	0.154	3222.555556
PRKAR1AP	0	0.116	0.11	0.11	0	0.104	0.38	0	0	0	0.147	0.144	0	0.119	0	0.403	9	0.181444444	0.119	2258
GPR83	0	0.142	0.119	0.119	0	0.101	0.305	0	0	0	0.108	0.214	0	0.145	0	0.321	9	0.174888889	0.142	2243.666667
ARF4P1	0	0.15	0.143	0.143	0	0.176	0.395	0	0	0	0.154	0.188	0	0.153	0	0.42	9	0.213555556	0.154	1173.555556
FOLR1	0	0.138	0.113	0.113	0	0.125	0.419	0	0	0	0.195	0.206	0	0.141	0	0.38	9	0.203333333	0.141	1339.222222
CATSPER1	0	0.165	0.152	0.152	0	0.237	0.349	0	0	0	0.117	0.266	0	0.17	0	0.387	9	0.221666667	0.17	947.6666667
KCND2	0	0.132	0.11	0.11	0	0.197	0.311	0	0	0	0.13	0.159	0	0.135	0	0.372	9	0.184	0.135	1974.333333
SAR1A	0	0.165	0.13	0.13	0	0.184	0.224	0	0	0	0.157	0.17	0	0.166	0	0.286	9	0.179111111	0.166	2691.666667
GPR116	0	0.122	0.104	0.104	0	0.14	0.123	0	0	0	0.207	0	0.106	0.122	0	0.172	9	0.133333333	0.122	3863.555556
RAP1GAP2	0	0.167	0.108	0.108	0	0	0.269	0	0	0	0.288	0.124	0.147	0.173	0	0.246	9	0.181111111	0.167	3107.111111
SH3YL1	0	0.115	0.101	0.1	0	0.11	0.273	0	0	0	0	0.181	0	0.119	0.188	0.274	9	0.162333333	0.119	2919
SSH1	0	0.103	0	0	0	0	0.296	0.115	0.117	0.105	0	0.2	0	0.112	0.299	0.278	9	0.180555556	0.117	2113.555556
PTK2B	0	0.136	0.108	0.108	0	0	0.232	0	0	0	0.112	0.182	0	0.149	0.302	0.245	9	0.174888889	0.149	2862.666667
FLOT1	0	0.124	0	0	0	0.113	0.315	0	0	0	0.115	0.172	0.124	0.129	0.143	0.255	9	0.165555556	0.129	2772.666667

Table S3. GeneIndexer latent semantic data extraction of DDR system-associated proteins. For each specific protein, identified by its official gene symbol, the cosine similarity score (at least >0.1, therefore indicating an implicit association) for the strength of latent semantic association with the DDR-specific concept terms (from Table S1) is given. The sum of the specific concept occurrences, the average and median cosine similarity score and the average rank within the matrix are given for each specific protein.

Gene symbol	DNA damage response	DNA damage	Double-strand break	DNA damage repair	Single-strand break	Base excision repair	Nucleotide excision repair	Homologous recombination	Non-homologous end-joining	DSB	SSB	Genotoxic stress	Genotoxic	DDR	Senescence	Cell-cycle checkpoint	Cell cycle checkpoint	Sum of occurrences	Average score	Median score	Average rank
TP53BP1	0.735	0.731	0.694	0.613	0.679	0.12	0.132	0.518	0.49	0.783	0.174	0.572	0.581	0.712	0.243	0.469	0.487	17	0.513705882	0.572	190.1764706
TOPBP1	0.706	0.705	0.353	0.537	0.342	0.137	0.116	0.236	0.124	0.376	0.188	0.519	0.525	0.681	0.119	0.657	0.861	17	0.422470588	0.376	363.9411765
H2AFX	0.699	0.7	0.728	0.605	0.718	0.114	0.148	0.622	0.426	0.776	0.145	0.468	0.473	0.728	0.16	0.433	0.515	17	0.497529412	0.515	229.4705882
POLQ	0.641	0.644	0.535	0.64	0.522	0.499	0.417	0.392	0.31	0.443	0.21	0.406	0.399	0.376	0.147	0.302	0.275	17	0.421058824	0.406	154.4117647
RAD9B	0.63	0.62	0.339	0.508	0.319	0.239	0.173	0.32	0.102	0.243	0.159	0.487	0.481	0.448	0.166	0.594	0.685	17	0.383117647	0.339	227.2941176
REV3L	0.626	0.625	0.346	0.603	0.335	0.447	0.489	0.26	0.16	0.232	0.127	0.461	0.447	0.274	0.163	0.378	0.392	17	0.374411765	0.378	220.9411765
CDC45	0.583	0.58	0.375	0.491	0.366	0.185	0.17	0.281	0.137	0.26	0.23	0.425	0.427	0.415	0.127	0.578	0.736	17	0.374470588	0.375	251.6470588
NABP2	0.582	0.582	0.612	0.512	0.602	0.132	0.135	0.624	0.239	0.578	0.173	0.45	0.456	0.625	0.149	0.329	0.332	17	0.418352941	0.456	243.5294118
WRN	0.58	0.592	0.688	0.584	0.691	0.301	0.272	0.737	0.309	0.54	0.185	0.34	0.338	0.375	0.146	0.314	0.393	17	0.434411765	0.375	152.8823529
NABP1	0.558	0.56	0.635	0.498	0.625	0.124	0.131	0.667	0.237	0.6	0.169	0.395	0.402	0.64	0.123	0.299	0.331	17	0.411411765	0.402	299.7647059
POLA1	0.554	0.547	0.369	0.479	0.356	0.33	0.265	0.343	0.145	0.227	0.157	0.356	0.342	0.285	0.15	0.449	0.509	17	0.344882353	0.343	216.2941176
KIN	0.553	0.562	0.381	0.545	0.371	0.541	0.376	0.339	0.141	0.217	0.233	0.325	0.307	0.203	0.126	0.324	0.3	17	0.343764706	0.325	256.7647059
RECQL4	0.548	0.535	0.57	0.511	0.556	0.255	0.245	0.559	0.272	0.427	0.164	0.422	0.412	0.279	0.224	0.363	0.336	17	0.392823529	0.412	133.8235294
IGHV3-7	0.526	0.539	0.353	0.628	0.356	0.555	0.505	0.207	0.203	0.285	0.261	0.339	0.348	0.168	0.105	0.229	0.232	17	0.343470588	0.339	381.9411765
AIFM2	0.523	0.498	0.354	0.405	0.333	0.212	0.181	0.268	0.205	0.305	0.144	0.475	0.438	0.313	0.233	0.29	0.261	17	0.319882353	0.305	237.0588235
POLE4	0.505	0.495	0.373	0.424	0.361	0.2	0.185	0.373	0.15	0.271	0.122	0.316	0.3	0.348	0.131	0.435	0.497	17	0.322705882	0.348	275.1176471
SESN1	0.499	0.464	0.303	0.399	0.292	0.174	0.132	0.154	0.149	0.227	0.346	0.492	0.452	0.252	0.216	0.327	0.325	17	0.306058824	0.303	455.6470588
POLM	0.497	0.492	0.523	0.498	0.512	0.366	0.317	0.32	0.466	0.45	0.15	0.264	0.248	0.212	0.105	0.224	0.217	17	0.344764706	0.32	334.2352941
QRSL1	0.496	0.474	0.381	0.374	0.36	0.123	0.115	0.153	0.238	0.473	0.193	0.357	0.33	0.625	0.109	0.21	0.176	17	0.305117647	0.33	653.8235294
GATC	0.496	0.474	0.381	0.374	0.36	0.123	0.115	0.153	0.238	0.473	0.193	0.357	0.33	0.625	0.109	0.21	0.176	17	0.305117647	0.33	654.8235294
PIF1	0.46	0.466	0.484	0.491	0.487	0.252	0.274	0.509	0.202	0.391	0.186	0.248	0.248	0.336	0.267	0.229	0.286	17	0.342117647	0.286	212.0588235
INO80	0.456	0.446	0.535	0.46	0.532	0.203	0.207	0.568	0.246	0.392	0.244	0.302	0.287	0.256	0.138	0.309	0.28	17	0.344764706	0.302	231.7058824
SETX	0.41	0.402	0.445	0.379	0.427	0.208	0.166	0.419	0.193	0.326	0.211	0.258	0.24	0.269	0.159	0.182	0.207	17	0.288294118	0.258	343.9411765

UBR2	0.405	0.391	0.381	0.342	0.366	0.12	0.11	0.406	0.196	0.365	0.115	0.303	0.291	0.271	0.125	0.273	0.24	17	0.276470588	0.291	466.5294118
HNRNPUL2	0.399	0.393	0.45	0.35	0.445	0.125	0.125	0.391	0.321	0.458	0.105	0.24	0.238	0.33	0.257	0.275	0.301	17	0.306058824	0.321	366.5882353
HIST1H2AA	0.385	0.383	0.345	0.32	0.324	0.126	0.119	0.329	0.138	0.238	0.103	0.272	0.26	0.197	0.111	0.325	0.245	17	0.248235294	0.26	525.0588235
ZNF350	0.382	0.381	0.318	0.405	0.321	0.284	0.171	0.234	0.14	0.32	0.388	0.227	0.23	0.226	0.144	0.217	0.203	17	0.270058824	0.234	413.2941176
HVBS7	0.367	0.337	0.319	0.297	0.309	0.135	0.116	0.261	0.211	0.263	0.164	0.292	0.27	0.201	0.28	0.238	0.164	17	0.248470588	0.263	457.8823529
HIST2H2AA3	0.355	0.345	0.358	0.296	0.35	0.161	0.14	0.34	0.144	0.269	0.134	0.234	0.224	0.223	0.138	0.202	0.181	17	0.240823529	0.224	480.4117647
OA23	0.328	0.322	0.268	0.308	0.268	0.193	0.231	0.248	0.112	0.188	0.103	0.215	0.213	0.166	0.104	0.142	0.164	17	0.210176471	0.213	695.6470588
BFHD	0.328	0.322	0.268	0.308	0.268	0.193	0.231	0.248	0.112	0.188	0.103	0.215	0.213	0.166	0.104	0.142	0.164	17	0.210176471	0.213	693.6470588
OA21	0.328	0.322	0.268	0.308	0.268	0.193	0.231	0.248	0.112	0.188	0.103	0.215	0.213	0.166	0.104	0.142	0.164	17	0.210176471	0.213	692.6470588
OA22	0.328	0.322	0.268	0.308	0.268	0.193	0.231	0.248	0.112	0.188	0.103	0.215	0.213	0.166	0.104	0.142	0.164	17	0.210176471	0.213	694.6470588
SNORA45	0.275	0.26	0.267	0.246	0.26	0.157	0.155	0.315	0.124	0.191	0.113	0.146	0.124	0.106	0.158	0.162	0.134	17	0.187823529	0.158	861.1176471
SNORA12	0.275	0.26	0.267	0.246	0.26	0.157	0.155	0.315	0.124	0.191	0.113	0.146	0.124	0.106	0.158	0.162	0.134	17	0.187823529	0.158	860.1176471
SNORA81	0.275	0.26	0.267	0.246	0.26	0.157	0.155	0.315	0.124	0.191	0.113	0.146	0.124	0.106	0.158	0.162	0.134	17	0.187823529	0.158	859.1176471
SNORA74B	0.275	0.26	0.267	0.246	0.26	0.157	0.155	0.315	0.124	0.191	0.113	0.146	0.124	0.106	0.158	0.162	0.134	17	0.187823529	0.158	862.1176471
SCARNA18	0.275	0.26	0.267	0.246	0.26	0.157	0.155	0.315	0.124	0.191	0.113	0.146	0.124	0.106	0.158	0.162	0.134	17	0.187823529	0.158	863.1176471
SNORA5C	0.275	0.26	0.267	0.246	0.26	0.157	0.155	0.315	0.124	0.191	0.113	0.146	0.124	0.106	0.158	0.162	0.134	17	0.187823529	0.158	864.1176471
SNORA11	0.275	0.26	0.267	0.246	0.26	0.157	0.155	0.315	0.124	0.191	0.113	0.146	0.124	0.106	0.158	0.162	0.134	17	0.187823529	0.158	858.1176471
SMC2	0.263	0.253	0.265	0.226	0.256	0.121	0.107	0.283	0.106	0.171	0.113	0.159	0.152	0.178	0.151	0.307	0.319	17	0.201764706	0.178	761.7647059
RAD9A	0.721	0.724	0.373	0.578	0.358	0.275	0.196	0.25	0.127	0.323	0.184	0.502	0.5	0.613	0	0.59	0.749	16	0.4414375	0.4365	165.1875
ATMIN	0.715	0.716	0.452	0.575	0.438	0.148	0	0.213	0.239	0.505	0.275	0.592	0.606	0.806	0.131	0.513	0.653	16	0.4735625	0.509	243.9375
HUS1	0.674	0.682	0.328	0.559	0.315	0.32	0.213	0.237	0	0.239	0.192	0.471	0.47	0.498	0.1	0.551	0.701	16	0.409375	0.399	295.4375
MUS81	0.651	0.65	0.426	0.631	0.422	0.283	0.438	0.422	0.129	0.34	0	0.478	0.483	0.484	0.122	0.523	0.694	16	0.4485	0.458	184.9375
NBN	0.644	0.649	0.679	0.571	0.673	0	0.135	0.641	0.317	0.696	0.165	0.425	0.435	0.737	0.116	0.418	0.54	16	0.4900625	0.5555	208.25
RECQL	0.618	0.62	0.737	0.63	0.74	0.392	0.315	0.688	0.211	0.477	0.436	0.405	0.406	0.407	0	0.266	0.292	16	0.4775	0.4215	95.75
EME1	0.614	0.618	0.397	0.585	0.396	0.281	0.419	0.407	0.113	0.341	0.11	0.394	0.401	0.476	0	0.497	0.7	16	0.4218125	0.404	158.375
MRE11A	0.609	0.614	0.705	0.554	0.7	0	0.147	0.676	0.357	0.73	0.119	0.378	0.386	0.687	0.112	0.363	0.459	16	0.47475	0.5065	231.75
RAD50	0.596	0.599	0.684	0.532	0.679	0	0.126	0.662	0.354	0.715	0.125	0.366	0.374	0.664	0.117	0.366	0.463	16	0.463875	0.4975	247.8125
INTS3	0.587	0.591	0.711	0.548	0.707	0.141	0.16	0.779	0.261	0.637	0.201	0.391	0.394	0.636	0	0.326	0.369	16	0.4649375	0.471	155.25
EME2	0.586	0.595	0.496	0.536	0.49	0.245	0.25	0.566	0.152	0.349	0.138	0.371	0.374	0.431	0	0.466	0.613	16	0.416125	0.4485	132.875
INIP	0.584	0.588	0.713	0.546	0.709	0.135	0.155	0.779	0.261	0.641	0.2	0.385	0.389	0.643	0	0.322	0.365	16	0.4634375	0.4675	166.3125

DCLRE1B	0.576	0.578	0.647	0.598	0.64	0.246	0.401	0.578	0.448	0.649	0	0.362	0.366	0.474	0.199	0.303	0.336	16	0.4625625	0.461	119.375
HORMAD1	0.563	0.562	0.5	0.489	0.493	0.115	0.119	0.53	0.191	0.489	0.177	0.345	0.345	0.506	0	0.429	0.554	16	0.4004375	0.489	268.9375
ASF1A	0.552	0.537	0.351	0.409	0.334	0.11	0.113	0.33	0.158	0.328	0	0.381	0.366	0.478	0.184	0.513	0.579	16	0.3576875	0.3585	355.375
TREX2	0.551	0.545	0.555	0.485	0.54	0.208	0.169	0.446	0.324	0.622	0.149	0.326	0.322	0.624	0	0.217	0.213	16	0.3935	0.386	210.6875
SETMAR	0.544	0.541	0.454	0.448	0.443	0.13	0.133	0.438	0.259	0.386	0.124	0.35	0.348	0.478	0	0.493	0.648	16	0.3885625	0.4405	237.125
FANCD2	0.538	0.538	0.55	0.525	0.547	0.154	0.211	0.655	0.21	0.399	0.102	0.372	0.373	0.408	0	0.399	0.484	16	0.4040625	0.4035	180.4375
LIG1	0.528	0.535	0.477	0.708	0.481	0.617	0.69	0.275	0.306	0.324	0.387	0.288	0.292	0.15	0	0.182	0.166	16	0.400375	0.3555	272.875
RAD54L	0.519	0.531	0.862	0.595	0.866	0.29	0.266	0.876	0.489	0.698	0.233	0.284	0.288	0.353	0	0.176	0.181	16	0.4691875	0.421	204.75
SMARCAL1	0.513	0.508	0.441	0.462	0.429	0.251	0.259	0.49	0.15	0.317	0.101	0.301	0.291	0.348	0	0.325	0.412	16	0.349875	0.3365	205
DNA2	0.506	0.512	0.519	0.522	0.516	0.354	0.33	0.587	0.202	0.361	0.144	0.261	0.253	0.271	0	0.27	0.308	16	0.36975	0.342	183.625
PRIM1	0.5	0.493	0.327	0.422	0.316	0.312	0.249	0.319	0.155	0.201	0	0.417	0.406	0.22	0.18	0.348	0.385	16	0.328125	0.323	228.75
TDP1	0.499	0.502	0.678	0.575	0.676	0.457	0.321	0.283	0.585	0.597	0.458	0.309	0.304	0.255	0	0.142	0.155	16	0.42475	0.4575	283.4375
RRM2B	0.499	0.46	0.251	0.384	0.211	0.209	0.231	0.185	0	0.186	0.109	0.491	0.458	0.306	0.206	0.353	0.269	16	0.3005	0.26	407.3125
FRA1H	0.498	0.5	0.223	0.373	0.21	0.143	0.1	0.142	0	0.164	0.11	0.368	0.363	0.387	0.118	0.49	0.696	16	0.3053125	0.293	754
SSBP1	0.496	0.498	0.371	0.511	0.363	0.538	0.468	0.403	0.122	0.251	0.214	0.316	0.269	0.229	0	0.159	0.17	16	0.336125	0.3395	311.8125
RNF168	0.492	0.486	0.507	0.429	0.499	0.104	0.11	0.383	0.378	0.574	0.134	0.355	0.356	0.461	0	0.286	0.289	16	0.3651875	0.3805	362.1875
TP53I3	0.491	0.474	0.306	0.376	0.251	0.134	0.191	0.214	0.143	0.218	0	0.591	0.585	0.27	0.328	0.346	0.287	16	0.3253125	0.2965	336.0625
REV1	0.49	0.503	0.302	0.526	0.299	0.487	0.458	0.255	0.12	0.172	0.109	0.274	0.267	0.166	0	0.224	0.222	16	0.304625	0.2705	365
MSH5	0.488	0.486	0.556	0.516	0.547	0.168	0.169	0.631	0.229	0.499	0.144	0.293	0.288	0.439	0	0.213	0.231	16	0.3685625	0.366	239.5
MMS22L	0.485	0.495	0.622	0.451	0.618	0.129	0.139	0.584	0.415	0.532	0.112	0.266	0.268	0.327	0	0.342	0.408	16	0.3870625	0.4115	261.375
DBF4B	0.483	0.478	0.234	0.387	0.224	0.151	0.131	0.167	0	0.193	0.159	0.367	0.366	0.372	0.113	0.551	0.614	16	0.311875	0.3	555.3125
RDM1	0.481	0.486	0.426	0.513	0.425	0.382	0.392	0.374	0.129	0.232	0.248	0.284	0.271	0.187	0	0.241	0.241	16	0.332	0.329	235.3125
ASF1B	0.464	0.45	0.334	0.362	0.319	0.134	0.128	0.356	0.144	0.281	0	0.316	0.301	0.357	0.12	0.454	0.484	16	0.31275	0.3265	380.125
WDHD1	0.461	0.457	0.301	0.39	0.284	0.175	0.189	0.299	0.142	0.244	0	0.327	0.317	0.27	0.157	0.455	0.402	16	0.304375	0.3	290.25
TERF2	0.456	0.457	0.53	0.423	0.524	0.131	0.208	0.403	0.435	0.558	0	0.293	0.292	0.392	0.354	0.226	0.237	16	0.3699375	0.3975	245.25
PRIM2	0.455	0.438	0.33	0.374	0.317	0.248	0.229	0.34	0.12	0.214	0.107	0.268	0.239	0.266	0	0.257	0.293	16	0.2809375	0.267	317.8125
LOC401131	0.453	0.38	0.257	0.3	0.228	0.105	0.114	0.237	0.126	0.175	0	0.431	0.366	0.191	0.297	0.334	0.272	16	0.266625	0.2645	526.125
MCM7	0.453	0.442	0.29	0.351	0.275	0.128	0.131	0.29	0.118	0.219	0	0.301	0.294	0.296	0.172	0.483	0.541	16	0.299	0.292	386.375
POLL	0.441	0.455	0.336	0.476	0.331	0.486	0.346	0.24	0.182	0.211	0.223	0.204	0.194	0.136	0	0.184	0.164	16	0.2880625	0.2315	427.625
MCM9	0.438	0.432	0.586	0.408	0.583	0.106	0.125	0.748	0.208	0.454	0.126	0.257	0.251	0.348	0	0.276	0.291	16	0.3523125	0.3195	365.3125

MCM10	0.436	0.43	0.297	0.376	0.286	0.183	0.224	0.333	0.127	0.228	0	0.308	0.296	0.258	0.166	0.425	0.431	16	0.30025	0.2965	285.9375
RPPH1	0.435	0.407	0.36	0.349	0.344	0.228	0.212	0.327	0.139	0.227	0.191	0.311	0.279	0.224	0	0.254	0.287	16	0.285875	0.283	269.4375
TONSL	0.434	0.421	0.494	0.382	0.491	0.117	0.132	0.474	0.321	0.412	0.119	0.287	0.287	0.248	0	0.268	0.318	16	0.3253125	0.3195	336.3125
PARP6	0.427	0.407	0.379	0.367	0.355	0.221	0.206	0.178	0	0.193	0.333	0.392	0.376	0.228	0.116	0.184	0.158	16	0.2825	0.2805	537.0625
GMNC	0.426	0.414	0.315	0.328	0.292	0.105	0.103	0.311	0.123	0.236	0	0.292	0.285	0.307	0.12	0.492	0.525	16	0.292125	0.2995	535.875
DDX11	0.424	0.417	0.326	0.376	0.312	0.191	0.182	0.361	0.114	0.201	0.16	0.225	0.205	0.215	0	0.319	0.332	16	0.2725	0.2685	334.625
CSTF3	0.418	0.397	0.348	0.329	0.33	0.164	0.163	0.361	0.163	0.252	0.116	0.284	0.265	0.266	0	0.307	0.38	16	0.2839375	0.2955	299
PET112	0.418	0.39	0.316	0.315	0.293	0.148	0.148	0.213	0.173	0.326	0.142	0.31	0.273	0.41	0	0.176	0.152	16	0.2626875	0.283	481.3125
TREX1	0.416	0.369	0.328	0.383	0.317	0.32	0.25	0.311	0.203	0.245	0.142	0.223	0.201	0.158	0	0.172	0.213	16	0.2656875	0.2475	390.8125
FRA9E	0.414	0.389	0.271	0.298	0.246	0.109	0.105	0.231	0.148	0.254	0	0.273	0.257	0.347	0.115	0.309	0.376	16	0.258875	0.264	605.75
MCMBP	0.412	0.397	0.321	0.331	0.307	0.127	0.131	0.366	0.142	0.243	0	0.242	0.228	0.237	0.118	0.331	0.348	16	0.2675625	0.275	466.3125
BRIP1	0.407	0.41	0.372	0.447	0.374	0.221	0.184	0.36	0.172	0.4	0.288	0.206	0.206	0.283	0	0.214	0.263	16	0.3004375	0.2855	297.125
POLA2	0.407	0.397	0.338	0.356	0.332	0.279	0.231	0.319	0	0.154	0.191	0.259	0.24	0.179	0.207	0.359	0.346	16	0.287125	0.299	293.75
MBD4	0.405	0.408	0.246	0.489	0.244	0.466	0.316	0.14	0	0.114	0.315	0.294	0.291	0.101	0.122	0.117	0.117	16	0.2615625	0.2685	935.5625
BLM	0.402	0.414	0.653	0.442	0.66	0.183	0.165	0.889	0.2	0.463	0.133	0.215	0.217	0.289	0	0.191	0.243	16	0.3599375	0.266	310.5
ORC1	0.399	0.392	0.292	0.323	0.283	0.108	0.105	0.272	0.197	0.243	0	0.246	0.24	0.239	0.13	0.46	0.464	16	0.2745625	0.259	537.375
THOC1	0.397	0.362	0.263	0.305	0.237	0.193	0.17	0.277	0	0.18	0.121	0.303	0.277	0.228	0.221	0.308	0.267	16	0.2568125	0.265	373.875
CHAF1B	0.395	0.379	0.284	0.317	0.266	0.163	0.165	0.293	0.11	0.208	0	0.242	0.223	0.194	0.179	0.302	0.251	16	0.2481875	0.2465	425.9375
FANCC	0.392	0.366	0.352	0.364	0.339	0.171	0.245	0.439	0.117	0.228	0	0.293	0.274	0.232	0.122	0.253	0.254	16	0.2775625	0.264	378.75
RMI2	0.391	0.395	0.589	0.414	0.594	0.157	0.165	0.827	0.149	0.398	0.119	0.214	0.215	0.267	0	0.2	0.254	16	0.33425	0.2605	346.5625
ERI2	0.391	0.376	0.405	0.367	0.387	0.188	0.257	0.371	0.31	0.373	0	0.236	0.213	0.241	0.131	0.239	0.259	16	0.2965	0.2845	341.375
EXO1	0.389	0.396	0.36	0.515	0.361	0.262	0.234	0.383	0.196	0.323	0.155	0.202	0.203	0.231	0	0.184	0.238	16	0.2895	0.25	334.5
ALKBH3	0.388	0.373	0.314	0.407	0.302	0.351	0.232	0.24	0.138	0.169	0.334	0.252	0.233	0.102	0	0.122	0.126	16	0.2551875	0.246	603
UPF1	0.388	0.358	0.282	0.282	0.256	0.126	0.125	0.328	0.111	0.232	0	0.29	0.262	0.313	0.138	0.278	0.293	16	0.253875	0.28	487.5625
GEN1	0.385	0.39	0.574	0.443	0.574	0.278	0.284	0.733	0.186	0.371	0.157	0.261	0.262	0.195	0	0.163	0.187	16	0.3401875	0.281	303.6875
MIR4511	0.38	0.356	0.327	0.361	0.323	0.205	0.224	0.192	0.184	0.328	0.203	0.255	0.249	0.271	0	0.231	0.165	16	0.265875	0.252	420.625
MIR5089	0.38	0.356	0.327	0.361	0.323	0.205	0.224	0.192	0.184	0.328	0.203	0.255	0.249	0.271	0	0.231	0.165	16	0.265875	0.252	418.625
MIR5093	0.38	0.356	0.327	0.361	0.323	0.205	0.224	0.192	0.184	0.328	0.203	0.255	0.249	0.271	0	0.231	0.165	16	0.265875	0.252	417.625
MIR5091	0.38	0.356	0.327	0.361	0.323	0.205	0.224	0.192	0.184	0.328	0.203	0.255	0.249	0.271	0	0.231	0.165	16	0.265875	0.252	419.625
MIR5092	0.38	0.356	0.327	0.361	0.323	0.205	0.224	0.192	0.184	0.328	0.203	0.255	0.249	0.271	0	0.231	0.165	16	0.265875	0.252	421.625

MIR5087	0.38	0.356	0.327	0.361	0.323	0.205	0.224	0.192	0.184	0.328	0.203	0.255	0.249	0.271	0	0.231	0.165	16	0.265875	0.252	423.625
MIR5088	0.38	0.356	0.327	0.361	0.323	0.205	0.224	0.192	0.184	0.328	0.203	0.255	0.249	0.271	0	0.231	0.165	16	0.265875	0.252	424.625
MIR5090	0.38	0.356	0.327	0.361	0.323	0.205	0.224	0.192	0.184	0.328	0.203	0.255	0.249	0.271	0	0.231	0.165	16	0.265875	0.252	425.625
MIR5094	0.38	0.356	0.327	0.361	0.323	0.205	0.224	0.192	0.184	0.328	0.203	0.255	0.249	0.271	0	0.231	0.165	16	0.265875	0.252	422.625
TOP3B	0.38	0.369	0.526	0.367	0.522	0.138	0.174	0.74	0.157	0.336	0	0.36	0.363	0.185	0.178	0.218	0.179	16	0.3245	0.348	350.0625
MCM4	0.376	0.369	0.275	0.314	0.266	0.173	0.143	0.275	0.152	0.23	0	0.244	0.238	0.213	0.172	0.392	0.42	16	0.26575	0.255	385.5
CTC1	0.376	0.378	0.329	0.338	0.328	0.149	0.192	0.342	0.187	0.273	0	0.214	0.208	0.244	0.317	0.249	0.31	16	0.277125	0.2915	338.375
APTX	0.373	0.377	0.44	0.495	0.449	0.418	0.214	0.157	0.229	0.218	0.725	0.269	0.269	0.111	0	0.104	0.112	16	0.31	0.269	737.25
NDUFA9P1	0.368	0.346	0.297	0.301	0.284	0.192	0.166	0.315	0	0.205	0.105	0.237	0.222	0.202	0.183	0.13	0.112	16	0.2290625	0.2135	626.6875
NIPBL	0.366	0.357	0.368	0.326	0.345	0.136	0.13	0.405	0.134	0.284	0	0.23	0.227	0.304	0.12	0.234	0.247	16	0.2633125	0.2655	484.1875
STAG3L3	0.363	0.367	0.555	0.378	0.552	0.102	0.101	0.783	0.171	0.417	0.134	0.189	0.186	0.314	0	0.239	0.273	16	0.32025	0.2935	528
STAG3L1	0.363	0.367	0.555	0.378	0.552	0.102	0.101	0.783	0.171	0.417	0.134	0.189	0.186	0.314	0	0.239	0.273	16	0.32025	0.2935	531
STAG3L2	0.363	0.367	0.555	0.378	0.552	0.102	0.101	0.783	0.171	0.417	0.134	0.189	0.186	0.314	0	0.239	0.273	16	0.32025	0.2935	530
STAG3L4	0.363	0.367	0.555	0.378	0.552	0.102	0.101	0.783	0.171	0.417	0.134	0.189	0.186	0.314	0	0.239	0.273	16	0.32025	0.2935	529
MRPL43	0.361	0.325	0.262	0.274	0.233	0.196	0.159	0.275	0.115	0.157	0	0.224	0.184	0.15	0.139	0.286	0.252	16	0.2245	0.2285	538.6875
DCP1B	0.36	0.333	0.392	0.32	0.38	0.147	0.163	0.377	0.281	0.328	0.111	0.197	0.17	0.192	0	0.199	0.229	16	0.2611875	0.255	451.625
RAD54B	0.356	0.365	0.683	0.405	0.691	0.112	0.124	0.917	0.215	0.505	0.164	0.169	0.175	0.289	0	0.143	0.146	16	0.3411875	0.252	586.9375
TOP3A	0.355	0.36	0.606	0.39	0.611	0.154	0.146	0.851	0.166	0.42	0.125	0.177	0.175	0.241	0	0.164	0.195	16	0.321	0.218	464.75
MCM2	0.355	0.345	0.299	0.287	0.283	0.1	0.103	0.294	0.14	0.231	0	0.235	0.231	0.212	0.189	0.346	0.371	16	0.2513125	0.259	571.25
ATRX	0.353	0.356	0.622	0.373	0.624	0.132	0.143	0.771	0.277	0.477	0	0.177	0.176	0.21	0.114	0.122	0.125	16	0.31575	0.2435	683.5
PARN	0.352	0.324	0.232	0.289	0.214	0.3	0.229	0.23	0	0.142	0.134	0.241	0.205	0.14	0.109	0.21	0.234	16	0.2240625	0.2295	616.4375
H2AFY	0.352	0.339	0.366	0.271	0.355	0.137	0.111	0.244	0	0.217	0.258	0.248	0.236	0.166	0.133	0.176	0.163	16	0.23575	0.24	626.625
DDX21	0.346	0.336	0.541	0.383	0.542	0.165	0.124	0.314	0.582	0.521	0.245	0.201	0.181	0.189	0	0.139	0.156	16	0.3103125	0.2795	509.25
SUPV3L1	0.346	0.329	0.359	0.298	0.342	0.171	0.154	0.426	0.128	0.235	0	0.2	0.179	0.185	0.12	0.211	0.251	16	0.245875	0.223	520.625
POLR1A	0.345	0.332	0.331	0.289	0.313	0.126	0.126	0.435	0.115	0.239	0	0.239	0.228	0.175	0.104	0.221	0.152	16	0.235625	0.2335	640.25
TOP1	0.342	0.332	0.449	0.319	0.444	0.207	0.201	0.354	0.215	0.293	0.216	0.275	0.264	0.15	0	0.166	0.153	16	0.27375	0.2695	391.0625
COA5	0.342	0.304	0.282	0.293	0.268	0.181	0.204	0.33	0.128	0.186	0	0.209	0.164	0.13	0.107	0.186	0.179	16	0.2183125	0.195	645
MTERFD3	0.341	0.306	0.268	0.252	0.25	0.117	0.123	0.285	0.132	0.199	0	0.242	0.215	0.17	0.162	0.261	0.25	16	0.2233125	0.246	587
OR5G1P	0.338	0.33	0.227	0.311	0.215	0.254	0.2	0.218	0	0.129	0.146	0.227	0.223	0.186	0.106	0.178	0.231	16	0.2199375	0.2205	654.1875
SUPT16H	0.338	0.322	0.291	0.272	0.284	0.14	0.147	0.276	0	0.187	0.134	0.229	0.223	0.164	0.116	0.202	0.191	16	0.21975	0.2125	605.125

TBPL1	0.336	0.319	0.284	0.275	0.271	0.121	0.156	0.31	0.169	0.236	0	0.266	0.25	0.177	0.243	0.251	0.201	16	0.2415625	0.2505	461.3125
SSRP1	0.334	0.326	0.246	0.28	0.236	0.163	0.163	0.235	0	0.136	0.114	0.201	0.193	0.122	0.111	0.188	0.172	16	0.20125	0.1905	743.125
DUX4L14	0.333	0.312	0.298	0.287	0.279	0.162	0.187	0.314	0.164	0.221	0	0.164	0.145	0.148	0.167	0.192	0.17	16	0.2214375	0.1895	616.375
DHX35	0.333	0.304	0.342	0.285	0.338	0.14	0.166	0.284	0.305	0.323	0	0.185	0.162	0.147	0.148	0.188	0.191	16	0.2400625	0.2375	576.0625
MGME1	0.331	0.327	0.29	0.324	0.273	0.332	0.241	0.286	0.173	0.245	0.102	0.181	0.153	0.157	0	0.129	0.166	16	0.231875	0.243	620.6875
DHX36	0.326	0.298	0.294	0.261	0.283	0.116	0.133	0.27	0.171	0.276	0	0.187	0.171	0.262	0.183	0.149	0.14	16	0.22	0.224	699
TERF2IP	0.323	0.321	0.425	0.318	0.423	0.112	0.205	0.342	0.381	0.436	0	0.188	0.186	0.228	0.32	0.142	0.149	16	0.2811875	0.319	551.25
IK	0.318	0.29	0.16	0.26	0.142	0.314	0.194	0.139	0	0.11	0.113	0.191	0.178	0.171	0.145	0.167	0.144	16	0.18975	0.169	1089.125
CSTF1	0.315	0.306	0.263	0.275	0.255	0.127	0.137	0.239	0.148	0.302	0.15	0.146	0.137	0.21	0	0.191	0.157	16	0.209875	0.2005	744.0625
POLG2	0.315	0.303	0.265	0.3	0.246	0.294	0.22	0.298	0.105	0.138	0.124	0.161	0.136	0	0.104	0.14	0.128	16	0.2048125	0.1905	852.4375
EIF3EP1	0.312	0.291	0.319	0.289	0.31	0.211	0.165	0.325	0.146	0.221	0.181	0.198	0.184	0.163	0.183	0.133	0	16	0.2269375	0.2045	553.75
SUGP1	0.309	0.288	0.269	0.233	0.244	0.125	0.118	0.235	0.177	0.163	0.108	0.462	0.444	0	0.203	0.192	0.12	16	0.230625	0.218	672.625
OBFC1	0.308	0.312	0.271	0.284	0.271	0.128	0.156	0.289	0.162	0.23	0	0.181	0.178	0.22	0.26	0.246	0.281	16	0.2360625	0.253	513.5625
FRA18C	0.299	0.296	0.286	0.255	0.268	0.14	0.105	0.292	0.136	0.225	0	0.162	0.157	0.237	0.103	0.223	0.248	16	0.2145	0.231	762.625
BAZ1A	0.292	0.281	0.395	0.276	0.39	0.107	0.111	0.24	0.469	0.423	0	0.136	0.124	0.127	0.155	0.167	0.128	16	0.2388125	0.2035	942.625
POLR2A	0.29	0.278	0.281	0.268	0.273	0.182	0.211	0.344	0.126	0.244	0	0.159	0.146	0.155	0.119	0.167	0.137	16	0.21125	0.1965	737.3125
HIST1H2BA	0.288	0.28	0.311	0.237	0.308	0.121	0.106	0.302	0	0.174	0.183	0.19	0.185	0.134	0.138	0.205	0.196	16	0.209875	0.193	720.625
ACTR5	0.286	0.281	0.298	0.263	0.292	0.115	0.138	0.288	0.169	0.248	0	0.217	0.203	0.153	0.141	0.251	0.223	16	0.222875	0.2355	591.875
TRIT1	0.278	0.256	0.201	0.224	0.185	0.161	0.168	0.26	0	0.11	0.108	0.203	0.174	0.119	0.11	0.11	0.112	16	0.1736875	0.171	1049
DUX4L15	0.276	0.255	0.23	0.22	0.209	0.123	0.133	0.267	0.106	0.157	0	0.12	0.103	0.112	0.138	0.154	0.127	16	0.170625	0.146	1163.8125
SUPT5H	0.274	0.259	0.239	0.232	0.232	0.135	0.142	0.295	0.106	0.215	0	0.138	0.121	0.131	0.114	0.196	0.189	16	0.188625	0.1925	919
H2AFB2	0.27	0.265	0.25	0.306	0.241	0.268	0.319	0.255	0.105	0.156	0.121	0.15	0.146	0.103	0	0.122	0.103	16	0.19875	0.1985	917.375
SUPT4H1	0.27	0.25	0.239	0.224	0.227	0.132	0.137	0.284	0.11	0.218	0	0.137	0.119	0.133	0.103	0.167	0.141	16	0.1806875	0.154	1029.5625
PAPD4	0.267	0.236	0.248	0.202	0.235	0.118	0.12	0.257	0.159	0.185	0	0.235	0.205	0.102	0.169	0.206	0.146	16	0.193125	0.2035	787.375
POLR1C	0.263	0.243	0.269	0.228	0.26	0.143	0.136	0.364	0.101	0.168	0	0.157	0.127	0.103	0.105	0.159	0.151	16	0.1860625	0.158	972.875
TNKS	0.26	0.258	0.305	0.223	0.305	0.116	0.117	0.179	0.125	0.183	0.193	0.195	0.188	0	0.263	0.115	0.137	16	0.197625	0.1905	937.8125
SCARNA23	0.257	0.245	0.295	0.214	0.285	0	0.101	0.297	0.135	0.236	0.106	0.147	0.125	0.142	0.182	0.149	0.161	16	0.1923125	0.1715	914.1875
TPP1	0.245	0.231	0.201	0.193	0.189	0.136	0.123	0.211	0.129	0.155	0	0.129	0.115	0.121	0.24	0.101	0.116	16	0.1646875	0.1455	1280.375
GTPBP10	0.244	0.222	0.223	0.208	0.211	0.136	0.172	0.231	0.137	0.168	0	0.225	0.202	0.12	0.172	0.182	0.157	16	0.188125	0.192	770.75
USB1	0.243	0.216	0.206	0.193	0.193	0.154	0.142	0.201	0.165	0.166	0	0.139	0.126	0.108	0.103	0.128	0.141	16	0.164	0.1595	1211.25

FRA13A	0.233	0.219	0.183	0.178	0.167	0.11	0.101	0.197	0	0.113	0.102	0.18	0.172	0.142	0.128	0.134	0.11	16	0.1543125	0.1545	1316.125
FRA2H	0.218	0.21	0.208	0.225	0.206	0.114	0.113	0.154	0.137	0.169	0.17	0.124	0.126	0.13	0	0.11	0.121	16	0.1584375	0.1455	1417.875
RAD1	0.667	0.68	0.347	0.586	0.337	0.379	0.283	0.268	0	0.24	0.194	0.424	0.42	0.469	0	0.504	0.638	15	0.429066667	0.42	135.8
SMC1A	0.601	0.596	0.511	0.477	0.496	0	0	0.509	0.191	0.5	0.139	0.409	0.411	0.662	0.143	0.525	0.62	15	0.452666667	0.5	159.8666667
RPA2	0.591	0.602	0.545	0.575	0.543	0.3	0.38	0.553	0.254	0.449	0	0.349	0.35	0.408	0	0.446	0.544	15	0.459266667	0.449	83.13333333
RPA1	0.588	0.601	0.501	0.593	0.501	0.341	0.432	0.584	0.174	0.382	0	0.351	0.355	0.383	0	0.406	0.525	15	0.4478	0.432	97.6
C19ORF40	0.574	0.57	0.412	0.574	0.408	0.289	0.465	0.494	0.102	0.314	0	0.385	0.385	0.408	0	0.471	0.566	15	0.4278	0.412	140.1333333
ATM	0.56	0.554	0.353	0.399	0.33	0	0	0.132	0.172	0.517	0.102	0.439	0.444	0.837	0.122	0.329	0.346	15	0.375733333	0.353	581.9333333
HUS1B	0.556	0.555	0.293	0.448	0.279	0.237	0.17	0.324	0	0.18	0.122	0.399	0.4	0.4	0	0.497	0.623	15	0.365533333	0.399	221
RNF8	0.541	0.539	0.525	0.459	0.516	0	0.102	0.465	0.293	0.555	0.139	0.375	0.376	0.52	0	0.355	0.374	15	0.408933333	0.459	233.8666667
RIF1	0.536	0.53	0.477	0.442	0.47	0	0	0.447	0.256	0.509	0.15	0.338	0.338	0.48	0.19	0.391	0.462	15	0.401066667	0.447	146.2666667
FANCM	0.531	0.529	0.489	0.54	0.482	0.255	0.346	0.611	0.144	0.316	0	0.331	0.326	0.332	0	0.401	0.46	15	0.4062	0.401	135.6
RPAP1	0.513	0.518	0.326	0.621	0.324	0.588	0.779	0.281	0.147	0.24	0	0.304	0.295	0.2	0	0.211	0.235	15	0.372133333	0.304	264.3333333
COA1	0.511	0.52	0.47	0.46	0.456	0.156	0.186	0.408	0.229	0.373	0	0.308	0.309	0.345	0	0.385	0.468	15	0.372266667	0.385	178.6666667
EXO5	0.505	0.522	0.496	0.505	0.503	0.311	0.376	0.588	0.113	0.323	0	0.317	0.324	0.299	0	0.321	0.455	15	0.3972	0.376	161.7333333
POLE	0.48	0.484	0.346	0.551	0.334	0.49	0.474	0.339	0	0.192	0.189	0.246	0.237	0.185	0	0.263	0.27	15	0.338666667	0.334	253.5333333
FRA2G	0.48	0.484	0.208	0.358	0.198	0.145	0	0.12	0.103	0.159	0	0.344	0.342	0.382	0.12	0.484	0.696	15	0.3082	0.342	809.8666667
POLK	0.479	0.496	0.28	0.512	0.278	0.527	0.462	0.249	0	0.144	0.113	0.241	0.233	0.146	0	0.201	0.218	15	0.305266667	0.249	419.2666667
ANKRD32	0.472	0.474	0.407	0.438	0.397	0.174	0.198	0.431	0.164	0.324	0	0.256	0.252	0.318	0	0.322	0.328	15	0.330333333	0.324	226
RBBP8	0.472	0.47	0.455	0.416	0.451	0	0	0.379	0.306	0.557	0.186	0.268	0.271	0.447	0.16	0.331	0.366	15	0.369	0.379	196.0666667
MAD2L2	0.466	0.467	0.274	0.446	0.267	0.369	0.35	0.258	0	0.15	0.108	0.28	0.268	0.18	0	0.32	0.326	15	0.301933333	0.28	327.9333333
RPA3	0.458	0.466	0.405	0.507	0.407	0.365	0.519	0.519	0.115	0.279	0	0.251	0.247	0.252	0	0.299	0.34	15	0.361933333	0.365	214.6
DCLRE1C	0.457	0.455	0.648	0.437	0.637	0.119	0.114	0.352	0.667	0.748	0	0.284	0.287	0.405	0	0.22	0.233	15	0.4042	0.405	356
HNRNPUL1	0.455	0.434	0.293	0.331	0.284	0	0.103	0.252	0.152	0.281	0	0.489	0.491	0.364	0.452	0.344	0.296	15	0.334733333	0.331	330.6666667
FANCI	0.451	0.45	0.386	0.412	0.377	0.149	0.172	0.453	0.132	0.307	0	0.324	0.323	0.392	0	0.315	0.343	15	0.3324	0.343	241.7333333
CDC5L	0.44	0.418	0.287	0.352	0.274	0.173	0.179	0.296	0	0.182	0.111	0.307	0.288	0.252	0	0.383	0.498	15	0.296	0.288	311.6666667
POLI	0.437	0.454	0.252	0.481	0.249	0.522	0.433	0.197	0	0.112	0.135	0.215	0.205	0.103	0	0.178	0.163	15	0.275733333	0.215	633.9333333
GADD45A	0.432	0.401	0.171	0.333	0.153	0.165	0.168	0.127	0	0.12	0	0.399	0.369	0.207	0.27	0.454	0.364	15	0.275533333	0.27	805.3333333
RPAIN	0.418	0.423	0.378	0.425	0.379	0.255	0.374	0.486	0.107	0.294	0	0.232	0.233	0.312	0	0.277	0.37	15	0.330866667	0.37	249.4666667
POLD1	0.417	0.424	0.291	0.48	0.29	0.415	0.293	0.213	0	0.162	0.324	0.191	0.185	0.139	0	0.232	0.226	15	0.285466667	0.29	449.9333333

FAN1	0.415	0.416	0.358	0.508	0.355	0.271	0.347	0.415	0.133	0.287	0	0.266	0.267	0.268	0	0.224	0.26	15	0.319333333	0.287	254.4666667
CDT1	0.415	0.416	0.185	0.325	0.175	0.109	0.111	0.163	0	0.127	0	0.249	0.247	0.265	0.124	0.52	0.5	15	0.262066667	0.247	843.9333333
PARP11	0.414	0.394	0.371	0.355	0.337	0.186	0.209	0.208	0	0.215	0.251	0.394	0.383	0.271	0	0.183	0.161	15	0.2888	0.271	407.8666667
POLN	0.403	0.412	0.311	0.399	0.309	0.246	0.229	0.284	0.118	0.196	0	0.213	0.207	0.187	0	0.236	0.223	15	0.264866667	0.236	389.5333333
PARP9	0.4	0.392	0.42	0.307	0.413	0.137	0.105	0.137	0	0.3	0.361	0.334	0.334	0.38	0	0.133	0.14	15	0.2862	0.334	826.3333333
ORC5	0.399	0.392	0.377	0.332	0.361	0.121	0.121	0.327	0.317	0.31	0	0.246	0.235	0.222	0	0.415	0.486	15	0.310733333	0.327	366.1333333
MCM6	0.395	0.387	0.338	0.333	0.328	0.151	0.149	0.371	0.159	0.259	0	0.215	0.203	0.226	0	0.332	0.38	15	0.281733333	0.328	348.7333333
PURG	0.391	0.401	0.544	0.395	0.549	0.212	0.186	0.706	0.197	0.396	0	0.207	0.206	0.248	0	0.203	0.28	15	0.3414	0.28	297.6
PNKP	0.387	0.394	0.465	0.527	0.478	0.458	0.252	0.119	0.241	0.225	0.784	0.287	0.29	0.103	0	0	0.11	15	0.341333333	0.29	740.8666667
DCLRE1A	0.385	0.381	0.272	0.482	0.267	0.362	0.674	0.271	0.124	0.253	0	0.248	0.25	0.21	0	0.178	0.171	15	0.301866667	0.267	399.9333333
UBL4B	0.383	0.382	0.265	0.333	0.256	0.214	0.233	0.231	0	0.211	0	0.284	0.265	0.219	0.163	0.237	0.255	15	0.262066667	0.255	412.8
ORC4	0.375	0.368	0.404	0.322	0.39	0.104	0.108	0.288	0.448	0.384	0	0.21	0.202	0.207	0	0.386	0.423	15	0.307933333	0.368	486.6666667
CCNO	0.373	0.383	0.236	0.421	0.232	0.471	0.358	0.218	0	0.116	0.161	0.175	0.166	0.123	0	0.236	0.183	15	0.2568	0.232	598.6
FANCA	0.372	0.362	0.411	0.378	0.401	0.146	0.191	0.512	0.146	0.242	0	0.271	0.264	0.244	0	0.251	0.28	15	0.298066667	0.271	308
PARP15	0.371	0.35	0.34	0.296	0.321	0.154	0.159	0.185	0	0.206	0.24	0.322	0.313	0.252	0	0.145	0.127	15	0.252066667	0.252	611.4
MIR1206	0.369	0.351	0.152	0.334	0.144	0.258	0.26	0.1	0	0	0.153	0.32	0.32	0.172	0.164	0.283	0.347	15	0.248466667	0.26	1006.933333
ACTR8	0.367	0.355	0.435	0.316	0.421	0	0	0.414	0.249	0.357	0.11	0.278	0.261	0.224	0.148	0.228	0.238	15	0.2934	0.278	345.5333333
GINS2	0.366	0.352	0.303	0.312	0.292	0.148	0.15	0.272	0.143	0.238	0	0.234	0.225	0.248	0	0.315	0.312	15	0.260666667	0.272	393.8
SMARCA5	0.363	0.355	0.327	0.289	0.318	0.101	0	0.24	0.227	0.315	0	0.208	0.2	0.259	0.129	0.192	0.169	15	0.246133333	0.24	643.2
C10ORF2	0.36	0.347	0.357	0.323	0.346	0.249	0.213	0.43	0.126	0.209	0	0.183	0.156	0.156	0	0.147	0.17	15	0.251466667	0.213	553.5333333
WRAP53	0.359	0.347	0.308	0.299	0.303	0	0	0.362	0.113	0.195	0.103	0.438	0.437	0.134	0.365	0.197	0.12	15	0.272	0.303	478.2666667
MCM8	0.358	0.353	0.56	0.349	0.561	0	0.117	0.757	0.158	0.413	0.113	0.198	0.196	0.308	0	0.198	0.215	15	0.3236	0.308	427.4
HMGN1	0.355	0.338	0.283	0.27	0.269	0.149	0.126	0.202	0	0.176	0.154	0.222	0.205	0.199	0	0.173	0.146	15	0.2178	0.202	673.5333333
RAD21	0.355	0.349	0.387	0.323	0.378	0.103	0	0.525	0.148	0.28	0.134	0.205	0.197	0.256	0	0.299	0.319	15	0.283866667	0.299	442.4666667
PSME4	0.353	0.344	0.207	0.312	0.202	0.369	0.222	0.15	0	0.108	0.226	0.264	0.229	0.145	0	0.135	0.123	15	0.225933333	0.222	877.2
TERF1	0.35	0.351	0.396	0.312	0.391	0	0.14	0.326	0.308	0.38	0	0.23	0.228	0.255	0.368	0.206	0.208	15	0.2966	0.312	344.6666667
SMARCAD1	0.349	0.322	0.366	0.299	0.346	0.141	0.181	0.46	0.155	0.262	0	0.218	0.212	0.201	0	0.23	0.243	15	0.265666667	0.243	402.6
POLE3	0.345	0.346	0.56	0.365	0.552	0.146	0.14	0.289	0.782	0.63	0	0.148	0.143	0.168	0	0.179	0.189	15	0.332133333	0.289	585.2666667
RNASEH2C	0.344	0.334	0.252	0.293	0.233	0.257	0.191	0.293	0	0.166	0	0.182	0.162	0.174	0.137	0.201	0.174	15	0.2262	0.201	606.7333333
TP53I13	0.344	0.288	0.169	0.211	0.136	0.101	0	0.194	0	0.114	0.109	0.355	0.284	0.16	0.179	0.246	0.189	15	0.205266667	0.189	913.9333333

FANCG	0.343	0.337	0.417	0.363	0.41	0.137	0.188	0.504	0.173	0.234	0	0.263	0.259	0.222	0	0.239	0.267	15	0.2904	0.263	338.5333333
GTF2F2P1	0.339	0.337	0.271	0.316	0.264	0.313	0.194	0.296	0	0.191	0.155	0.226	0.219	0.178	0	0.18	0.196	15	0.245	0.226	453.0666667
POLR3GL	0.338	0.313	0.229	0.282	0.211	0.144	0.213	0.229	0	0.205	0	0.277	0.263	0.259	0.256	0.219	0.123	15	0.2374	0.229	555.5333333
SSBP2	0.336	0.319	0.277	0.272	0.266	0.122	0.123	0.323	0	0.207	0	0.368	0.369	0.217	0.166	0.233	0.213	15	0.254066667	0.266	510.8
RMI1	0.335	0.348	0.613	0.374	0.623	0.132	0.127	0.874	0.177	0.421	0	0.155	0.159	0.236	0	0.158	0.221	15	0.3302	0.236	566.7333333
FEN1	0.333	0.348	0.274	0.383	0.274	0.377	0.275	0.239	0.102	0.153	0.196	0.12	0.116	0	0	0.144	0.102	15	0.229066667	0.239	928.1333333
CIZ1	0.331	0.304	0.199	0.261	0.185	0.13	0.13	0.245	0	0.133	0	0.219	0.2	0.139	0.184	0.337	0.326	15	0.221533333	0.2	659.8666667
C17ORF70	0.33	0.325	0.265	0.34	0.265	0.189	0.286	0.352	0	0.19	0	0.221	0.213	0.183	0.102	0.201	0.209	15	0.244733333	0.221	549.8
PAPD7	0.326	0.303	0.327	0.266	0.319	0.178	0.177	0.232	0	0.156	0.237	0.245	0.23	0.117	0	0.169	0.187	15	0.231266667	0.232	563.0666667
ZMAT3	0.326	0.297	0.157	0.236	0.133	0.128	0.14	0.152	0	0.105	0	0.355	0.34	0.122	0.36	0.326	0.182	15	0.223933333	0.182	963.6666667
TMEM189-UBE2V1	0.326	0.311	0.223	0.271	0.211	0.144	0.128	0.272	0	0.146	0	0.219	0.207	0.163	0.161	0.216	0.221	15	0.2146	0.216	636.8666667
TRNT1	0.325	0.299	0.266	0.246	0.253	0.141	0.131	0.3	0.117	0.211	0	0.204	0.185	0.228	0	0.176	0.195	15	0.218466667	0.211	614.0666667
POLE2	0.325	0.306	0.243	0.279	0.231	0.2	0.17	0.277	0	0.145	0	0.193	0.171	0.137	0.116	0.212	0.215	15	0.214666667	0.212	665.2
MED30	0.323	0.282	0.195	0.227	0.172	0.11	0.118	0.232	0	0.166	0	0.199	0.172	0.179	0.125	0.21	0.154	15	0.190933333	0.179	913.2666667
DHX9	0.322	0.307	0.409	0.307	0.4	0.208	0.187	0.39	0.316	0.361	0	0.161	0.144	0.139	0	0.187	0.155	15	0.2662	0.307	550.6
ADIRF	0.321	0.268	0.165	0.274	0.15	0.127	0.168	0.152	0	0	0.125	0.253	0.233	0.112	0.161	0.279	0.235	15	0.201533333	0.168	920
IFIT1P1	0.32	0.288	0.262	0.248	0.252	0.169	0.15	0.309	0.102	0.174	0	0.192	0.17	0.154	0.149	0.104	0	15	0.202866667	0.174	797.4
RPS3	0.32	0.298	0.208	0.32	0.195	0.362	0.297	0.163	0.161	0.154	0.114	0.244	0.214	0	0	0.175	0.148	15	0.224866667	0.208	727.1333333
PRDM9	0.319	0.298	0.368	0.282	0.353	0.131	0.13	0.502	0.116	0.271	0	0.169	0.161	0.194	0	0.144	0.165	15	0.2402	0.194	680.0666667
RNASEH2A	0.315	0.296	0.224	0.257	0.207	0.206	0.156	0.263	0	0.136	0	0.175	0.146	0.143	0.12	0.194	0.169	15	0.200466667	0.194	782.1333333
SDE2	0.314	0.312	0.249	0.258	0.237	0.1	0	0.251	0.126	0.21	0	0.205	0.206	0.224	0.135	0.243	0.271	15	0.222733333	0.237	665.6
SMARCA1	0.308	0.282	0.301	0.24	0.294	0.142	0.132	0.273	0	0.176	0.147	0.182	0.163	0.119	0	0.143	0.123	15	0.201666667	0.176	795.1333333
HIST1H1T	0.307	0.287	0.295	0.241	0.278	0.136	0.112	0.333	0	0.196	0.11	0.202	0.189	0.176	0	0.2	0.208	15	0.218	0.202	634.6666667
PARP8	0.305	0.285	0.279	0.254	0.253	0.189	0.197	0.233	0	0.145	0.128	0.214	0.205	0.164	0	0.119	0.107	15	0.205133333	0.205	789.2666667
SMC4	0.305	0.287	0.287	0.239	0.279	0.109	0	0.263	0	0.174	0.257	0.225	0.2	0.199	0.206	0.29	0.284	15	0.240266667	0.257	527.9333333
CYP51P2	0.304	0.283	0.268	0.255	0.251	0.193	0.168	0.313	0	0.158	0.108	0.176	0.161	0.136	0.156	0.106	0	15	0.2024	0.176	803.6666667
RPL21P1	0.304	0.295	0.298	0.231	0.286	0	0	0.311	0.116	0.209	0.105	0.222	0.216	0.19	0.146	0.196	0.276	15	0.226733333	0.222	537.6666667
DHX15	0.303	0.277	0.309	0.263	0.295	0.175	0.192	0.392	0.136	0.239	0	0.175	0.151	0.144	0	0.175	0.164	15	0.226	0.192	612
NCAPG	0.3	0.294	0.302	0.292	0.297	0.23	0.169	0.167	0	0.102	0.383	0.238	0.233	0.124	0	0.188	0.193	15	0.234133333	0.233	709.0666667
MTERFD1	0.3	0.256	0.198	0.21	0.177	0.119	0.106	0.25	0	0.137	0	0.173	0.142	0.123	0.207	0.281	0.227	15	0.193733333	0.198	902.8666667

DHX8	0.299	0.283	0.285	0.262	0.278	0.159	0.168	0.372	0.11	0.196	0	0.165	0.144	0.152	0	0.189	0.192	15	0.216933333	0.192	650.8
MAU2	0.298	0.292	0.339	0.264	0.328	0	0.112	0.454	0.107	0.241	0	0.182	0.178	0.227	0.101	0.263	0.295	15	0.2454	0.263	636.8
NCAPD2	0.297	0.297	0.353	0.288	0.354	0.232	0.152	0.156	0	0.127	0.445	0.239	0.237	0.106	0	0.155	0.175	15	0.240866667	0.237	770.2666667
MAP2K4P1	0.297	0.271	0.183	0.285	0.168	0.187	0.192	0.234	0	0.104	0	0.218	0.2	0.1	0.277	0.189	0.153	15	0.203866667	0.192	788.9333333
PURA	0.295	0.265	0.312	0.239	0.295	0.126	0.119	0.4	0	0.186	0	0.189	0.172	0.146	0.116	0.219	0.186	15	0.217666667	0.189	741.0666667
WRNIP1	0.294	0.291	0.269	0.271	0.267	0.128	0.12	0.311	0.114	0.2	0	0.253	0.25	0.188	0	0.17	0.175	15	0.220066667	0.25	625.7333333
MSH4	0.294	0.297	0.541	0.394	0.544	0	0.102	0.79	0.169	0.388	0.129	0.138	0.131	0.205	0	0.114	0.146	15	0.292133333	0.205	827.2666667
APPBP2	0.293	0.278	0.395	0.239	0.387	0.106	0	0.33	0.115	0.259	0.228	0.21	0.193	0.199	0	0.133	0.151	15	0.2344	0.228	676.4
FAM162B	0.293	0.257	0.191	0.227	0.185	0.135	0.171	0.134	0.104	0.104	0	0.201	0.17	0	0.275	0.131	0.107	15	0.179	0.171	1228.866667
HIST2H2AA4	0.293	0.269	0.184	0.204	0.171	0.146	0.135	0.223	0	0.144	0	0.164	0.131	0.166	0.143	0.158	0.11	15	0.176066667	0.164	1060.466667
NELFB	0.293	0.278	0.193	0.251	0.189	0.106	0	0.16	0	0.254	0.208	0.186	0.18	0.19	0.117	0.188	0.16	15	0.196866667	0.189	982.0666667
UBE2T	0.292	0.284	0.193	0.265	0.187	0.14	0.127	0.194	0	0.271	0.104	0.19	0.184	0.243	0	0.161	0.155	15	0.199333333	0.19	835
HIST1H4I	0.291	0.275	0.303	0.239	0.293	0.125	0.111	0.3	0.185	0.229	0	0.184	0.17	0	0.101	0.129	0.107	15	0.2028	0.185	959.6
HIST1H2BG	0.289	0.263	0.238	0.218	0.229	0.109	0.106	0.252	0.134	0.179	0	0.165	0.149	0.124	0	0.179	0.146	15	0.185333333	0.179	942.8666667
UPF2	0.289	0.261	0.214	0.21	0.195	0.122	0.12	0.274	0	0.148	0	0.195	0.164	0.186	0.111	0.213	0.234	15	0.195733333	0.195	840.2666667
TCEA2	0.286	0.264	0.258	0.257	0.244	0.202	0.21	0.313	0.107	0.192	0	0.229	0.214	0.136	0	0.128	0.117	15	0.210466667	0.214	705.1333333
CENPB	0.283	0.269	0.333	0.247	0.319	0.235	0.173	0.245	0.103	0.175	0.238	0.183	0.172	0	0	0.172	0.196	15	0.222866667	0.235	596.6666667
GTSF1L	0.281	0.259	0.242	0.231	0.228	0.115	0.111	0.287	0	0.163	0.108	0.17	0.155	0.166	0	0.202	0.178	15	0.193066667	0.178	830.8666667
TINF2	0.278	0.278	0.343	0.25	0.34	0	0.11	0.317	0.255	0.307	0	0.154	0.149	0.178	0.353	0.142	0.149	15	0.2402	0.255	725.1333333
HIST2H2AC	0.277	0.268	0.226	0.231	0.22	0.105	0.123	0.246	0.116	0.216	0	0.183	0.172	0.179	0	0.191	0.151	15	0.1936	0.191	827.4666667
HAT1	0.277	0.258	0.186	0.212	0.176	0.143	0.112	0.188	0	0.13	0	0.209	0.194	0.125	0.124	0.164	0.116	15	0.174266667	0.176	1080.8
EIF5A2	0.277	0.244	0.21	0.209	0.197	0.123	0.123	0.195	0	0.128	0	0.224	0.199	0.109	0.174	0.205	0.168	15	0.185666667	0.197	904.3333333
HIST4H4	0.277	0.268	0.182	0.217	0.172	0.139	0.11	0.216	0	0.133	0	0.178	0.167	0.123	0.166	0.185	0.141	15	0.178266667	0.172	992.4
EIF1	0.274	0.245	0.206	0.199	0.192	0.113	0.105	0.289	0	0.159	0.105	0.15	0.119	0.136	0	0.193	0.19	15	0.178333333	0.19	1035.8
H3F3B	0.274	0.252	0.186	0.196	0.165	0.111	0.101	0.241	0	0.12	0	0.161	0.143	0.109	0.115	0.199	0.186	15	0.1706	0.165	1174.733333
POT1	0.274	0.281	0.28	0.245	0.28	0	0.102	0.26	0.209	0.256	0	0.162	0.162	0.179	0.347	0.166	0.202	15	0.227	0.245	693.6666667
QSOX1	0.271	0.246	0.193	0.199	0.178	0.141	0.132	0.252	0.101	0.141	0	0.188	0.138	0.125	0	0.149	0.114	15	0.1712	0.149	1018.8
NCAPH	0.271	0.274	0.288	0.279	0.288	0.204	0.128	0.158	0	0.109	0.378	0.207	0.209	0.138	0	0.26	0.302	15	0.232866667	0.26	734.6666667
HEATR1	0.27	0.238	0.253	0.205	0.233	0.132	0.118	0.312	0.109	0.172	0	0.142	0.121	0.136	0	0.162	0.147	15	0.183333333	0.162	981.4
HIST1H2BJ	0.268	0.235	0.23	0.208	0.232	0.108	0.137	0.299	0.109	0.187	0	0.134	0.106	0.138	0	0.11	0.126	15	0.175133333	0.138	1203.466667

CSTF2	0.267	0.242	0.221	0.209	0.207	0.135	0.134	0.26	0.103	0.171	0	0.135	0.111	0.113	0	0.139	0.133	15	0.172	0.139	1111.8
CPSF3	0.266	0.24	0.219	0.209	0.203	0.151	0.143	0.232	0.125	0.157	0	0.172	0.15	0.113	0	0.188	0.169	15	0.182466667	0.172	849.3333333
DMRTC2	0.266	0.261	0.324	0.239	0.314	0.181	0.12	0.306	0	0.183	0.201	0.159	0.155	0.125	0	0.107	0.15	15	0.206066667	0.183	868.3333333
SET	0.266	0.242	0.174	0.208	0.161	0.212	0.149	0.183	0	0.103	0.111	0.222	0.198	0	0.129	0.188	0.146	15	0.179466667	0.183	985.8666667
H3F3AP4	0.265	0.235	0.23	0.201	0.207	0.14	0.115	0.279	0	0.132	0.114	0.196	0.179	0.113	0	0.13	0.116	15	0.1768	0.179	978.5333333
PCF11	0.265	0.241	0.234	0.211	0.212	0.126	0.121	0.291	0	0.154	0	0.164	0.143	0.139	0.126	0.223	0.164	15	0.1876	0.164	904.8
ERI1	0.265	0.243	0.199	0.225	0.179	0.18	0.193	0.253	0	0.121	0	0.167	0.144	0.107	0.103	0.173	0.142	15	0.1796	0.179	1006.133333
SUPT6H	0.264	0.258	0.285	0.255	0.279	0.232	0.204	0.315	0.172	0.242	0	0.134	0.122	0.13	0	0.133	0.107	15	0.2088	0.232	899.4
PSMD3	0.264	0.231	0.215	0.208	0.202	0.114	0.127	0.291	0	0.147	0.129	0.172	0.148	0.112	0	0.163	0.136	15	0.177266667	0.163	968.0666667
PTBP2	0.262	0.219	0.204	0.179	0.172	0.128	0.123	0.262	0	0.108	0	0.18	0.148	0.102	0.11	0.168	0.131	15	0.1664	0.168	1158.333333
TIPARP	0.261	0.229	0.208	0.196	0.196	0.113	0.119	0.168	0	0.116	0.105	0.222	0.211	0.125	0	0.122	0.118	15	0.167266667	0.168	1194.666667
PLD6	0.256	0.246	0.297	0.224	0.288	0.105	0.115	0.331	0.147	0.235	0	0.123	0.107	0.143	0	0.127	0.139	15	0.1922	0.147	1135.266667
HIST1H2AM	0.255	0.228	0.153	0.179	0.14	0.111	0.122	0.19	0	0.103	0	0.17	0.148	0.13	0.166	0.169	0.127	15	0.1594	0.153	1315.333333
PIWIL1	0.254	0.237	0.223	0.198	0.207	0.105	0.1	0.289	0	0.145	0	0.145	0.129	0.137	0.176	0.186	0.147	15	0.178533333	0.176	1098.6
FILIP1L	0.254	0.229	0.289	0.217	0.285	0	0.126	0.292	0.246	0.283	0	0.148	0.121	0.14	0.151	0.123	0.128	15	0.202133333	0.217	963.4666667
RPS6KA2-AS1	0.252	0.233	0.228	0.251	0.219	0.144	0.166	0.226	0.134	0.178	0	0.141	0.135	0	0.144	0.176	0.143	15	0.184666667	0.176	932.4666667
CNOT11	0.252	0.222	0.203	0.189	0.19	0.136	0.127	0.277	0.109	0.134	0	0.154	0.128	0	0.113	0.177	0.162	15	0.171533333	0.162	1068.933333
ZNF331	0.252	0.217	0.158	0.183	0.139	0.113	0.121	0.217	0	0	0.149	0.24	0.193	0.109	0.169	0.192	0.151	15	0.173533333	0.169	1060.933333
DDX51	0.25	0.23	0.243	0.214	0.228	0.132	0.137	0.298	0	0.16	0	0.174	0.155	0.127	0.135	0.201	0.169	15	0.1902	0.174	840.1333333
MOV10L1	0.249	0.233	0.207	0.217	0.193	0.122	0.146	0.247	0	0.144	0	0.143	0.134	0.118	0.12	0.147	0.122	15	0.169466667	0.146	1145.2
CMTR2	0.248	0.239	0.161	0.192	0.152	0.137	0.119	0.145	0	0	0.139	0.172	0.153	0.126	0.142	0.175	0.194	15	0.166266667	0.153	1235.866667
PUM2	0.247	0.219	0.201	0.208	0.176	0.131	0.149	0.22	0	0.11	0.107	0.213	0.193	0	0.105	0.183	0.144	15	0.173733333	0.183	1012.8
PRPF4	0.245	0.221	0.2	0.184	0.19	0.112	0.114	0.253	0	0.139	0	0.167	0.144	0.132	0.129	0.189	0.175	15	0.172933333	0.175	1069.466667
CTDP1	0.244	0.227	0.233	0.196	0.214	0.107	0.122	0.247	0.106	0.196	0	0.132	0.118	0.155	0	0.186	0.139	15	0.1748	0.186	1104.466667
HMGA1	0.243	0.211	0.193	0.18	0.174	0.116	0.108	0.196	0.131	0.134	0	0.156	0.14	0	0.156	0.206	0.139	15	0.165533333	0.156	1175.066667
NHP2L1	0.241	0.229	0.324	0.218	0.315	0.105	0.106	0.421	0.152	0.24	0	0.13	0.105	0.15	0	0.114	0.125	15	0.198333333	0.152	1195.133333
GADD45AP1	0.24	0.218	0.169	0.205	0.165	0.132	0.111	0.164	0	0.138	0.22	0.244	0.182	0.131	0	0.131	0.125	15	0.171666667	0.165	1180.4
SNRNP200	0.238	0.212	0.247	0.195	0.237	0.141	0.138	0.322	0.101	0.161	0	0.123	0.1	0.121	0	0.107	0.114	15	0.170466667	0.141	1288.133333
SCARNA2	0.234	0.203	0.19	0.176	0.169	0.154	0.14	0.249	0.101	0.104	0	0.132	0.107	0	0.198	0.144	0.119	15	0.161333333	0.154	1285.666667
SRSF1	0.228	0.202	0.196	0.175	0.178	0.127	0.115	0.265	0	0.116	0	0.172	0.15	0.101	0.108	0.156	0.126	15	0.161	0.156	1246.733333

MCM5	0.225	0.215	0.144	0.217	0.138	0.116	0.206	0.154	0	0.122	0	0.139	0.138	0.11	0.264	0.229	0.22	15	0.1758	0.154	1312
BTBD1	0.224	0.203	0.242	0.226	0.236	0.18	0.216	0.246	0.102	0.156	0.143	0.162	0.153	0.103	0	0.103	0	15	0.179666667	0.18	988.0666667
STSP1	0.224	0.217	0.29	0.188	0.279	0.119	0.1	0.408	0.109	0.203	0	0.107	0.101	0.151	0	0.1	0.112	15	0.180533333	0.151	1434.666667
BLCAP	0.224	0.191	0.156	0.156	0.137	0.108	0.101	0.158	0.106	0.118	0	0.13	0.104	0	0.163	0.154	0.115	15	0.1414	0.137	1768.333333
POM121C	0.221	0.193	0.207	0.16	0.177	0.103	0.106	0.26	0.104	0.124	0	0.154	0.13	0	0.107	0.158	0.122	15	0.155066667	0.154	1390.333333
LOC729316	0.221	0.193	0.207	0.16	0.177	0.103	0.106	0.26	0.104	0.124	0	0.154	0.13	0	0.107	0.158	0.122	15	0.155066667	0.154	1391.333333
XRN2	0.219	0.209	0.225	0.197	0.209	0.164	0.146	0.256	0.126	0.175	0	0.124	0.109	0.122	0	0.126	0.123	15	0.168666667	0.164	1205.133333
FRA1E	0.219	0.197	0.187	0.181	0.168	0.138	0.163	0.202	0	0.107	0	0.159	0.149	0.111	0.105	0.127	0.115	15	0.1552	0.159	1373
KHNYN	0.21	0.194	0.195	0.166	0.182	0.115	0.122	0.244	0.106	0.123	0	0.192	0.179	0.103	0	0.125	0.115	15	0.158066667	0.166	1252.333333
LSM10	0.205	0.187	0.193	0.161	0.18	0.121	0.126	0.263	0	0.136	0	0.144	0.121	0.103	0.112	0.147	0.125	15	0.154933333	0.144	1410.266667
JTB	0.199	0.181	0.191	0.161	0.185	0.108	0	0.139	0	0.113	0.132	0.15	0.126	0.138	0.101	0.13	0.12	15	0.144933333	0.138	1707.866667
PTTG3P	0.196	0.179	0.256	0.18	0.247	0.103	0.117	0.245	0.292	0.245	0	0.125	0.109	0.101	0	0.164	0.117	15	0.1784	0.179	1354.733333
ZCCHC7	0.186	0.177	0.206	0.156	0.192	0.123	0.101	0.21	0.153	0.183	0	0.123	0.116	0.141	0	0.123	0.1	15	0.152666667	0.153	1577.133333

Table S4. DDR concept term interrogation of the latent semantic indexing (LSI)-based GPCR system corpus. The DDR-specific concept terms (Table S1) were applied as interrogators to the GPCR system corpus created in Table S2. For each specific protein, identified by its official gene symbol, the cosine similarity score (at least >0.1, therefore indicating an implicit association) for the strength of latent semantic association with the DDR-specific concept terms is given. The sum of the specific concept occurrences, the average and median cosine similarity score and the average rank within the matrix are given for each specific protein.

Gene symbol	Homologous recombination	DNA damage response	DNA damage	Double-strand break	Genotoxic stress	DNA damage repair	Single strand break	Cell-cycle checkpoint	Genotoxic	Base excision repair	Nucleotide excision repair	Cell cycle checkpoint	Senescence	DDR	DSB	Non-homologous end-joining	SSB	Sum of occurrences	Average score	Median score	Average rank
DHX8	0.372	0.299	0.283	0.285	0.165	0.262	0.278	0.189	0.144	0.159	0.168	0.192	0	0.152	0.196	0.11	0	15	0.216933333	0.192	7.066666667
CSTF2	0.26	0.267	0.242	0.221	0.135	0.209	0.207	0.139	0.111	0.135	0.134	0.133	0	0.113	0.171	0.103	0	15	0.172	0.139	16.33333333
TCEA1	0.264	0.276	0.264	0.213	0.185	0.255	0.197	0.141	0.173	0.236	0.221	0.118	0	0.126	0.152	0	0	14	0.2015	0.205	10.57142857
LOC647323	0.113	0.305	0.28	0.153	0.312	0.199	0.129	0.28	0.308	0	0	0.169	0.319	0.273	0.155	0	0	13	0.230384615	0.273	29.38461538
RFX8	0.113	0.305	0.28	0.153	0.312	0.199	0.129	0.28	0.308	0	0	0.169	0.319	0.273	0.155	0	0	13	0.230384615	0.273	28.38461538
QTRT1	0.108	0.319	0.303	0.132	0.239	0.265	0.13	0.234	0.232	0.14	0	0.344	0	0.146	0	0	0.245	13	0.218230769	0.234	33.38461538
PPP2R4	0.129	0.277	0.241	0.143	0.236	0.171	0.121	0.253	0.204	0	0	0.229	0.108	0.209	0.143	0	0	13	0.189538462	0.204	28.92307692
TSNAX	0.19	0.25	0.225	0.188	0.178	0.185	0.175	0.133	0.157	0	0.106	0.139	0	0.147	0.139	0	0	13	0.170153846	0.175	18.69230769
KIAA0368	0.279	0.236	0.192	0.199	0.181	0.168	0.186	0.134	0.128	0	0	0.136	0	0.125	0.147	0	0.101	13	0.170153846	0.168	14.92307692
POLR2J	0.22	0.212	0.184	0.147	0.167	0.167	0.131	0.185	0.143	0.153	0.138	0.15	0.155	0	0	0	0	13	0.165538462	0.155	20.23076923
GPR108	0.317	0.216	0.199	0.201	0.165	0.177	0.186	0.138	0.144	0.123	0.127	0.114	0	0	0.111	0	0	13	0.170615385	0.165	17.23076923
KIAA1033	0.23	0.221	0.189	0.184	0.16	0.159	0.164	0.138	0.138	0	0.104	0.1	0	0.135	0.123	0	0	13	0.157307692	0.159	23.76923077
PITPNC1	0.167	0.212	0.181	0.131	0.16	0.15	0.116	0.132	0.139	0.103	0.113	0.125	0	0	0	0	0	12	0.144083333	0.1355	36.66666667
ARF3	0.194	0.181	0.152	0.149	0.136	0.119	0.138	0.114	0.112	0	0.102	0.117	0	0	0	0	0.108	12	0.135166667	0.1275	39.58333333
OXR1	0.126	0.26	0.245	0.123	0.145	0.242	0.106	0	0.11	0.265	0.166	0	0	0	0	0	0.115	11	0.173	0.145	41.27272727
RNU6V	0.253	0.202	0.172	0.219	0.102	0.154	0.203	0	0	0	0	0	0.117	0.117	0.18	0.137	0	11	0.168727273	0.172	22.09090909
REXO1	0.177	0.184	0.165	0.171	0.115	0.134	0.163	0.109	0.101	0	0	0	0	0	0.139	0.122	0	11	0.143636364	0.139	38
EIF3J	0.191	0.177	0.156	0.141	0.12	0.148	0.129	0.102	0	0.104	0.153	0	0.119	0	0	0	0	11	0.14	0.141	41.72727273
TMEM181	0.145	0.202	0.167	0.122	0.118	0.121	0.107	0	0.106	0	0	0.113	0	0.149	0.102	0	0	11	0.132	0.121	51.09090909
DYNC1LI2	0.157	0.161	0.14	0.132	0.132	0.104	0.11	0.141	0.118	0	0	0.126	0	0.113	0	0	0	11	0.130363636	0.132	53.45454545
ANKRD30BL	0.272	0.227	0.195	0.198	0.108	0.162	0.175	0	0	0	0	0.118	0	0.132	0.137	0	0	10	0.1724	0.1685	21.1
POTEF	0.3	0.19	0.167	0.222	0.114	0.149	0.214	0	0.103	0	0	0	0	0	0.153	0.118	0	10	0.173	0.16	23.2
SPATC1	0.152	0.154	0.154	0.125	0.126	0.119	0.112	0.255	0.12	0	0	0.254	0	0	0	0	0	10	0.1571	0.139	52.1

PAIP2	0.18	0.203	0.167	0.147	0.157	0.132	0.133	0.156	0.127	0	0	0.113	0	0	0	0	0	10	0.1515	0.1515	34.3
TMEM217	0.183	0.174	0.149	0.138	0.196	0.12	0.126	0.114	0.178	0	0	0	0	0	0.116	0	0	10	0.1494	0.1435	40.4
NOL12	0.173	0.204	0.191	0.12	0.112	0.148	0.105	0.149	0	0	0	0.157	0.114	0	0	0	0	10	0.1473	0.1485	41.9
GPR32P1	0.197	0.156	0.141	0.144	0	0.137	0.126	0	0	0.148	0.161	0	0	0.104	0.135	0	0	10	0.1449	0.1425	37.4
GOLPH3	0.156	0.186	0.149	0.12	0.109	0.132	0.102	0.151	0	0	0	0.111	0.104	0	0	0	0	10	0.132	0.126	56
PPP5C	0	0.212	0.186	0.101	0.177	0.136	0	0.209	0.145	0	0	0.235	0	0.185	0	0	0	9	0.176222222	0.185	28
ATP9A	0.198	0.202	0.182	0.203	0.144	0.156	0.169	0	0.132	0	0.113	0	0	0	0	0	0	9	0.166555556	0.169	24.88888889
AASS	0.143	0.219	0.196	0.123	0.127	0.16	0.111	0	0	0.192	0.135	0	0	0	0	0	0	9	0.156222222	0.143	43.55555556
AFTPH	0.113	0.185	0.166	0	0.156	0.106	0	0.152	0.139	0	0	0.17	0	0.166	0	0	0	9	0.150333333	0.156	56.22222222
ZNF43	0.139	0.178	0.148	0.113	0.205	0.108	0	0.149	0.194	0	0	0.142	0	0	0	0	0	9	0.152888889	0.148	53.88888889
DENND1B	0.206	0.187	0.162	0.157	0.105	0.162	0.149	0	0	0.141	0.159	0	0	0	0	0	0	9	0.158666667	0.159	32.22222222
PPP1CB	0.115	0.167	0.141	0	0.146	0.101	0	0.213	0.121	0	0	0.202	0	0.107	0	0	0	9	0.145888889	0.141	62.77777778
PAIP1	0.225	0.18	0.154	0.192	0.11	0.133	0.179	0	0	0	0	0	0	0	0.132	0.114	0	9	0.157666667	0.154	31.88888889
OR6V1	0.107	0.223	0.183	0	0.1	0.149	0	0.127	0	0	0.109	0.138	0	0.136	0	0	0	9	0.141333333	0.136	61.77777778
OR52I2	0.107	0.223	0.183	0	0.1	0.149	0	0.127	0	0	0.109	0.138	0	0.136	0	0	0	9	0.141333333	0.136	64.77777778
OR52M1	0.107	0.223	0.183	0	0.1	0.149	0	0.127	0	0	0.109	0.138	0	0.136	0	0	0	9	0.141333333	0.136	60.77777778
OR51D1	0.107	0.223	0.183	0	0.1	0.149	0	0.127	0	0	0.109	0.138	0	0.136	0	0	0	9	0.141333333	0.136	62.77777778
OR52I1	0.107	0.223	0.183	0	0.1	0.149	0	0.127	0	0	0.109	0.138	0	0.136	0	0	0	9	0.141333333	0.136	63.77777778
GPR21	0.112	0.225	0.177	0	0.139	0.135	0	0.133	0.109	0	0.104	0.125	0	0	0	0	0	9	0.139888889	0.133	59.55555556
SLC6A16	0.225	0.201	0.158	0.163	0.123	0.139	0.135	0	0	0.111	0.117	0	0	0	0	0	0	9	0.152444444	0.139	32.88888889
WDR65	0.216	0.18	0.151	0.159	0.123	0.132	0.141	0.108	0	0	0.11	0	0	0	0	0	0	9	0.146666667	0.141	40.55555556
LOC440683	0.233	0.192	0.162	0.149	0.105	0.14	0.127	0	0	0.117	0.109	0	0	0	0	0	0	9	0.148222222	0.14	38.66666667
COPZ2	0.201	0.152	0.125	0.16	0	0.103	0.143	0.136	0	0	0	0	0	0	0.123	0.106	0	9	0.138777778	0.136	44.44444444
SNX33	0.137	0.151	0.129	0.123	0.119	0.114	0.106	0.138	0	0	0	0.15	0	0	0	0	0	9	0.129666667	0.129	71.22222222
VPS26B	0.244	0.146	0.124	0.148	0.103	0.104	0.133	0.109	0	0	0	0.108	0	0	0	0	0	9	0.135444444	0.124	61.44444444
TCEAL3	0.204	0.151	0.13	0.127	0.111	0.111	0.11	0	0	0.123	0.1	0	0	0	0	0	0	9	0.129666667	0.123	59.66666667
ARFGEF1	0.195	0.151	0.123	0.132	0.12	0.1	0.12	0.116	0	0	0	0.101	0	0	0	0	0	9	0.128666667	0.12	63
BRSK1	0	0.298	0.282	0	0.251	0.197	0	0.393	0.236	0	0	0.477	0	0.257	0	0	0	8	0.298875	0.2695	4.125
GPR87	0.112	0.208	0.175	0	0.283	0.127	0	0.163	0.277	0	0	0	0.222	0	0	0	0	8	0.195875	0.1915	49.25
PEX26	0.198	0.193	0.17	0.113	0.169	0.133	0	0.11	0.132	0	0	0	0	0	0	0	0	8	0.15225	0.151	41.125
WDR20	0.148	0.18	0.167	0.107	0.125	0.133	0	0.156	0	0	0	0.108	0	0	0	0	0	8	0.1405	0.1405	57.25
GPR52	0.233	0.18	0.149	0.148	0	0.124	0.126	0	0	0.117	0.118	0	0	0	0	0	0	8	0.149375	0.137	39.625

GPR53P	0.233	0.18	0.149	0.148	0	0.124	0.126	0	0	0.117	0.118	0	0	0	0	0	0	8	0.149375	0.137	38.625
OR51E1	0.154	0.187	0.14	0	0.125	0.115	0	0.12	0.105	0	0	0	0.166	0	0	0	0	8	0.139	0.1325	52.375
GPR33	0.152	0.171	0.142	0.103	0.107	0.124	0	0	0	0.166	0.133	0	0	0	0	0	0	8	0.13725	0.1375	65.25
ARF4P1	0.166	0.158	0.126	0.126	0	0.109	0.108	0	0	0.143	0.158	0	0	0	0	0	0	8	0.13675	0.1345	60.375
VAC14	0.231	0.168	0.133	0.156	0.107	0.105	0.127	0.117	0	0	0	0	0	0	0	0	0	8	0.143	0.13	54.5
FOLR1	0.124	0.196	0.151	0.106	0.102	0.124	0	0	0	0.107	0.116	0	0	0	0	0	0	8	0.12825	0.12	76.625
MYO16	0.246	0.161	0.127	0.148	0	0.112	0.127	0.103	0	0	0.103	0	0	0	0	0	0	8	0.140875	0.127	54.125
GBF1	0.201	0.16	0.118	0.132	0.124	0	0.119	0.114	0	0	0	0.103	0	0	0	0	0	8	0.133875	0.1215	57.375
RBAK	0.169	0.138	0.116	0.117	0	0.133	0.109	0.123	0	0	0	0	0.124	0	0	0	0	8	0.128625	0.1235	69.625
COPE	0.194	0.16	0.124	0.127	0.128	0.102	0.11	0	0	0	0.101	0	0	0	0	0	0	8	0.13075	0.1255	64.625
HIST2H2BD	0.179	0.135	0.12	0.127	0	0.109	0.116	0	0	0.133	0.109	0	0	0	0	0	0	8	0.1285	0.1235	67.125
ZNF326	0.118	0.138	0.114	0	0.281	0	0	0.101	0.271	0	0	0	0.239	0	0	0	0	7	0.180285714	0.138	78
RAB35	0.156	0.154	0.127	0	0.263	0	0	0.131	0.254	0	0	0	0.161	0	0	0	0	7	0.178	0.156	47.57142857
GPR156	0.181	0.145	0.11	0.189	0	0	0.172	0	0	0	0	0	0	0	0.171	0.233	0	7	0.171571429	0.172	45.85714286
PPAN-P2RY11	0.328	0.165	0.132	0.207	0	0.129	0.196	0	0	0	0	0	0	0	0.109	0	0	7	0.180857143	0.165	33
PPP1CC	0.127	0.166	0.136	0	0.131	0	0	0.18	0.105	0	0	0.179	0	0	0	0	0	7	0.146285714	0.136	64.14285714
SDS	0.146	0.186	0.154	0.116	0	0.125	0	0	0	0.162	0.11	0	0	0	0	0	0	7	0.142714286	0.146	56.85714286
OR1D2	0.215	0.169	0.144	0.157	0.106	0.114	0.137	0	0	0	0	0	0	0	0	0	0	7	0.148857143	0.144	50.85714286
MMADHC	0.179	0.15	0.131	0.16	0	0.104	0.146	0	0	0	0	0	0	0	0.107	0	0	7	0.139571429	0.146	55.85714286
NDUFAF5	0.134	0.137	0.127	0.127	0	0.123	0.122	0	0	0	0	0	0	0	0.115	0	0	7	0.126428571	0.127	81.71428571
GPR158	0.155	0.162	0.135	0.116	0.108	0.106	0	0	0	0.11	0	0	0	0	0	0	0	7	0.127428571	0.116	76.42857143
GPR27	0.189	0.175	0.13	0.106	0.103	0	0	0	0	0.113	0.105	0	0	0	0	0	0	7	0.131571429	0.113	66.28571429
GLUD1P5	0.182	0.15	0.124	0.117	0	0	0.107	0	0	0.126	0.106	0	0	0	0	0	0	7	0.130285714	0.124	65.57142857
VPS26A	0.178	0.152	0.126	0.12	0.106	0.105	0.103	0	0	0	0	0	0	0	0	0	0	7	0.127142857	0.12	80.85714286
IMPAD1	0.127	0.14	0.125	0.113	0.113	0	0	0	0.101	0.105	0	0	0	0	0	0	0	7	0.117714286	0.113	94.85714286
AAK1	0.128	0.127	0.101	0	0.14	0	0	0.102	0.12	0	0	0.107	0	0	0	0	0	7	0.117857143	0.12	100.4285714
PRKAR1AP	0.184	0.142	0.12	0.107	0	0.101	0	0	0	0.119	0.107	0	0	0	0	0	0	7	0.125714286	0.119	76
TRNAR2	0.115	0.176	0.164	0	0	0.168	0	0	0	0.313	0.191	0	0	0	0	0	0	6	0.187833333	0.172	60.83333333
TRNAV21	0.115	0.176	0.164	0	0	0.168	0	0	0	0.313	0.191	0	0	0	0	0	0	6	0.187833333	0.172	59.83333333
TRNAV32	0.115	0.176	0.164	0	0	0.168	0	0	0	0.313	0.191	0	0	0	0	0	0	6	0.187833333	0.172	61.83333333
CETN4P	0.11	0.148	0.138	0	0	0.146	0	0	0	0.196	0.246	0	0	0	0	0	0	6	0.164	0.147	81.5
GDPD5	0	0.186	0.156	0	0.14	0.103	0	0	0.119	0	0	0	0	0.155	0	0	0	6	0.143166667	0.1475	42.5
ACSM1	0.101	0.143	0.11	0	0	0.166	0	0	0	0.152	0.261	0	0	0	0	0	0	6	0.1555	0.1475	98.16666667

ILKAP	0	0.118	0	0	0.145	0	0	0.153	0.124	0	0	0.112	0.179	0	0	0	0	6	0.1385	0.1345	53.66666667
ANO7	0.137	0.165	0.13	0	0.19	0	0	0	0.176	0	0	0	0.127	0	0	0	0	6	0.15416667	0.151	58.66666667
GPR12	0.14	0.184	0.132	0	0.127	0	0	0.143	0	0	0	0.105	0	0	0	0	0	6	0.1385	0.136	64.5
OR5AC1	0.166	0.187	0.137	0.123	0.117	0	0	0.105	0	0	0	0	0	0	0	0	0	6	0.13916667	0.13	66.16666667
OR52Z1	0.166	0.187	0.137	0.123	0.117	0	0	0.105	0	0	0	0	0	0	0	0	0	6	0.13916667	0.13	63.16666667
OR4E1	0.166	0.187	0.137	0.123	0.117	0	0	0.105	0	0	0	0	0	0	0	0	0	6	0.13916667	0.13	64.16666667
OR10J4	0.166	0.187	0.137	0.123	0.117	0	0	0.105	0	0	0	0	0	0	0	0	0	6	0.13916667	0.13	65.16666667
GPR113	0.172	0.155	0.121	0.111	0.147	0	0	0	0.128	0	0	0	0	0	0	0	0	6	0.139	0.1375	69.83333333
ZSWIM8	0.249	0.133	0.114	0.16	0	0.107	0.143	0	0	0	0	0	0	0	0	0	0	6	0.151	0.138	65.33333333
NRG3-AS1	0.249	0.133	0.114	0.16	0	0.107	0.143	0	0	0	0	0	0	0	0	0	0	6	0.151	0.138	66.33333333
FAM65C	0.249	0.133	0.114	0.16	0	0.107	0.143	0	0	0	0	0	0	0	0	0	0	6	0.151	0.138	64.33333333
CDRT15P2	0.249	0.133	0.114	0.16	0	0.107	0.143	0	0	0	0	0	0	0	0	0	0	6	0.151	0.138	67.33333333
TBC1D3G	0.18	0.129	0.107	0.115	0.147	0	0	0	0.147	0	0	0	0	0	0	0	0	6	0.1375	0.138	81.83333333
TBC1D3B	0.18	0.129	0.107	0.115	0.147	0	0	0	0.147	0	0	0	0	0	0	0	0	6	0.1375	0.138	82.83333333
LOC653380	0.18	0.129	0.107	0.115	0.147	0	0	0	0.147	0	0	0	0	0	0	0	0	6	0.1375	0.138	79.83333333
TBC1D3H	0.18	0.129	0.107	0.115	0.147	0	0	0	0.147	0	0	0	0	0	0	0	0	6	0.1375	0.138	80.83333333
PDCL	0.123	0.145	0.113	0	0.157	0	0	0.112	0.115	0	0	0	0	0	0	0	0	6	0.1275	0.119	92.66666667
DGKB	0.144	0.144	0.109	0.126	0	0	0.101	0	0	0	0	0	0.147	0	0	0	0	6	0.1285	0.135	88
CSTF2T	0.179	0.147	0.127	0.127	0	0.106	0.116	0	0	0	0	0	0	0	0	0	0	6	0.13366667	0.127	74.33333333
C1ORF27	0.21	0.124	0.114	0.141	0	0.107	0.136	0	0	0	0	0	0	0	0	0	0	6	0.13866667	0.13	78.83333333
LCA5L	0.116	0.133	0.118	0.104	0	0	0	0.12	0	0	0	0.122	0	0	0	0	0	6	0.11883333	0.119	112.1666667
ARFGAP3	0.178	0.145	0.101	0.108	0.118	0	0	0	0	0	0	0	0	0	0	0	0.121	6	0.1285	0.1195	83.83333333
SNX21	0.134	0.152	0.11	0	0.106	0	0	0	0	0	0	0.101	0	0.113	0	0	0	6	0.11933333	0.1115	90.16666667
INSC	0.198	0.119	0.101	0.125	0	0	0.103	0.106	0	0	0	0	0	0	0	0	0	6	0.12533333	0.1125	97.5
SYNRG	0.136	0.121	0.101	0	0.112	0	0	0.104	0	0	0	0.109	0	0	0	0	0	6	0.11383333	0.1105	114
OPN5	0	0.123	0.11	0	0	0.109	0	0	0	0.143	0.149	0	0	0	0	0	0	5	0.1268	0.123	81
YIPF6	0.108	0.174	0.134	0	0.149	0	0	0	0.105	0	0	0	0	0	0	0	0	5	0.134	0.134	100
MCOLN2	0.147	0.119	0	0.154	0	0	0.14	0	0	0	0	0	0	0	0	0	0.122	5	0.1364	0.14	76.8
SLC13A5	0.142	0.148	0.121	0	0	0.132	0	0	0	0.117	0	0	0	0	0	0	0	5	0.132	0.132	84.2
COPG1	0.198	0.131	0.105	0.141	0	0	0.127	0	0	0	0	0	0	0	0	0	0	5	0.1404	0.131	83.4
BBIP1	0.192	0.14	0.115	0.133	0	0	0.113	0	0	0	0	0	0	0	0	0	0	5	0.1386	0.133	75.4
PEX11G	0.177	0.145	0.117	0.121	0	0	0.103	0	0	0	0	0	0	0	0	0	0	5	0.1326	0.121	87.8

PIGG	0.145	0.158	0.112	0.101	0.11	0	0	0	0	0	0	0	0	0	0	0	0	5	0.1252	0.112	105.6
MCIDAS	0.11	0.114	0	0	0.13	0	0	0.108	0.127	0	0	0	0	0	0	0	0	5	0.1178	0.114	125.2
COPA	0.181	0.135	0.108	0.121	0	0	0.11	0	0	0	0	0	0	0	0	0	0	5	0.131	0.121	92.4
ARFGAP1	0.159	0.136	0.105	0.11	0.111	0	0	0	0	0	0	0	0	0	0	0	0	5	0.1242	0.111	109.8
SSNA1	0.159	0.121	0	0.112	0	0	0	0.116	0	0	0	0.112	0	0	0	0	0	5	0.124	0.116	93.2
DLG2-AS1	0.104	0.111	0	0	0	0.11	0	0	0	0.103	0.123	0	0	0	0	0	0	5	0.1102	0.11	133.4
OR1D5	0.215	0.136	0.103	0.107	0	0	0	0	0	0.101	0	0	0	0	0	0	0	5	0.1324	0.107	92.6
AZGP1P2	0.183	0.129	0.102	0.105	0	0	0	0	0	0.104	0	0	0	0	0	0	0	5	0.1246	0.105	104.2
AZGP1P1	0.183	0.129	0.102	0.105	0	0	0	0	0	0.104	0	0	0	0	0	0	0	5	0.1246	0.105	105.2
PIP5K1C	0.191	0.109	0	0.114	0	0	0.101	0	0	0	0	0	0.113	0	0	0	0	5	0.1256	0.113	94.2
GPR180	0	0	0	0.212	0	0	0.213	0	0	0	0	0	0	0	0.218	0.254	0	4	0.22425	0.2155	3
ELTD1	0	0	0	0.163	0	0	0.145	0	0	0	0	0	0	0	0.181	0.266	0	4	0.18875	0.172	10.5
LOC100129726	0	0.114	0	0	0	0.131	0	0	0	0.167	0.231	0	0	0	0	0	0	4	0.16075	0.149	69.25
MTMR6	0	0.136	0.107	0	0.142	0	0	0	0.12	0	0	0	0	0	0	0	0	4	0.12625	0.128	84.5
PDE11A	0.26	0.108	0	0.142	0	0	0.137	0	0	0	0	0	0	0	0	0	0	4	0.16175	0.1395	81
TSPAN11	0.151	0.155	0.12	0.111	0	0	0	0	0	0	0	0	0	0	0	0	0	4	0.13425	0.1355	100.75
LPHN3	0.127	0.157	0.114	0.11	0	0	0	0	0	0	0	0	0	0	0	0	0	4	0.127	0.1205	123.5
GPR79	0.163	0.155	0.119	0.102	0	0	0	0	0	0	0	0	0	0	0	0	0	4	0.13475	0.137	100
ARRDC4	0.144	0.133	0	0	0	0	0	0	0	0.117	0.114	0	0	0	0	0	0	4	0.127	0.125	86.75
GPR34	0.164	0.147	0.109	0.105	0	0	0	0	0	0	0	0	0	0	0	0	0	4	0.13125	0.128	108
NAT8L	0.12	0.144	0.106	0	0	0	0	0	0	0	0.111	0	0	0	0	0	0	4	0.12025	0.1155	127.25
MVK	0.116	0.144	0.11	0	0	0	0	0	0	0.103	0	0	0	0	0	0	0	4	0.11825	0.113	130.75
ARL6	0.18	0.135	0.104	0.113	0	0	0	0	0	0	0	0	0	0	0	0	0	4	0.133	0.124	109.25
LPHN2	0.149	0.147	0.104	0.1	0	0	0	0	0	0	0	0	0	0	0	0	0	4	0.125	0.1255	125
RAB43	0.162	0.142	0.108	0.1	0	0	0	0	0	0	0	0	0	0	0	0	0	4	0.128	0.125	117.75
ARF1	0.163	0.116	0	0.108	0	0	0	0	0	0	0	0	0.125	0	0	0	0	4	0.128	0.1205	107
GPR111	0.155	0.128	0.105	0.113	0	0	0	0	0	0	0	0	0	0	0	0	0	4	0.12525	0.1205	129.25
GNG5P1	0.207	0	0	0.102	0	0	0	0	0	0.136	0.108	0	0	0	0	0	0	4	0.13825	0.122	52.5
BAI2	0.106	0.136	0.101	0	0.108	0	0	0	0	0	0	0	0	0	0	0	0	4	0.11275	0.107	167.75
GPRC5C	0.124	0.119	0.1	0	0	0	0	0	0	0.126	0	0	0	0	0	0	0	4	0.11725	0.1215	147.5
IGHV4-59	0.163	0.135	0.102	0.105	0	0	0	0	0	0	0	0	0	0	0	0	0	4	0.12625	0.12	124.25
PPAP2C	0.139	0.133	0.104	0	0	0	0	0.105	0	0	0	0	0	0	0	0	0	4	0.12025	0.119	127.25
GPR144	0.129	0	0	0.106	0.119	0	0	0	0.117	0	0	0	0	0	0	0	0	4	0.11775	0.118	99
AGAP1	0.162	0.127	0	0.101	0.11	0	0	0	0	0	0	0	0	0	0	0	0	4	0.125	0.1185	116

ITCH	0	0.11	0	0	0.17	0	0	0	0.165	0	0	0	0	0	0	0	0	3	0.148333333	0.165	84.66666667
GPR22	0	0.179	0.14	0	0	0.11	0	0	0	0	0	0	0	0	0	0	0	3	0.143	0.14	61.33333333
PTAFR	0	0.187	0.128	0	0	0.102	0	0	0	0	0	0	0	0	0	0	0	3	0.139	0.128	68.66666667
CATSPERB	0	0.109	0	0	0	0	0	0	0	0.129	0.169	0	0	0	0	0	0	3	0.135666667	0.129	89.33333333
MAP3K5	0	0.124	0	0	0.156	0	0	0	0.103	0	0	0	0	0	0	0	0	3	0.127666667	0.124	85.66666667
SPHK1	0	0.162	0.107	0	0.104	0	0	0	0	0	0	0	0	0	0	0	0	3	0.124333333	0.107	97.66666667
PDE9A	0	0.138	0	0.117	0	0	0.104	0	0	0	0	0	0	0	0	0	0	3	0.119666667	0.117	92.33333333
VN1R1	0	0	0	0.13	0	0	0.118	0	0	0	0	0	0	0	0	0	0.102	3	0.116666667	0.118	37.66666667
RAB22A	0.309	0	0	0.164	0	0	0.156	0	0	0	0	0	0	0	0	0	0	3	0.209666667	0.164	12.66666667
DUSP16	0	0.103	0	0	0.112	0	0	0.102	0	0	0	0	0	0	0	0	0	3	0.105666667	0.103	135
NLN	0.121	0.16	0.131	0	0	0	0	0	0	0	0	0	0	0	0	0	0	3	0.137333333	0.131	125.6666667
SPHK2	0.107	0.177	0.114	0	0	0	0	0	0	0	0	0	0	0	0	0	0	3	0.132666667	0.114	153.3333333
INPP4A	0.139	0.164	0.119	0	0	0	0	0	0	0	0	0	0	0	0	0	0	3	0.140666667	0.139	112
ARF4	0.147	0.118	0	0	0	0	0	0	0	0	0	0	0.158	0	0	0	0	3	0.141	0.147	114.3333333
IPCEF1	0.139	0.149	0.11	0	0	0	0	0	0	0	0	0	0	0	0	0	0	3	0.132666667	0.139	127.3333333
RLN2	0.133	0.146	0.101	0	0	0	0	0	0	0	0	0	0	0	0	0	0	3	0.126666667	0.133	147.3333333
SEC24A	0.196	0.134	0	0.111	0	0	0	0	0	0	0	0	0	0	0	0	0	3	0.147	0.134	94
GNG12	0.131	0.139	0.104	0	0	0	0	0	0	0	0	0	0	0	0	0	0	3	0.124666667	0.131	151.6666667
CLUL1	0.139	0.134	0.108	0	0	0	0	0	0	0	0	0	0	0	0	0	0	3	0.127	0.134	142.6666667
FRMPD1	0.143	0.133	0.108	0	0	0	0	0	0	0	0	0	0	0	0	0	0	3	0.128	0.133	141
OR7E24	0.149	0.126	0	0	0.112	0	0	0	0	0	0	0	0	0	0	0	0	3	0.129	0.126	123
TBC1D5	0.158	0.126	0.107	0	0	0	0	0	0	0	0	0	0	0	0	0	0	3	0.130333333	0.126	138
ESRRAP1	0.113	0.125	0.106	0	0	0	0	0	0	0	0	0	0	0	0	0	0	3	0.114666667	0.113	193.3333333
ESRRAP2	0.113	0.125	0.106	0	0	0	0	0	0	0	0	0	0	0	0	0	0	3	0.114666667	0.113	192.3333333
RINL	0.248	0	0	0.119	0	0	0.112	0	0	0	0	0	0	0	0	0	0	3	0.159666667	0.119	50
AMPD2	0.169	0.128	0	0	0	0	0	0	0	0.102	0	0	0	0	0	0	0	3	0.133	0.128	100.6666667
PSD	0.109	0.12	0	0	0	0	0	0	0	0	0	0	0	0.109	0	0	0	3	0.112666667	0.109	168
SPACA3	0.143	0.127	0.101	0	0	0	0	0	0	0	0	0	0	0	0	0	0	3	0.123666667	0.127	156.6666667
ANKRD13C	0.121	0.126	0	0	0.102	0	0	0	0	0	0	0	0	0	0	0	0	3	0.116333333	0.121	162.3333333
INTU	0.132	0.12	0	0	0	0	0	0	0	0	0	0	0.105	0	0	0	0	3	0.119	0.12	134.6666667
MKNK2	0.1	0.11	0	0	0	0	0	0.114	0	0	0	0	0	0	0	0	0	3	0.108	0.11	200.6666667
LPAR4	0.101	0.11	0	0	0	0	0	0	0	0	0	0	0.112	0	0	0	0	3	0.107666667	0.11	189.3333333

GNG10	0.157	0.11	0	0.105	0	0	0	0	0	0	0	0	0	0	0	0	0	3	0.124	0.11	148.66666 67
COQ4	0.141	0.11	0.102	0	0	0	0	0	0	0	0	0	0	0	0	0	0	3	0.1176666 67	0.11	178.33333 33
AP4M1	0.171	0.109	0	0.101	0	0	0	0	0	0	0	0	0	0	0	0	0	3	0.127	0.109	147
GPRC5B	0.117	0.107	0	0	0	0	0	0	0	0.101	0	0	0	0	0	0	0	3	0.1083333 33	0.107	179
TPST2	0.112	0.102	0	0.105	0	0	0	0	0	0	0	0	0	0	0	0	0	3	0.1063333 33	0.105	219
RGS6	0	0.165	0.149	0	0	0	0	0	0	0	0	0	0	0	0	0	0	2	0.157	0.157	61.5
MIR342	0	0.152	0.129	0	0	0	0	0	0	0	0	0	0	0	0	0	0	2	0.1405	0.1405	86.5
TRNAA2	0	0.135	0.109	0	0	0	0	0	0	0	0	0	0	0	0	0	0	2	0.122	0.122	131.5
TRNAA3	0	0.135	0.109	0	0	0	0	0	0	0	0	0	0	0	0	0	0	2	0.122	0.122	130.5
PDE1A	0	0.13	0	0	0.106	0	0	0	0	0	0	0	0	0	0	0	0	2	0.118	0.118	120.5
GPR65	0	0.11	0	0	0	0	0	0	0	0	0	0	0.118	0	0	0	0	2	0.114	0.114	123
CCER1	0	0.116	0	0	0	0	0	0	0	0	0	0	0.109	0	0	0	0	2	0.1125	0.1125	117
CABIN1	0	0.117	0	0	0	0	0	0.1	0	0	0	0	0	0	0	0	0	2	0.1085	0.1085	137.5
ASAP2	0	0	0	0	0	0	0	0	0	0.109	0.108	0	0	0	0	0	0	2	0.1085	0.1085	39
GPR161	0.123	0	0	0	0	0	0	0	0	0	0	0	0.187	0	0	0	0	2	0.155	0.155	110.5
ARL1	0.145	0	0	0	0	0	0	0	0	0	0	0	0.16	0	0	0	0	2	0.1525	0.1525	74
ACKR2	0.12	0.154	0	0	0	0	0	0	0	0	0	0	0	0	0	0	0	2	0.137	0.137	158.5
CATSPER1	0.121	0.136	0	0	0	0	0	0	0	0	0	0	0	0	0	0	0	2	0.1285	0.1285	177.5
RLN1	0.12	0.136	0	0	0	0	0	0	0	0	0	0	0	0	0	0	0	2	0.128	0.128	178.5
RXFP1	0.121	0.135	0	0	0	0	0	0	0	0	0	0	0	0	0	0	0	2	0.128	0.128	180
RGS1	0.16	0.133	0	0	0	0	0	0	0	0	0	0	0	0	0	0	0	2	0.1465	0.1465	127.5
EMR2	0.106	0.131	0	0	0	0	0	0	0	0	0	0	0	0	0	0	0	2	0.1185	0.1185	229
GNG3	0.171	0.13	0	0	0	0	0	0	0	0	0	0	0	0	0	0	0	2	0.1505	0.1505	121
GPR139	0.175	0.129	0	0	0	0	0	0	0	0	0	0	0	0	0	0	0	2	0.152	0.152	120.5
GPR3	0.118	0.128	0	0	0	0	0	0	0	0	0	0	0	0	0	0	0	2	0.123	0.123	199.5
GPR85	0.115	0.127	0	0	0	0	0	0	0	0	0	0	0	0	0	0	0	2	0.121	0.121	207.5
GPR115	0.137	0.125	0	0	0	0	0	0	0	0	0	0	0	0	0	0	0	2	0.131	0.131	173.5
ARFGAP2	0.172	0	0	0	0	0	0	0.125	0	0	0	0	0	0	0	0	0	2	0.1485	0.1485	60.5
SCTR	0.134	0.124	0	0	0	0	0	0	0	0	0	0	0	0	0	0	0	2	0.129	0.129	179.5
PDE8A	0.108	0.124	0	0	0	0	0	0	0	0	0	0	0	0	0	0	0	2	0.116	0.116	236.5
GTF3A	0.114	0.121	0	0	0	0	0	0	0	0	0	0	0	0	0	0	0	2	0.1175	0.1175	221.5
GPR20	0.144	0.119	0	0	0	0	0	0	0	0	0	0	0	0	0	0	0	2	0.1315	0.1315	169.5
GPR56	0.111	0	0	0.118	0	0	0	0	0	0	0	0	0	0	0	0	0	2	0.1145	0.1145	177
SNX1	0.149	0.117	0	0	0	0	0	0	0	0	0	0	0	0	0	0	0	2	0.133	0.133	165

CGB1	0.172	0.117	0	0	0	0	0	0	0	0	0	0	0	0	0	0	0	2	0.1445	0.1445	142.5
C16ORF89	0.183	0	0	0.116	0	0	0	0	0	0	0	0	0	0	0	0	0	2	0.1495	0.1495	69.5
PLEK	0.124	0.115	0	0	0	0	0	0	0	0	0	0	0	0	0	0	0	2	0.1195	0.1195	210.5
GPRC6A	0.107	0.115	0	0	0	0	0	0	0	0	0	0	0	0	0	0	0	2	0.111	0.111	255
ATP6V1E2	0.161	0	0	0	0	0	0	0	0	0.115	0	0	0	0	0	0	0	2	0.138	0.138	67
SLC38A2	0.101	0.113	0	0	0	0	0	0	0	0	0	0	0	0	0	0	0	2	0.107	0.107	268
EMR4P	0.14	0	0	0	0	0	0	0	0	0.112	0	0	0	0	0	0	0	2	0.126	0.126	95.5
AKAP11	0.129	0.111	0	0	0	0	0	0	0	0	0	0	0	0	0	0	0	2	0.12	0.12	207.5
YIF1A	0.215	0	0	0.111	0	0	0	0	0	0	0	0	0	0	0	0	0	2	0.163	0.163	64
GNAQP1	0.143	0.11	0	0	0	0	0	0	0	0	0	0	0	0	0	0	0	2	0.1265	0.1265	187.5
CYTH3	0.152	0.109	0	0	0	0	0	0	0	0	0	0	0	0	0	0	0	2	0.1305	0.1305	176.5
WDR27	0.142	0	0	0	0	0	0	0.109	0	0	0	0	0	0	0	0	0	2	0.1255	0.1255	102.5
SLC24A1	0.134	0.108	0	0	0	0	0	0	0	0	0	0	0	0	0	0	0	2	0.121	0.121	210
AP1S1	0.128	0.108	0	0	0	0	0	0	0	0	0	0	0	0	0	0	0	2	0.118	0.118	218
PDE1C	0.105	0.108	0	0	0	0	0	0	0	0	0	0	0	0	0	0	0	2	0.1065	0.1065	275
GPR123	0.11	0.108	0	0	0	0	0	0	0	0	0	0	0	0	0	0	0	2	0.109	0.109	263.5
ADCY3	0.101	0.106	0	0	0	0	0	0	0	0	0	0	0	0	0	0	0	2	0.1035	0.1035	284.5
RPLP2	0.128	0.106	0	0	0	0	0	0	0	0	0	0	0	0	0	0	0	2	0.117	0.117	224.5
PITPNM3	0.113	0.105	0	0	0	0	0	0	0	0	0	0	0	0	0	0	0	2	0.109	0.109	259.5
PDE2A	0.114	0.105	0	0	0	0	0	0	0	0	0	0	0	0	0	0	0	2	0.1095	0.1095	258
GUCY2GP	0.135	0.105	0	0	0	0	0	0	0	0	0	0	0	0	0	0	0	2	0.12	0.12	214.5
APPL2	0.121	0.105	0	0	0	0	0	0	0	0	0	0	0	0	0	0	0	2	0.113	0.113	240
REP15	0.111	0.105	0	0	0	0	0	0	0	0	0	0	0	0	0	0	0	2	0.108	0.108	268
DNM3	0.147	0.104	0	0	0	0	0	0	0	0	0	0	0	0	0	0	0	2	0.1255	0.1255	199.5
CHM	0.118	0.104	0	0	0	0	0	0	0	0	0	0	0	0	0	0	0	2	0.111	0.111	247.5
OR2B2	0.142	0.104	0	0	0	0	0	0	0	0	0	0	0	0	0	0	0	2	0.123	0.123	207.5
GPR42	0.114	0.103	0	0	0	0	0	0	0	0	0	0	0	0	0	0	0	2	0.1085	0.1085	261.5
OR1G1	0.112	0.103	0	0	0	0	0	0	0	0	0	0	0	0	0	0	0	2	0.1075	0.1075	270
BPNT1	0.115	0.103	0	0	0	0	0	0	0	0	0	0	0	0	0	0	0	2	0.109	0.109	260
RAB1A	0.156	0.102	0	0	0	0	0	0	0	0	0	0	0	0	0	0	0	2	0.129	0.129	193.5
AKAP6	0.114	0.102	0	0	0	0	0	0	0	0	0	0	0	0	0	0	0	2	0.108	0.108	262
MBOAT2	0.109	0.102	0	0	0	0	0	0	0	0	0	0	0	0	0	0	0	2	0.1055	0.1055	284
RAB6A	0.179	0.102	0	0	0	0	0	0	0	0	0	0	0	0	0	0	0	2	0.1405	0.1405	174.5
GRK6P1	0.139	0	0	0.101	0	0	0	0	0	0	0	0	0	0	0	0	0	2	0.12	0.12	143

CHMP4A	0.182	0	0	0.101	0	0	0	0	0	0	0	0	0	0	0	0	0	2	0.1415	0.1415	90
CLTCL1	0.152	0.1	0	0	0	0	0	0	0	0	0	0	0	0	0	0	0	2	0.126	0.126	203
RNU3P1	0.147	0.1	0	0	0	0	0	0	0	0	0	0	0	0	0	0	0	2	0.1235	0.1235	208.5
PYGB	0.106	0.1	0	0	0	0	0	0	0	0	0	0	0	0	0	0	0	2	0.103	0.103	293
GPSM2	0.133	0	0	0	0	0	0	0	0	0	0	0.1	0	0	0	0	0	2	0.1165	0.1165	113
MIR138-2	0	0	0	0	0	0	0	0	0	0	0	0	0.163	0	0	0	0	1	0.163	0.163	8
HRH4	0	0.16	0	0	0	0	0	0	0	0	0	0	0	0	0	0	0	1	0.16	0.16	81
GPR183	0	0.145	0	0	0	0	0	0	0	0	0	0	0	0	0	0	0	1	0.145	0.145	111
OXER1	0	0.142	0	0	0	0	0	0	0	0	0	0	0	0	0	0	0	1	0.142	0.142	118
TCEAL4	0	0	0	0	0	0	0	0	0	0	0	0	0.14	0	0	0	0	1	0.14	0.14	14
PDE1B	0	0.138	0	0	0	0	0	0	0	0	0	0	0	0	0	0	0	1	0.138	0.138	124
HCAR1	0	0.135	0	0	0	0	0	0	0	0	0	0	0	0	0	0	0	1	0.135	0.135	140
NMUR1	0	0.127	0	0	0	0	0	0	0	0	0	0	0	0	0	0	0	1	0.127	0.127	172
NMUR2	0	0.127	0	0	0	0	0	0	0	0	0	0	0	0	0	0	0	1	0.127	0.127	169
DGKE	0	0.124	0	0	0	0	0	0	0	0	0	0	0	0	0	0	0	1	0.124	0.124	180
PDE7A	0	0.122	0	0	0	0	0	0	0	0	0	0	0	0	0	0	0	1	0.122	0.122	186
GPR83	0	0.121	0	0	0	0	0	0	0	0	0	0	0	0	0	0	0	1	0.121	0.121	189
CD97	0	0.12	0	0	0	0	0	0	0	0	0	0	0	0	0	0	0	1	0.12	0.12	191
PDE3A	0	0.12	0	0	0	0	0	0	0	0	0	0	0	0	0	0	0	1	0.12	0.12	192
NCS1	0	0.119	0	0	0	0	0	0	0	0	0	0	0	0	0	0	0	1	0.119	0.119	199
GPR26	0	0.118	0	0	0	0	0	0	0	0	0	0	0	0	0	0	0	1	0.118	0.118	201
COX7B	0	0.118	0	0	0	0	0	0	0	0	0	0	0	0	0	0	0	1	0.118	0.118	203
HCAR2	0	0.117	0	0	0	0	0	0	0	0	0	0	0	0	0	0	0	1	0.117	0.117	206
HRH2	0	0.117	0	0	0	0	0	0	0	0	0	0	0	0	0	0	0	1	0.117	0.117	208
NTSR2	0	0.117	0	0	0	0	0	0	0	0	0	0	0	0	0	0	0	1	0.117	0.117	209
WDR26	0	0.116	0	0	0	0	0	0	0	0	0	0	0	0	0	0	0	1	0.116	0.116	212
GPR35	0	0.115	0	0	0	0	0	0	0	0	0	0	0	0	0	0	0	1	0.115	0.115	213
S1PR5	0	0.114	0	0	0	0	0	0	0	0	0	0	0	0	0	0	0	1	0.114	0.114	217
GPR17	0	0.112	0	0	0	0	0	0	0	0	0	0	0	0	0	0	0	1	0.112	0.112	220
UGT3A2	0	0	0	0	0	0	0	0	0	0.112	0	0	0	0	0	0	0	1	0.112	0.112	36
SSH1	0	0	0	0	0	0	0	0.112	0	0	0	0	0	0	0	0	0	1	0.112	0.112	49
DGKZ	0	0.111	0	0	0	0	0	0	0	0	0	0	0	0	0	0	0	1	0.111	0.111	223
GPR50	0	0.11	0	0	0	0	0	0	0	0	0	0	0	0	0	0	0	1	0.11	0.11	231
NTSR1	0	0.11	0	0	0	0	0	0	0	0	0	0	0	0	0	0	0	1	0.11	0.11	229

BEST3	0	0	0	0	0	0	0	0	0	0	0	0	0	0	0	0	0.11	1	0.11	0.11	5
FFAR1	0	0.109	0	0	0	0	0	0	0	0	0	0	0	0	0	0	0	1	0.109	0.109	236
PRKD1	0	0.109	0	0	0	0	0	0	0	0	0	0	0	0	0	0	0	1	0.109	0.109	233
ADAMTS18	0	0.108	0	0	0	0	0	0	0	0	0	0	0	0	0	0	0	1	0.108	0.108	242
UTS2R	0	0.108	0	0	0	0	0	0	0	0	0	0	0	0	0	0	0	1	0.108	0.108	239
SLC5A5	0	0	0	0	0	0	0	0	0	0.108	0	0	0	0	0	0	0	1	0.108	0.108	40
ADCY8	0	0.107	0	0	0	0	0	0	0	0	0	0	0	0	0	0	0	1	0.107	0.107	246
P2RY14	0	0.107	0	0	0	0	0	0	0	0	0	0	0	0	0	0	0	1	0.107	0.107	248
RGS3	0	0.107	0	0	0	0	0	0	0	0	0	0	0	0	0	0	0	1	0.107	0.107	249
VSNL1	0	0.106	0	0	0	0	0	0	0	0	0	0	0	0	0	0	0	1	0.106	0.106	252
GUCY1A2	0	0.106	0	0	0	0	0	0	0	0	0	0	0	0	0	0	0	1	0.106	0.106	254
P2RY6	0	0.106	0	0	0	0	0	0	0	0	0	0	0	0	0	0	0	1	0.106	0.106	253
NTS	0	0.105	0	0	0	0	0	0	0	0	0	0	0	0	0	0	0	1	0.105	0.105	255
FFAR3	0	0.105	0	0	0	0	0	0	0	0	0	0	0	0	0	0	0	1	0.105	0.105	260
PDC	0	0.105	0	0	0	0	0	0	0	0	0	0	0	0	0	0	0	1	0.105	0.105	257
ADCY10	0	0.105	0	0	0	0	0	0	0	0	0	0	0	0	0	0	0	1	0.105	0.105	263
HRH1	0	0.104	0	0	0	0	0	0	0	0	0	0	0	0	0	0	0	1	0.104	0.104	266
LPAR1	0	0.104	0	0	0	0	0	0	0	0	0	0	0	0	0	0	0	1	0.104	0.104	267
AKAP3	0	0.103	0	0	0	0	0	0	0	0	0	0	0	0	0	0	0	1	0.103	0.103	273
GPR4	0	0.102	0	0	0	0	0	0	0	0	0	0	0	0	0	0	0	1	0.102	0.102	279
HTR4	0	0.102	0	0	0	0	0	0	0	0	0	0	0	0	0	0	0	1	0.102	0.102	280
S1PR2	0	0.102	0	0	0	0	0	0	0	0	0	0	0	0	0	0	0	1	0.102	0.102	276
PDE4B	0	0.101	0	0	0	0	0	0	0	0	0	0	0	0	0	0	0	1	0.101	0.101	282
NKPD1	0	0	0	0	0	0	0	0	0	0	0	0	0.101	0	0	0	0	1	0.101	0.101	28
TBC1D8	0	0	0	0	0	0	0	0.101	0	0	0	0	0	0	0	0	0	1	0.101	0.101	68
GALR3	0	0.1	0	0	0	0	0	0	0	0	0	0	0	0	0	0	0	1	0.1	0.1	283
GNG5P2	0.185	0	0	0	0	0	0	0	0	0	0	0	0	0	0	0	0	1	0.185	0.185	51
RAB21	0.123	0	0	0	0	0	0	0	0	0	0	0	0	0	0	0	0	1	0.123	0.123	214
GGCT	0.122	0	0	0	0	0	0	0	0	0	0	0	0	0	0	0	0	1	0.122	0.122	217
KIF3A	0.132	0	0	0	0	0	0	0	0	0	0	0	0	0	0	0	0	1	0.132	0.132	183
RXFP3	0.101	0	0	0	0	0	0	0	0	0	0	0	0	0	0	0	0	1	0.101	0.101	321
PLCH2	0.1	0	0	0	0	0	0	0	0	0	0	0	0	0	0	0	0	1	0.1	0.1	324
MAMDC4	0.128	0	0	0	0	0	0	0	0	0	0	0	0	0	0	0	0	1	0.128	0.128	197
ANAPC16	0.18	0	0	0	0	0	0	0	0	0	0	0	0	0	0	0	0	1	0.18	0.18	65

SNAP91	0.136	0	0	0	0	0	0	0	0	0	0	0	0	0	0	0	0	1	0.136	0.136	172
CHMP4B	0.179	0	0	0	0	0	0	0	0	0	0	0	0	0	0	0	0	1	0.179	0.179	69
AP1G1	0.131	0	0	0	0	0	0	0	0	0	0	0	0	0	0	0	0	1	0.131	0.131	189
GPR75	0.177	0	0	0	0	0	0	0	0	0	0	0	0	0	0	0	0	1	0.177	0.177	75
CLTB	0.124	0	0	0	0	0	0	0	0	0	0	0	0	0	0	0	0	1	0.124	0.124	208
CYTH4	0.176	0	0	0	0	0	0	0	0	0	0	0	0	0	0	0	0	1	0.176	0.176	78
PHLDB2	0.138	0	0	0	0	0	0	0	0	0	0	0	0	0	0	0	0	1	0.138	0.138	166
ARFGEF2	0.164	0	0	0	0	0	0	0	0	0	0	0	0	0	0	0	0	1	0.164	0.164	95
GGA2	0.134	0	0	0	0	0	0	0	0	0	0	0	0	0	0	0	0	1	0.134	0.134	176
CLTC	0.157	0	0	0	0	0	0	0	0	0	0	0	0	0	0	0	0	1	0.157	0.157	106
KCNIP1	0.131	0	0	0	0	0	0	0	0	0	0	0	0	0	0	0	0	1	0.131	0.131	186
EPN2	0.156	0	0	0	0	0	0	0	0	0	0	0	0	0	0	0	0	1	0.156	0.156	109
EPS15	0.129	0	0	0	0	0	0	0	0	0	0	0	0	0	0	0	0	1	0.129	0.129	193
MPP1	0.155	0	0	0	0	0	0	0	0	0	0	0	0	0	0	0	0	1	0.155	0.155	114
KCNJ14	0.127	0	0	0	0	0	0	0	0	0	0	0	0	0	0	0	0	1	0.127	0.127	204
KDELR3	0.15	0	0	0	0	0	0	0	0	0	0	0	0	0	0	0	0	1	0.15	0.15	122
PQBP4	0.124	0	0	0	0	0	0	0	0	0	0	0	0	0	0	0	0	1	0.124	0.124	212
AP4B1	0.148	0	0	0	0	0	0	0	0	0	0	0	0	0	0	0	0	1	0.148	0.148	126
RAB5A	0.138	0	0	0	0	0	0	0	0	0	0	0	0	0	0	0	0	1	0.138	0.138	165
SEC24D	0.148	0	0	0	0	0	0	0	0	0	0	0	0	0	0	0	0	1	0.148	0.148	127
ARF5	0.136	0	0	0	0	0	0	0	0	0	0	0	0	0	0	0	0	1	0.136	0.136	171
CYTH2	0.146	0	0	0	0	0	0	0	0	0	0	0	0	0	0	0	0	1	0.146	0.146	134
ARF6	0.135	0	0	0	0	0	0	0	0	0	0	0	0	0	0	0	0	1	0.135	0.135	174
FCHO1	0.145	0	0	0	0	0	0	0	0	0	0	0	0	0	0	0	0	1	0.145	0.145	137
GUCA1C	0.134	0	0	0	0	0	0	0	0	0	0	0	0	0	0	0	0	1	0.134	0.134	180
GNG8	0.144	0	0	0	0	0	0	0	0	0	0	0	0	0	0	0	0	1	0.144	0.144	141
RAB26	0.132	0	0	0	0	0	0	0	0	0	0	0	0	0	0	0	0	1	0.132	0.132	185
AP1S3	0.144	0	0	0	0	0	0	0	0	0	0	0	0	0	0	0	0	1	0.144	0.144	142
IFT52	0.131	0	0	0	0	0	0	0	0	0	0	0	0	0	0	0	0	1	0.131	0.131	187
EPN1	0.144	0	0	0	0	0	0	0	0	0	0	0	0	0	0	0	0	1	0.144	0.144	143
IGHV1-3	0.13	0	0	0	0	0	0	0	0	0	0	0	0	0	0	0	0	1	0.13	0.13	190
AP1S2	0.142	0	0	0	0	0	0	0	0	0	0	0	0	0	0	0	0	1	0.142	0.142	150
VPS25	0.128	0	0	0	0	0	0	0	0	0	0	0	0	0	0	0	0	1	0.128	0.128	195
PDE4C	0.142	0	0	0	0	0	0	0	0	0	0	0	0	0	0	0	0	1	0.142	0.142	153

MYPOP	0.127	0	0	0	0	0	0	0	0	0	0	0	0	0	0	0	0	1	0.127	0.127	202
AP1M2	0.14	0	0	0	0	0	0	0	0	0	0	0	0	0	0	0	0	1	0.14	0.14	155
CLTA	0.125	0	0	0	0	0	0	0	0	0	0	0	0	0	0	0	0	1	0.125	0.125	206
AP2A1	0.101	0	0	0	0	0	0	0	0	0	0	0	0	0	0	0	0	1	0.101	0.101	322
WDR44	0.124	0	0	0	0	0	0	0	0	0	0	0	0	0	0	0	0	1	0.124	0.124	211
GMPR	0.139	0	0	0	0	0	0	0	0	0	0	0	0	0	0	0	0	1	0.139	0.139	163
GNG13	0.101	0	0	0	0	0	0	0	0	0	0	0	0	0	0	0	0	1	0.101	0.101	320
CLINT1	0.123	0	0	0	0	0	0	0	0	0	0	0	0	0	0	0	0	1	0.123	0.123	213
RELL2	0.113	0	0	0	0	0	0	0	0	0	0	0	0	0	0	0	0	1	0.113	0.113	256
NETO2	0.122	0	0	0	0	0	0	0	0	0	0	0	0	0	0	0	0	1	0.122	0.122	218
ADAP1	0.116	0	0	0	0	0	0	0	0	0	0	0	0	0	0	0	0	1	0.116	0.116	240
DENND1C	0.121	0	0	0	0	0	0	0	0	0	0	0	0	0	0	0	0	1	0.121	0.121	221
TBC1D24	0.113	0	0	0	0	0	0	0	0	0	0	0	0	0	0	0	0	1	0.113	0.113	261
TULP3	0.121	0	0	0	0	0	0	0	0	0	0	0	0	0	0	0	0	1	0.121	0.121	224
SLC6A18	0.114	0	0	0	0	0	0	0	0	0	0	0	0	0	0	0	0	1	0.114	0.114	252
AP2M1	0.12	0	0	0	0	0	0	0	0	0	0	0	0	0	0	0	0	1	0.12	0.12	228
GNG5	0.113	0	0	0	0	0	0	0	0	0	0	0	0	0	0	0	0	1	0.113	0.113	259
SERPINA7	0.118	0	0	0	0	0	0	0	0	0	0	0	0	0	0	0	0	1	0.118	0.118	232
SMAP1	0.112	0	0	0	0	0	0	0	0	0	0	0	0	0	0	0	0	1	0.112	0.112	268
STARD3NL	0.118	0	0	0	0	0	0	0	0	0	0	0	0	0	0	0	0	1	0.118	0.118	233
CHST9	0.116	0	0	0	0	0	0	0	0	0	0	0	0	0	0	0	0	1	0.116	0.116	241
AP1G2	0.117	0	0	0	0	0	0	0	0	0	0	0	0	0	0	0	0	1	0.117	0.117	235
OR2AG1	0.113	0	0	0	0	0	0	0	0	0	0	0	0	0	0	0	0	1	0.113	0.113	255
FCHO2	0.117	0	0	0	0	0	0	0	0	0	0	0	0	0	0	0	0	1	0.117	0.117	236
RELL1	0.113	0	0	0	0	0	0	0	0	0	0	0	0	0	0	0	0	1	0.113	0.113	257
RAB32	0.117	0	0	0	0	0	0	0	0	0	0	0	0	0	0	0	0	1	0.117	0.117	237
RGSL1	0.113	0	0	0	0	0	0	0	0	0	0	0	0	0	0	0	0	1	0.113	0.113	260
RGS21	0.117	0	0	0	0	0	0	0	0	0	0	0	0	0	0	0	0	1	0.117	0.117	238
RGS18	0.113	0	0	0	0	0	0	0	0	0	0	0	0	0	0	0	0	1	0.113	0.113	264
CYTH1	0.112	0	0	0	0	0	0	0	0	0	0	0	0	0	0	0	0	1	0.112	0.112	272
PIP4K2A	0.112	0	0	0	0	0	0	0	0	0	0	0	0	0	0	0	0	1	0.112	0.112	274
GDA	0.112	0	0	0	0	0	0	0	0	0	0	0	0	0	0	0	0	1	0.112	0.112	273
PRKAR2B	0.108	0	0	0	0	0	0	0	0	0	0	0	0	0	0	0	0	1	0.108	0.108	291
KCNJ4	0.108	0	0	0	0	0	0	0	0	0	0	0	0	0	0	0	0	1	0.108	0.108	290

RAB11B	0.111	0	0	0	0	0	0	0	0	0	0	0	0	0	0	0	0	1	0.111	0.111	278
GDPD2	0.109	0	0	0	0	0	0	0	0	0	0	0	0	0	0	0	0	1	0.109	0.109	285
GNB5	0.11	0	0	0	0	0	0	0	0	0	0	0	0	0	0	0	0	1	0.11	0.11	279
AP2A2	0.109	0	0	0	0	0	0	0	0	0	0	0	0	0	0	0	0	1	0.109	0.109	284
AP1B1	0.11	0	0	0	0	0	0	0	0	0	0	0	0	0	0	0	0	1	0.11	0.11	281
REEP2	0.108	0	0	0	0	0	0	0	0	0	0	0	0	0	0	0	0	1	0.108	0.108	288
DNM1	0.107	0	0	0	0	0	0	0	0	0	0	0	0	0	0	0	0	1	0.107	0.107	294
FLNA	0.105	0	0	0	0	0	0	0	0	0	0	0	0	0	0	0	0	1	0.105	0.105	309
PALM	0.105	0	0	0	0	0	0	0	0	0	0	0	0	0	0	0	0	1	0.105	0.105	308
RGS7BP	0.105	0	0	0	0	0	0	0	0	0	0	0	0	0	0	0	0	1	0.105	0.105	305
RAB11FIP1	0.105	0	0	0	0	0	0	0	0	0	0	0	0	0	0	0	0	1	0.105	0.105	306
NECAP2	0.105	0	0	0	0	0	0	0	0	0	0	0	0	0	0	0	0	1	0.105	0.105	310
GNB2	0.103	0	0	0	0	0	0	0	0	0	0	0	0	0	0	0	0	1	0.103	0.103	313
KCNJ5	0.102	0	0	0	0	0	0	0	0	0	0	0	0	0	0	0	0	1	0.102	0.102	316
SAR1B	0.102	0	0	0	0	0	0	0	0	0	0	0	0	0	0	0	0	1	0.102	0.102	315
SLC9A9	0.104	0	0	0	0	0	0	0	0	0	0	0	0	0	0	0	0	1	0.104	0.104	312
GPSM1	0.102	0	0	0	0	0	0	0	0	0	0	0	0	0	0	0	0	1	0.102	0.102	314

Table S5. GPCR concept term interrogation of the LSI-based DDR system corpus. The GPCR-specific concept terms (Table S1) were applied as interrogators to the DDR system corpus created in Table S3. For each specific protein, identified by its official gene symbol, the cosine similarity score (at least >0.1, therefore indicating an implicit association) for the strength of latent semantic association with the GPCR-specific concept terms is given. The sum of the specific concept occurrences, the average and median cosine similarity score and the average rank within the matrix are given for each specific protein.

Gene symbol	G protein	G protein-coupled receptor kinase interacting transcript 2	Regulator of G protein signaling	7 transmembrane receptor	RGS protein	G protein-coupled receptor kinase	GPCR	G protein-coupled receptor	GRK	Beta arrestin	Serpentine receptor	Arrestin	Heptahelical receptor	Heptahelical	Beta-arrestin	GIT2	Sum of occurrences	Average-score	Median score	Average rank
CSTF2	0.446	0.552	0.201	0	0.549	0.286	0.204	0.283	0.229	0	0	0	0.213	0.213	0	0.111	11	0.298818182	0.229	17.81818182
DHX8	0.457	0.509	0.2	0	0	0.182	0.207	0.179	0.191	0.248	0	0.245	0.183	0.183	0.256	0	12	0.253333333	0.2035	16.5
POLR1C	0.463	0.441	0.186	0	0.158	0.135	0.105	0.13	0.113	0	0	0	0	0	0	0	8	0.216375	0.1465	29.625
CSTF3	0.524	0.512	0.249	0.12	0.175	0.105	0	0	0	0	0	0	0	0	0	0	6	0.280833333	0.212	15.83333333
ADIRF	0.409	0.37	0.361	0.156	0	0.121	0.107	0.115	0	0	0	0	0	0	0	0	7	0.234142857	0.156	30.71428571
CNOT11	0.572	0.523	0.314	0.12	0	0	0	0	0	0	0	0	0	0	0	0.103	5	0.3264	0.314	9.8
TP53I13	0.661	0.485	0.361	0.122	0	0	0	0	0	0	0	0	0	0	0	0	4	0.40725	0.423	10.75
POM121C	0.542	0.511	0.304	0.163	0	0	0	0	0	0	0	0	0	0	0	0	4	0.38	0.4075	7
LOC729316	0.542	0.511	0.304	0.163	0	0	0	0	0	0	0	0	0	0	0	0	4	0.38	0.4075	8
PTBP2	0.588	0.482	0.304	0.127	0	0	0	0	0	0	0	0	0	0	0	0	4	0.37525	0.393	12.25
MTERFD3	0.492	0.457	0.308	0.13	0	0	0	0	0	0	0	0	0	0	0	0.102	5	0.2978	0.308	15.6
PRPF4	0.593	0.502	0.254	0.126	0	0	0	0	0	0	0	0	0	0	0	0	4	0.36875	0.378	17.25
PARN	0.523	0.412	0.243	0.137	0	0	0	0	0	0	0.153	0	0	0	0	0	5	0.2936	0.243	25.2
DHX15	0.565	0.521	0.244	0.112	0	0	0	0	0	0	0	0	0	0	0	0	4	0.3605	0.3825	26
MRPL43	0.502	0.459	0.336	0.142	0	0	0	0	0	0	0	0	0	0	0	0	4	0.35975	0.3975	12.75
RPPH1	0.553	0.505	0.245	0.125	0	0	0	0	0	0	0	0	0	0	0	0	4	0.357	0.375	22.75
COA5	0.565	0.405	0.313	0.145	0	0	0	0	0	0	0	0	0	0	0	0	4	0.357	0.359	19.75
SNRNP200	0.454	0.407	0.199	0.105	0	0	0	0	0	0.131	0	0.127	0	0	0	0	6	0.237166667	0.165	48
EIF1	0.536	0.473	0.272	0.123	0	0	0	0	0	0	0	0	0	0	0	0	4	0.351	0.3725	18.75
SRSF1	0.532	0.461	0.261	0.125	0	0	0	0	0	0	0	0	0	0	0	0	4	0.34475	0.361	20.75
H3F3AP4	0.502	0.511	0.248	0.116	0	0	0	0	0	0	0	0	0	0	0	0	4	0.34425	0.375	25.75
HEATR1	0.492	0.447	0.265	0.16	0	0	0	0	0	0	0	0	0	0	0	0	4	0.341	0.356	21.75
CDC5L	0.499	0.404	0.3	0.129	0	0	0	0	0	0	0	0	0	0	0	0	4	0.333	0.352	27.5

LOC401131	0.518	0.354	0.346	0.106	0	0	0	0	0	0	0	0	0	0	0	0	4	0.331	0.35	44.75
UPF2	0.498	0.449	0.262	0.109	0	0	0	0	0	0	0	0	0	0	0	0	4	0.3295	0.3555	30.25
UPF1	0.508	0.439	0.267	0.101	0	0	0	0	0	0	0	0	0	0	0	0	4	0.32875	0.353	32
MTERFD1	0.436	0.423	0.33	0.118	0	0	0	0	0	0	0	0	0	0	0	0	4	0.32675	0.3765	38.75
THOC1	0.529	0.467	0.302	0	0	0	0	0	0	0	0	0	0	0	0	0	3	0.4326666 67	0.467	13.333333 33
PCF11	0.451	0.436	0.278	0.116	0	0	0	0	0	0	0	0	0	0	0	0	4	0.32025	0.357	38.25
BLCAP	0.429	0.402	0.27	0.174	0	0	0	0	0	0	0	0	0	0	0	0	4	0.31875	0.336	44
GTSF1L	0.44	0.477	0.218	0.134	0	0	0	0	0	0	0	0	0	0	0	0	4	0.31725	0.329	50
ZNF331	0.48	0.404	0.254	0.114	0	0	0	0	0	0	0	0	0	0	0	0	4	0.313	0.329	41.25
SET	0.477	0.427	0.24	0	0.104	0	0	0	0	0	0	0	0	0	0	0	4	0.312	0.3335	31.5
ASF1B	0.477	0.378	0.277	0.108	0	0	0	0	0	0	0	0	0	0	0	0	4	0.31	0.3275	44.75
SCARNA2	0.454	0.44	0.23	0.103	0	0	0	0	0	0	0	0	0	0	0	0	4	0.30675	0.335	54.5
SCARNA18	0.475	0.406	0.245	0.101	0	0	0	0	0	0	0	0	0	0	0	0	4	0.30675	0.3255	51.25
SNORA74B	0.475	0.406	0.245	0.101	0	0	0	0	0	0	0	0	0	0	0	0	4	0.30675	0.3255	50.25
SNORA5C	0.475	0.406	0.245	0.101	0	0	0	0	0	0	0	0	0	0	0	0	4	0.30675	0.3255	52.25
SNORA11	0.475	0.406	0.245	0.101	0	0	0	0	0	0	0	0	0	0	0	0	4	0.30675	0.3255	46.25
SNORA81	0.475	0.406	0.245	0.101	0	0	0	0	0	0	0	0	0	0	0	0	4	0.30675	0.3255	47.25
SNORA12	0.475	0.406	0.245	0.101	0	0	0	0	0	0	0	0	0	0	0	0	4	0.30675	0.3255	48.25
SNORA45	0.475	0.406	0.245	0.101	0	0	0	0	0	0	0	0	0	0	0	0	4	0.30675	0.3255	49.25
MED30	0.497	0.422	0.287	0	0	0	0	0	0	0	0	0	0	0	0	0	3	0.402	0.422	24.666666 67
CIZ1	0.416	0.36	0.289	0.128	0	0	0	0	0	0	0	0	0	0	0	0	4	0.29825	0.3245	59.25
HMGA1	0.506	0.395	0.291	0	0	0	0	0	0	0	0	0	0	0	0	0	3	0.3973333 33	0.395	32.666666 67
PUM2	0.475	0.444	0.263	0	0	0	0	0	0	0	0	0	0	0	0	0	3	0.394	0.444	32.333333 33
CHAF1B	0.492	0.418	0.27	0	0	0	0	0	0	0	0	0	0	0	0	0	3	0.3933333 33	0.418	29.333333 33
RRM2B	0.43	0.281	0.321	0.141	0	0	0	0	0	0	0	0	0	0	0	0	4	0.29325	0.301	71.75
CPSF3	0.472	0.449	0.247	0	0	0	0	0	0	0	0	0	0	0	0	0	3	0.3893333 33	0.449	36.333333 33
H3F3B	0.441	0.459	0.267	0	0	0	0	0	0	0	0	0	0	0	0	0	3	0.389	0.441	40.666666 67
TRIT1	0.43	0.377	0.221	0.133	0	0	0	0	0	0	0	0	0	0	0	0	4	0.29025	0.299	68.25
EIF5A2	0.447	0.356	0.246	0.109	0	0	0	0	0	0	0	0	0	0	0	0	4	0.2895	0.301	65.75
DUX4L15	0.405	0.413	0.219	0.118	0	0	0	0	0	0	0	0	0	0	0	0	4	0.28875	0.312	70.5
LSM10	0.494	0.453	0.207	0	0	0	0	0	0	0	0	0	0	0	0	0	3	0.3846666 67	0.453	51.666666 67
SMC2	0.425	0.367	0.239	0.119	0	0	0	0	0	0	0	0	0	0	0	0	4	0.2875	0.303	69.5
TCEA2	0.46	0.457	0.232	0	0	0	0	0	0	0	0	0	0	0	0	0	3	0.383	0.457	48.333333 33
DUX4L14	0.401	0.419	0.204	0.122	0	0	0	0	0	0	0	0	0	0	0	0	4	0.2865	0.3025	74.5

CYP51P2	0.405	0.396	0.194	0.145	0	0	0	0	0	0	0	0	0	0	0	0	4	0.285	0.295	78.5
IFIT1P1	0.383	0.356	0.158	0.136	0.107	0	0	0	0	0	0	0	0	0	0	0	5	0.228	0.158	88.8
TRNT1	0.414	0.331	0.228	0.166	0	0	0	0	0	0	0	0	0	0	0	0	4	0.28475	0.2795	79.5
PAPD4	0.495	0.403	0.232	0	0	0	0	0	0	0	0	0	0	0	0	0	3	0.376666667	0.403	51
SUGP1	0.495	0.414	0.217	0	0	0	0	0	0	0	0	0	0	0	0	0	3	0.375333333	0.414	53
DDX51	0.47	0.409	0.247	0	0	0	0	0	0	0	0	0	0	0	0	0	3	0.375333333	0.409	44.66666667
PRIM2	0.463	0.395	0.158	0.105	0	0	0	0	0	0	0	0	0	0	0	0	4	0.28025	0.2765	90
HIST2H2AA3	0.444	0.44	0.237	0	0	0	0	0	0	0	0	0	0	0	0	0	3	0.373666667	0.44	54.33333333
DDX11	0.343	0.324	0.273	0.171	0	0	0	0	0	0	0	0	0	0	0	0	4	0.27775	0.2985	82.5
SMARCAL1	0.396	0.338	0.24	0.134	0	0	0	0	0	0	0	0	0	0	0	0	4	0.277	0.289	81.5
QSOX1	0.468	0.319	0.196	0.123	0	0	0	0	0	0	0	0	0	0	0	0	4	0.2765	0.2575	88.25
JTB	0.304	0.29	0.188	0	0	0	0	0	0	0	0.11	0	0.105	0.105	0	0	6	0.183666667	0.149	88.66666667
HIST1H4I	0.444	0.417	0.236	0	0	0	0	0	0	0	0	0	0	0	0	0	3	0.365666667	0.417	59.66666667
PIWIL1	0.433	0.401	0.261	0	0	0	0	0	0	0	0	0	0	0	0	0	3	0.365	0.401	59.66666667
FILIP1L	0.426	0.33	0.223	0.116	0	0	0	0	0	0	0	0	0	0	0	0	4	0.27375	0.2765	88
SMARCA1	0.417	0.432	0.243	0	0	0	0	0	0	0	0	0	0	0	0	0	3	0.364	0.417	60.66666667
USB1	0.368	0.334	0.227	0.163	0	0	0	0	0	0	0	0	0	0	0	0	4	0.273	0.2805	90.25
WDHD1	0.46	0.38	0.25	0	0	0	0	0	0	0	0	0	0	0	0	0	3	0.363333333	0.38	57
POLR1A	0.456	0.403	0.228	0	0	0	0	0	0	0	0	0	0	0	0	0	3	0.362333333	0.403	64.66666667
ERI2	0.475	0.36	0.251	0	0	0	0	0	0	0	0	0	0	0	0	0	3	0.362	0.36	56.33333333
EIF3EP1	0.415	0.387	0.168	0.113	0	0	0	0	0	0	0	0	0	0	0	0	4	0.27075	0.2775	96.5
ORC5	0.475	0.386	0.222	0	0	0	0	0	0	0	0	0	0	0	0	0	3	0.361	0.386	67
DCP1B	0.488	0.402	0.189	0	0	0	0	0	0	0	0	0	0	0	0	0	3	0.359666667	0.402	75.33333333
SMARCAD1	0.415	0.412	0.249	0	0	0	0	0	0	0	0	0	0	0	0	0	3	0.358666667	0.412	61.33333333
ASF1A	0.462	0.381	0.232	0	0	0	0	0	0	0	0	0	0	0	0	0	3	0.358333333	0.381	67.66666667
MCMBP	0.467	0.383	0.224	0	0	0	0	0	0	0	0	0	0	0	0	0	3	0.358	0.383	69
GTPBP10	0.475	0.375	0.224	0	0	0	0	0	0	0	0	0	0	0	0	0	3	0.358	0.375	69.33333333
SUPT4H1	0.436	0.423	0.215	0	0	0	0	0	0	0	0	0	0	0	0	0	3	0.358	0.423	70.66666667
RPAP1	0.268	0.248	0	0	0	0	0.109	0	0.103	0.112	0	0.111	0	0	0.121	0	7	0.153142857	0.112	65.42857143
HIST1H1T	0.471	0.389	0.21	0	0	0	0	0	0	0	0	0	0	0	0	0	3	0.356666667	0.389	73.33333333
POLR2A	0.444	0.413	0.212	0	0	0	0	0	0	0	0	0	0	0	0	0	3	0.356333333	0.413	73
PARP8	0.379	0.362	0.215	0.105	0	0	0	0	0	0	0	0	0	0	0	0	4	0.26525	0.2885	96.25
PURA	0.466	0.374	0.218	0	0	0	0	0	0	0	0	0	0	0	0	0	3	0.352666667	0.374	77.33333333

C10ORF2	0.401	0.35	0.179	0.118	0	0	0	0	0	0	0	0	0	0	0	0	4	0.262	0.2645	104.25
POLG2	0.401	0.338	0.156	0.152	0	0	0	0	0	0	0	0	0	0	0	0	4	0.26175	0.247	111.5
RECQL4	0.448	0.37	0.221	0	0	0	0	0	0	0	0	0	0	0	0	0	3	0.346333333	0.37	82
PET112	0.453	0.377	0.208	0	0	0	0	0	0	0	0	0	0	0	0	0	3	0.346	0.377	83.66666667
SUPV3L1	0.458	0.371	0.207	0	0	0	0	0	0	0	0	0	0	0	0	0	3	0.345333333	0.371	84.66666667
HMGN1	0.485	0.329	0.222	0	0	0	0	0	0	0	0	0	0	0	0	0	3	0.345333333	0.329	85.66666667
DHX35	0.445	0.375	0.214	0	0	0	0	0	0	0	0	0	0	0	0	0	3	0.344666667	0.375	84.66666667
POLE2	0.41	0.326	0.185	0	0	0	0	0	0	0	0.111	0	0	0	0	0	4	0.258	0.2555	98.5
NHP2L1	0.467	0.41	0.152	0	0	0	0	0	0	0	0	0	0	0	0	0	3	0.343	0.41	97.33333333
TMEM189-UBE2V1	0.414	0.375	0.236	0	0	0	0	0	0	0	0	0	0	0	0	0	3	0.341666667	0.375	84
ORC1	0.422	0.341	0.26	0	0	0	0	0	0	0	0	0	0	0	0	0	3	0.341	0.341	84.66666667
CENPB	0.434	0.387	0.196	0	0	0	0	0	0	0	0	0	0	0	0	0	3	0.339	0.387	92.66666667
INO80	0.432	0.359	0.225	0	0	0	0	0	0	0	0	0	0	0	0	0	3	0.338666667	0.359	88.33333333
POLE4	0.443	0.357	0.214	0	0	0	0	0	0	0	0	0	0	0	0	0	3	0.338	0.357	94
FANCC	0.362	0.294	0.235	0.121	0	0	0	0	0	0	0	0	0	0	0	0	4	0.253	0.2645	105.75
HIST2H2AA4	0.425	0.42	0.165	0	0	0	0	0	0	0	0	0	0	0	0	0	3	0.336666667	0.42	100.6666667
RPS3	0.449	0.345	0.212	0	0	0	0	0	0	0	0	0	0	0	0	0	3	0.335333333	0.345	96.66666667
HAT1	0.403	0.387	0.215	0	0	0	0	0	0	0	0	0	0	0	0	0	3	0.335	0.387	93.33333333
POLR3GL	0.424	0.354	0.223	0	0	0	0	0	0	0	0	0	0	0	0	0	3	0.333666667	0.354	95.66666667
DHX9	0.448	0.369	0.174	0	0	0	0	0	0	0	0	0	0	0	0	0	3	0.330333333	0.369	105.3333333
MAU2	0.387	0.359	0.242	0	0	0	0	0	0	0	0	0	0	0	0	0	3	0.329333333	0.359	96.33333333
ERI1	0.469	0.321	0.198	0	0	0	0	0	0	0	0	0	0	0	0	0	3	0.329333333	0.321	105.3333333
CTDP1	0.447	0.339	0.201	0	0	0	0	0	0	0	0	0	0	0	0	0	3	0.329	0.339	103.3333333
ORC4	0.43	0.346	0.21	0	0	0	0	0	0	0	0	0	0	0	0	0	3	0.328666667	0.346	104
GMNC	0.404	0.311	0.27	0	0	0	0	0	0	0	0	0	0	0	0	0	3	0.328333333	0.311	97.33333333
HVBS7	0.39	0.355	0.24	0	0	0	0	0	0	0	0	0	0	0	0	0	3	0.328333333	0.355	97.66666667
RAD9B	0.397	0.342	0.242	0	0	0	0	0	0	0	0	0	0	0	0	0	3	0.327	0.342	100
H2AFY	0.38	0.363	0.237	0	0	0	0	0	0	0	0	0	0	0	0	0	3	0.326666667	0.363	98.33333333
AIFM2	0.449	0.315	0.215	0	0	0	0	0	0	0	0	0	0	0	0	0	3	0.326333333	0.315	106.6666667
MCM10	0.436	0.313	0.226	0	0	0	0	0	0	0	0	0	0	0	0	0	3	0.325	0.313	104.6666667
GTF2F2P1	0.312	0.296	0	0	0	0	0	0	0	0.122	0	0.121	0	0	0.123	0	5	0.1948	0.123	75.8
PTTG3P	0.39	0.402	0.181	0	0	0	0	0	0	0	0	0	0	0	0	0	3	0.324333333	0.39	112.3333333

HIST4H4	0.39	0.361	0.222	0	0	0	0	0	0	0	0	0	0	0	0	0	3	0.324333333	0.361	104.333333
TBPL1	0.403	0.367	0.2	0	0	0	0	0	0	0	0	0	0	0	0	0	3	0.323333333	0.367	108
SUPT5H	0.408	0.362	0.2	0	0	0	0	0	0	0	0	0	0	0	0	0	3	0.323333333	0.362	107.666667
SSBP2	0.271	0.282	0.169	0	0	0	0	0	0	0	0.239	0	0	0	0	0	4	0.24025	0.255	145
MAP2K4P1	0.387	0.349	0.224	0	0	0	0	0	0	0	0	0	0	0	0	0	3	0.32	0.349	108.333333
HIST1H2BG	0.423	0.352	0.185	0	0	0	0	0	0	0	0	0	0	0	0	0	3	0.32	0.352	118
SUPT16H	0.416	0.321	0.22	0	0	0	0	0	0	0	0	0	0	0	0	0	3	0.319	0.321	112.333333
PRDM9	0.365	0.383	0.206	0	0	0	0	0	0	0	0	0	0	0	0	0	3	0.318	0.365	111
HIST2H2AC	0.389	0.37	0.192	0	0	0	0	0	0	0	0	0	0	0	0	0	3	0.317	0.37	116
FRA13A	0.332	0.369	0.138	0.11	0	0	0	0	0	0	0	0	0	0	0	0	4	0.23725	0.235	134.75
KHNYN	0.398	0.36	0.191	0	0	0	0	0	0	0	0	0	0	0	0	0	3	0.316333333	0.36	118
FRA1E	0.304	0.339	0.177	0.124	0	0	0	0	0	0	0	0	0	0	0	0	4	0.236	0.2405	128.25
HIST1H2AM	0.39	0.366	0.186	0	0	0	0	0	0	0	0	0	0	0	0	0	3	0.314	0.366	120
GADD45A	0.372	0.294	0.274	0	0	0	0	0	0	0	0	0	0	0	0	0	3	0.313333333	0.294	112.666667
PSMD3	0.395	0.353	0.191	0	0	0	0	0	0	0	0	0	0	0	0	0	3	0.313	0.353	123
UBR2	0.375	0.325	0.238	0	0	0	0	0	0	0	0	0	0	0	0	0	3	0.312666667	0.325	114.666667
POLA2	0.427	0.334	0.17	0	0	0	0	0	0	0	0	0	0	0	0	0	3	0.310333333	0.334	128.666667
BTBD1	0.408	0.353	0.167	0	0	0	0	0	0	0	0	0	0	0	0	0	3	0.309333333	0.353	129.666667
SMC4	0.373	0.312	0.238	0	0	0	0	0	0	0	0	0	0	0	0	0	3	0.307666667	0.312	120
H2AFB2	0.385	0.385	0.151	0	0	0	0	0	0	0	0	0	0	0	0	0	3	0.307	0.385	135
SETX	0.402	0.355	0.162	0	0	0	0	0	0	0	0	0	0	0	0	0	3	0.306333333	0.355	133.333333
MAD2L2	0.385	0.33	0.201	0	0	0	0	0	0	0	0	0	0	0	0	0	3	0.305333333	0.33	128.666667
IK	0.355	0.334	0.224	0	0	0	0	0	0	0	0	0	0	0	0	0	3	0.304333333	0.334	121.666667
NIPBL	0.348	0.343	0.218	0	0	0	0	0	0	0	0	0	0	0	0	0	3	0.303	0.343	125.666667
POLA1	0.423	0.333	0.152	0	0	0	0	0	0	0	0	0	0	0	0	0	3	0.302666667	0.333	142.333333
ACTR8	0.417	0.309	0.177	0	0	0	0	0	0	0	0	0	0	0	0	0	3	0.301	0.309	138
HIST1H2AA	0.372	0.332	0.196	0	0	0	0	0	0	0	0	0	0	0	0	0	3	0.3	0.332	134.666667
PLD6	0.376	0.33	0.193	0	0	0	0	0	0	0	0	0	0	0	0	0	3	0.299666667	0.33	136
NDUFA9P1	0.323	0.304	0.14	0.125	0	0	0	0	0	0	0	0	0	0	0	0	4	0.223	0.222	150
SCARNA23	0.404	0.292	0.196	0	0	0	0	0	0	0	0	0	0	0	0	0	3	0.297333333	0.292	139
HIST1H2BA	0.381	0.345	0.162	0	0	0	0	0	0	0	0	0	0	0	0	0	3	0.296	0.345	144.666667
PARP6	0.354	0.299	0.234	0	0	0	0	0	0	0	0	0	0	0	0	0	3	0.295666667	0.299	131.333333
SUPT6H	0.366	0.348	0.168	0	0	0	0	0	0	0	0	0	0	0	0	0	3	0.294	0.348	143.333333

FRA18C	0.338	0.403	0.136	0	0	0	0	0	0	0	0	0	0	0	0	0	3	0.292333333	0.338	152.6666667
RNASEH2C	0.355	0.344	0.168	0	0	0	0	0	0	0	0	0	0	0	0	0	3	0.289	0.344	147.6666667
RNASEH2A	0.358	0.334	0.172	0	0	0	0	0	0	0	0	0	0	0	0	0	3	0.288	0.334	147.3333333
PARP11	0.331	0.295	0.236	0	0	0	0	0	0	0	0	0	0	0	0	0	3	0.287333333	0.295	138.3333333
FRA9E	0.347	0.341	0.172	0	0	0	0	0	0	0	0	0	0	0	0	0	3	0.286666667	0.341	150
STSP1	0.352	0.374	0.133	0	0	0	0	0	0	0	0	0	0	0	0	0	3	0.286333333	0.352	158.6666667
MCM7	0.382	0.277	0.199	0	0	0	0	0	0	0	0	0	0	0	0	0	3	0.286	0.277	148.3333333
TREX1	0.355	0.309	0.186	0	0	0	0	0	0	0	0	0	0	0	0	0	3	0.283333333	0.309	150.6666667
PARP15	0.337	0.3	0.212	0	0	0	0	0	0	0	0	0	0	0	0	0	3	0.283	0.3	147
SMARCA5	0.328	0.321	0.199	0	0	0	0	0	0	0	0	0	0	0	0	0	3	0.282666667	0.321	149
FAM162B	0.354	0.308	0.186	0	0	0	0	0	0	0	0	0	0	0	0	0	3	0.282666667	0.308	152.6666667
PRIM1	0.397	0.319	0.128	0	0	0	0	0	0	0	0	0	0	0	0	0	3	0.281333333	0.319	168.6666667
C17ORF70	0.322	0.239	0.179	0.103	0	0	0	0	0	0	0	0	0	0	0	0	4	0.21075	0.209	154
CSTF1	0.342	0.344	0.155	0	0	0	0	0	0	0	0	0	0	0	0	0	3	0.280333333	0.342	159.6666667
TPP1	0.324	0.296	0.112	0.106	0	0	0	0	0	0	0	0	0	0	0	0	4	0.2095	0.204	167
APPBP2	0.364	0.294	0.174	0	0	0	0	0	0	0	0	0	0	0	0	0	3	0.277333333	0.294	159.3333333
BAZ1A	0.339	0.315	0.175	0	0	0	0	0	0	0	0	0	0	0	0	0	3	0.276333333	0.315	159.6666667
OR5G1P	0.325	0.35	0.15	0	0	0	0	0	0	0	0	0	0	0	0	0	3	0.275	0.325	170.3333333
MCM6	0.369	0.278	0.172	0	0	0	0	0	0	0	0	0	0	0	0	0	3	0.273	0.278	164
SSRP1	0.376	0.265	0.173	0	0	0	0	0	0	0	0	0	0	0	0	0	3	0.271333333	0.265	165.6666667
RNF168	0.326	0.289	0.197	0	0	0	0	0	0	0	0	0	0	0	0	0	3	0.270666667	0.289	162
ZMAT3	0.349	0.272	0.185	0	0	0	0	0	0	0	0	0	0	0	0	0	3	0.268666667	0.272	165.6666667
RPS6KA2-AS1	0.336	0.273	0.193	0	0	0	0	0	0	0	0	0	0	0	0	0	3	0.267333333	0.273	165
CMTR2	0.347	0.311	0.138	0	0	0	0	0	0	0	0	0	0	0	0	0	3	0.265333333	0.311	182
HUS1B	0.318	0.286	0.185	0	0	0	0	0	0	0	0	0	0	0	0	0	3	0.263	0.286	173
STAG3L3	0.334	0.316	0.135	0	0	0	0	0	0	0	0	0	0	0	0	0	3	0.261666667	0.316	184.3333333
STAG3L2	0.334	0.316	0.135	0	0	0	0	0	0	0	0	0	0	0	0	0	3	0.261666667	0.316	186.3333333
STAG3L4	0.334	0.316	0.135	0	0	0	0	0	0	0	0	0	0	0	0	0	3	0.261666667	0.316	185.3333333
STAG3L1	0.334	0.316	0.135	0	0	0	0	0	0	0	0	0	0	0	0	0	3	0.261666667	0.316	187.3333333
KIN	0.34	0.278	0.162	0	0	0	0	0	0	0	0	0	0	0	0	0	3	0.26	0.278	177.6666667
HNRNPUL1	0.342	0.273	0.164	0	0	0	0	0	0	0	0	0	0	0	0	0	3	0.259666667	0.273	177.6666667
RNF8	0.312	0.28	0.182	0	0	0	0	0	0	0	0	0	0	0	0	0	3	0.258	0.28	177

DHX36	0.341	0.28	0.151	0	0	0	0	0	0	0	0	0	0	0	0	0	3	0.257333333	0.28	183.333333
XRN2	0.315	0.305	0.143	0	0	0	0	0	0	0	0	0	0	0	0	0	3	0.254333333	0.305	193
TIPARP	0.301	0.27	0.192	0	0	0	0	0	0	0	0	0	0	0	0	0	3	0.254333333	0.27	179
HIST1H2BJ	0.352	0.279	0.13	0	0	0	0	0	0	0	0	0	0	0	0	0	3	0.253666667	0.279	193
SSBP1	0.355	0.275	0.126	0	0	0	0	0	0	0	0	0	0	0	0	0	3	0.252	0.275	194.666667
SESN1	0.339	0.189	0.226	0	0	0	0	0	0	0	0	0	0	0	0	0	3	0.251333333	0.226	169
PAPD7	0.283	0.299	0.17	0	0	0	0	0	0	0	0	0	0	0	0	0	3	0.250666667	0.283	184.333333
SMC1A	0.326	0.259	0.163	0	0	0	0	0	0	0	0	0	0	0	0	0	3	0.249333333	0.259	187.333333
RAD21	0.295	0.273	0.176	0	0	0	0	0	0	0	0	0	0	0	0	0	3	0.248	0.273	187.666667
ZCCHC7	0.272	0.299	0.169	0	0	0	0	0	0	0	0	0	0	0	0	0	3	0.246666667	0.272	188
UBL4B	0.322	0.277	0.14	0	0	0	0	0	0	0	0	0	0	0	0	0	3	0.246333333	0.277	199.333333
FANCA	0.314	0.266	0.158	0	0	0	0	0	0	0	0	0	0	0	0	0	3	0.246	0.266	194
MOV10L1	0.292	0.262	0.182	0	0	0	0	0	0	0	0	0	0	0	0	0	3	0.245333333	0.262	188
ANKRD32	0.293	0.281	0.156	0	0	0	0	0	0	0	0	0	0	0	0	0	3	0.243333333	0.281	196
DMRTC2	0.257	0.309	0.163	0	0	0	0	0	0	0	0	0	0	0	0	0	3	0.243	0.257	192.666667
NELFB	0.283	0.294	0.151	0	0	0	0	0	0	0	0	0	0	0	0	0	3	0.242666667	0.283	199
MCM4	0.32	0.239	0.161	0	0	0	0	0	0	0	0	0	0	0	0	0	3	0.24	0.239	198
NCAPG	0.29	0.258	0.166	0	0	0	0	0	0	0	0	0	0	0	0	0	3	0.238	0.258	198.333333
ACTR5	0.32	0.246	0.147	0	0	0	0	0	0	0	0	0	0	0	0	0	3	0.237666667	0.246	205
CDC45	0.3	0.246	0.166	0	0	0	0	0	0	0	0	0	0	0	0	0	3	0.237333333	0.246	197
FANCM	0.312	0.245	0.153	0	0	0	0	0	0	0	0	0	0	0	0	0	3	0.236666667	0.245	201
REV3L	0.322	0.242	0.144	0	0	0	0	0	0	0	0	0	0	0	0	0	3	0.236	0.242	207
PSME4	0.301	0.239	0.168	0	0	0	0	0	0	0	0	0	0	0	0	0	3	0.236	0.239	199.666667
POLM	0.318	0.266	0.122	0	0	0	0	0	0	0	0	0	0	0	0	0	3	0.235333333	0.266	212.333333
ALKBH3	0.328	0.233	0.145	0	0	0	0	0	0	0	0	0	0	0	0	0	3	0.235333333	0.233	208.333333
MCM9	0.311	0.24	0.15	0	0	0	0	0	0	0	0	0	0	0	0	0	3	0.233666667	0.24	206.333333
DBF4B	0.299	0.211	0.189	0	0	0	0	0	0	0	0	0	0	0	0	0	3	0.233	0.211	198
FAN1	0.214	0.187	0.178	0.108	0	0	0	0	0	0	0	0	0	0	0	0	4	0.17175	0.1825	188.25
MCM2	0.31	0.216	0.159	0	0	0	0	0	0	0	0	0	0	0	0	0	3	0.228333333	0.216	209.333333
RECQL	0.325	0.233	0.12	0	0	0	0	0	0	0	0	0	0	0	0	0	3	0.226	0.233	220.333333
TOP1	0.331	0.244	0.103	0	0	0	0	0	0	0	0	0	0	0	0	0	3	0.226	0.244	220.666667
POLE	0.304	0.254	0.119	0	0	0	0	0	0	0	0	0	0	0	0	0	3	0.225666667	0.254	222

GATC	0.309	0.226	0.139	0	0	0	0	0	0	0	0	0	0	0	0	0	3	0.224666667	0.226	220.666667
QRSL1	0.309	0.226	0.139	0	0	0	0	0	0	0	0	0	0	0	0	0	3	0.224666667	0.226	219.666667
ATRX	0.289	0.261	0.121	0	0	0	0	0	0	0	0	0	0	0	0	0	3	0.223666667	0.261	221.666667
FANCI	0.28	0.24	0.149	0	0	0	0	0	0	0	0	0	0	0	0	0	3	0.223	0.24	219.333333
RIF1	0.251	0.266	0.152	0	0	0	0	0	0	0	0	0	0	0	0	0	3	0.223	0.251	214
TP53I3	0.284	0.184	0.2	0	0	0	0	0	0	0	0	0	0	0	0	0	3	0.222666667	0.2	202
NCAPD2	0.279	0.241	0.147	0	0	0	0	0	0	0	0	0	0	0	0	0	3	0.222333333	0.241	219.666667
GINS2	0.291	0.221	0.153	0	0	0	0	0	0	0	0	0	0	0	0	0	3	0.221666667	0.221	215.666667
HNRNPUL2	0.298	0.238	0.123	0	0	0	0	0	0	0	0	0	0	0	0	0	3	0.219666667	0.238	225.666667
UBE2T	0.26	0.236	0.161	0	0	0	0	0	0	0	0	0	0	0	0	0	3	0.219	0.236	215.333333
DDX21	0.319	0.21	0.119	0	0	0	0	0	0	0	0	0	0	0	0	0	3	0.216	0.21	231
CDT1	0.274	0.215	0.158	0	0	0	0	0	0	0	0	0	0	0	0	0	3	0.215666667	0.215	220.333333
NCAPH	0.259	0.226	0.161	0	0	0	0	0	0	0	0	0	0	0	0	0	3	0.215333333	0.226	217.666667
POLQ	0.299	0.233	0.113	0	0	0	0	0	0	0	0	0	0	0	0	0	3	0.215	0.233	232.333333
RPL21P1	0.266	0.211	0.161	0	0	0	0	0	0	0	0	0	0	0	0	0	3	0.212666667	0.211	221
WRNIP1	0.239	0.249	0.149	0	0	0	0	0	0	0	0	0	0	0	0	0	3	0.212333333	0.239	224.666667
RDM1	0.282	0.243	0.111	0	0	0	0	0	0	0	0	0	0	0	0	0	3	0.212	0.243	233.333333
DNA2	0.282	0.235	0.116	0	0	0	0	0	0	0	0	0	0	0	0	0	3	0.211	0.235	235.666667
TOP3B	0.283	0.233	0.116	0	0	0	0	0	0	0	0	0	0	0	0	0	3	0.210666667	0.233	235.333333
CCNO	0.281	0.222	0.121	0	0	0	0	0	0	0	0	0	0	0	0	0	3	0.208	0.222	235.666667
MSH5	0.276	0.24	0.104	0	0	0	0	0	0	0	0	0	0	0	0	0	3	0.206666667	0.24	238.666667
POLE3	0.267	0.232	0.117	0	0	0	0	0	0	0	0	0	0	0	0	0	3	0.205333333	0.232	239.666667
HORMAD1	0.233	0.242	0.13	0	0	0	0	0	0	0	0	0	0	0	0	0	3	0.201666667	0.233	240.333333
SDE2	0.244	0.218	0.132	0	0	0	0	0	0	0	0	0	0	0	0	0	3	0.198	0.218	242.333333
TP53BP1	0.261	0.198	0.119	0	0	0	0	0	0	0	0	0	0	0	0	0	3	0.192666667	0.198	248.333333
RAD9A	0.248	0.184	0.138	0	0	0	0	0	0	0	0	0	0	0	0	0	3	0.19	0.184	247.333333
MCM8	0.245	0.214	0.111	0	0	0	0	0	0	0	0	0	0	0	0	0	3	0.19	0.214	252.666667
FANCG	0.253	0.202	0.113	0	0	0	0	0	0	0	0	0	0	0	0	0	3	0.189333333	0.202	252
MIR5090	0.239	0.179	0.15	0	0	0	0	0	0	0	0	0	0	0	0	0	3	0.189333333	0.179	251.333333
MIR5088	0.239	0.179	0.15	0	0	0	0	0	0	0	0	0	0	0	0	0	3	0.189333333	0.179	250.333333
MIR5087	0.239	0.179	0.15	0	0	0	0	0	0	0	0	0	0	0	0	0	3	0.189333333	0.179	249.333333
MIR5094	0.239	0.179	0.15	0	0	0	0	0	0	0	0	0	0	0	0	0	3	0.189333333	0.179	248.333333

MIR5092	0.239	0.179	0.15	0	0	0	0	0	0	0	0	0	0	0	0	0	3	0.189333333	0.179	247.333333
MIR4511	0.239	0.179	0.15	0	0	0	0	0	0	0	0	0	0	0	0	0	3	0.189333333	0.179	246.333333
MIR5093	0.239	0.179	0.15	0	0	0	0	0	0	0	0	0	0	0	0	0	3	0.189333333	0.179	243.333333
MIR5091	0.239	0.179	0.15	0	0	0	0	0	0	0	0	0	0	0	0	0	3	0.189333333	0.179	245.333333
MIR5089	0.239	0.179	0.15	0	0	0	0	0	0	0	0	0	0	0	0	0	3	0.189333333	0.179	244.333333
C19ORF40	0.257	0.185	0.123	0	0	0	0	0	0	0	0	0	0	0	0	0	3	0.188333333	0.185	250.333333
MIR1206	0.23	0.193	0.136	0	0	0	0	0	0	0	0	0	0	0	0	0	3	0.186333333	0.193	253.333333
DCLRE1B	0.261	0.191	0.102	0	0	0	0	0	0	0	0	0	0	0	0	0	3	0.184666667	0.191	256
TNKS	0.22	0.199	0.121	0	0	0	0	0	0	0	0	0	0	0	0	0	3	0.18	0.199	259
H2AFX	0.246	0.188	0.106	0	0	0	0	0	0	0	0	0	0	0	0	0	3	0.18	0.188	260
RBBP8	0.219	0.206	0.11	0	0	0	0	0	0	0	0	0	0	0	0	0	3	0.178333333	0.206	266
RAD1	0.225	0.188	0.111	0	0	0	0	0	0	0	0	0	0	0	0	0	3	0.174666667	0.188	268.333333
TOPBP1	0.22	0.174	0.128	0	0	0	0	0	0	0	0	0	0	0	0	0	3	0.174	0.174	269
HUS1	0.228	0.173	0.116	0	0	0	0	0	0	0	0	0	0	0	0	0	3	0.172333333	0.173	273.333333
TERF2IP	0.199	0.212	0.102	0	0	0	0	0	0	0	0	0	0	0	0	0	3	0.171	0.199	272.666667
EME2	0.218	0.174	0.118	0	0	0	0	0	0	0	0	0	0	0	0	0	3	0.17	0.174	274.666667
FRA1H	0.186	0.165	0.155	0	0	0	0	0	0	0	0	0	0	0	0	0	3	0.168666667	0.165	265
FANCD2	0.224	0.174	0.107	0	0	0	0	0	0	0	0	0	0	0	0	0	3	0.168333333	0.174	276.666667
NABP2	0.22	0.178	0.103	0	0	0	0	0	0	0	0	0	0	0	0	0	3	0.167	0.178	278.333333
MGME1	0.261	0.231	0	0	0	0	0	0	0	0	0	0	0	0	0	0	2	0.246	0.246	233.5
MCM5	0.227	0.161	0.101	0	0	0	0	0	0	0	0	0	0	0	0	0	3	0.163	0.161	284
POLL	0.254	0.228	0	0	0	0	0	0	0	0	0	0	0	0	0	0	2	0.241	0.241	237.5
POLD1	0.253	0.221	0	0	0	0	0	0	0	0	0	0	0	0	0	0	2	0.237	0.237	241
FRA2G	0.177	0.154	0.141	0	0	0	0	0	0	0	0	0	0	0	0	0	3	0.157333333	0.154	281.666667
REV1	0.25	0.22	0	0	0	0	0	0	0	0	0	0	0	0	0	0	2	0.235	0.235	243
OA23	0.185	0.157	0.12	0	0	0	0	0	0	0	0	0	0	0	0	0	3	0.154	0.157	290.666667
BFHD	0.185	0.157	0.12	0	0	0	0	0	0	0	0	0	0	0	0	0	3	0.154	0.157	288.666667
OA21	0.185	0.157	0.12	0	0	0	0	0	0	0	0	0	0	0	0	0	3	0.154	0.157	287.666667
OA22	0.185	0.157	0.12	0	0	0	0	0	0	0	0	0	0	0	0	0	3	0.154	0.157	289.666667
RPAIN	0.245	0.217	0	0	0	0	0	0	0	0	0	0	0	0	0	0	2	0.231	0.231	245.5
ZNF350	0.172	0.187	0.101	0	0	0	0	0	0	0	0	0	0	0	0	0	3	0.153333333	0.172	285.666667
LIG1	0.238	0.206	0	0	0	0	0	0	0	0	0	0	0	0	0	0	2	0.222	0.222	257.5
MBD4	0.233	0.199	0	0	0	0	0	0	0	0	0	0	0	0	0	0	2	0.216	0.216	261

TONSL	0.258	0.164	0	0	0	0	0	0	0	0	0	0	0	0	0	0	2	0.211	0.211	269
POLI	0.223	0.198	0	0	0	0	0	0	0	0	0	0	0	0	0	0	2	0.2105	0.2105	266.5
WRN	0.237	0.184	0	0	0	0	0	0	0	0	0	0	0	0	0	0	2	0.2105	0.2105	268
POLK	0.223	0.189	0	0	0	0	0	0	0	0	0	0	0	0	0	0	2	0.206	0.206	270.5
TERF1	0.215	0.197	0	0	0	0	0	0	0	0	0	0	0	0	0	0	2	0.206	0.206	271.5
RPA2	0.244	0.163	0	0	0	0	0	0	0	0	0	0	0	0	0	0	2	0.2035	0.2035	277
SETMAR	0.235	0.171	0	0	0	0	0	0	0	0	0	0	0	0	0	0	2	0.203	0.203	279
TREX2	0.232	0.174	0	0	0	0	0	0	0	0	0	0	0	0	0	0	2	0.203	0.203	277.5
TDP1	0.239	0.166	0	0	0	0	0	0	0	0	0	0	0	0	0	0	2	0.2025	0.2025	280
CTC1	0.201	0.199	0	0	0	0	0	0	0	0	0	0	0	0	0	0	2	0.2	0.2	276
PARP9	0.164	0.131	0.103	0	0	0	0	0	0	0	0	0	0	0	0	0	3	0.132666667	0.131	304.6666667
PIF1	0.201	0.187	0	0	0	0	0	0	0	0	0	0	0	0	0	0	2	0.194	0.194	282
INTS3	0.216	0.169	0	0	0	0	0	0	0	0	0	0	0	0	0	0	2	0.1925	0.1925	289
TERF2	0.206	0.179	0	0	0	0	0	0	0	0	0	0	0	0	0	0	2	0.1925	0.1925	287.5
RMI2	0.212	0.172	0	0	0	0	0	0	0	0	0	0	0	0	0	0	2	0.192	0.192	289
TINF2	0.19	0.194	0	0	0	0	0	0	0	0	0	0	0	0	0	0	2	0.192	0.192	281
MMS22L	0.227	0.152	0	0	0	0	0	0	0	0	0	0	0	0	0	0	2	0.1895	0.1895	294.5
RPA3	0.212	0.166	0	0	0	0	0	0	0	0	0	0	0	0	0	0	2	0.189	0.189	292.5
APTX	0.206	0.167	0	0	0	0	0	0	0	0	0	0	0	0	0	0	2	0.1865	0.1865	293
TOP3A	0.206	0.164	0	0	0	0	0	0	0	0	0	0	0	0	0	0	2	0.185	0.185	297
GEN1	0.208	0.152	0	0	0	0	0	0	0	0	0	0	0	0	0	0	2	0.18	0.18	302.5
INIP	0.202	0.157	0	0	0	0	0	0	0	0	0	0	0	0	0	0	2	0.1795	0.1795	301
PURG	0.184	0.175	0	0	0	0	0	0	0	0	0	0	0	0	0	0	2	0.1795	0.1795	298
MSH4	0.192	0.164	0	0	0	0	0	0	0	0	0	0	0	0	0	0	2	0.178	0.178	301
WRAP53	0.195	0.16	0	0	0	0	0	0	0	0	0	0	0	0	0	0	2	0.1775	0.1775	301.5
RPA1	0.206	0.146	0	0	0	0	0	0	0	0	0	0	0	0	0	0	2	0.176	0.176	304.5
RAD54L	0.212	0.139	0	0	0	0	0	0	0	0	0	0	0	0	0	0	2	0.1755	0.1755	303
GADD45AP1	0.233	0.116	0	0	0	0	0	0	0	0	0	0	0	0	0	0	2	0.1745	0.1745	297.5
POLN	0.189	0.158	0	0	0	0	0	0	0	0	0	0	0	0	0	0	2	0.1735	0.1735	305
NABP1	0.185	0.154	0	0	0	0	0	0	0	0	0	0	0	0	0	0	2	0.1695	0.1695	310
DCLRE1A	0.186	0.153	0	0	0	0	0	0	0	0	0	0	0	0	0	0	2	0.1695	0.1695	310
OBFC1	0.164	0.17	0	0	0	0	0	0	0	0	0	0	0	0	0	0	2	0.167	0.167	306.5
MUS81	0.195	0.134	0	0	0	0	0	0	0	0	0	0	0	0	0	0	2	0.1645	0.1645	311
COA1	0.191	0.136	0	0	0	0	0	0	0	0	0	0	0	0	0	0	2	0.1635	0.1635	311.5

BRIP1	0.167	0.159	0	0	0	0	0	0	0	0	0	0	0	0	0	0	2	0.163	0.163	311
PNKP	0.17	0.137	0	0	0	0	0	0	0	0	0	0	0	0	0	0	2	0.1535	0.1535	318
RAD50	0.174	0.13	0	0	0	0	0	0	0	0	0	0	0	0	0	0	2	0.152	0.152	319
FRA2H	0.119	0.171	0	0	0	0	0	0	0	0	0	0	0	0	0	0	2	0.145	0.145	311
BLM	0.162	0.128	0	0	0	0	0	0	0	0	0	0	0	0	0	0	2	0.145	0.145	323.5
EME1	0.157	0.125	0	0	0	0	0	0	0	0	0	0	0	0	0	0	2	0.141	0.141	325
FEN1	0.142	0.14	0	0	0	0	0	0	0	0	0	0	0	0	0	0	2	0.141	0.141	322
MRE11A	0.165	0.11	0	0	0	0	0	0	0	0	0	0	0	0	0	0	2	0.1375	0.1375	324.5
EXO1	0.159	0.108	0	0	0	0	0	0	0	0	0	0	0	0	0	0	2	0.1335	0.1335	327
RAD54B	0.137	0.114	0	0	0	0	0	0	0	0	0	0	0	0	0	0	2	0.1255	0.1255	328
RMI1	0.137	0.102	0	0	0	0	0	0	0	0	0	0	0	0	0	0	2	0.1195	0.1195	330
POT1	0.114	0.118	0	0	0	0	0	0	0	0	0	0	0	0	0	0	2	0.116	0.116	329.5
DCLRE1C	0.165	0	0	0	0	0	0	0	0	0	0	0	0	0	0	0	1	0.165	0.165	320
NBN	0.153	0	0	0	0	0	0	0	0	0	0	0	0	0	0	0	1	0.153	0.153	327
ATMIN	0.128	0	0	0	0	0	0	0	0	0	0	0	0	0	0	0	1	0.128	0.128	331
IGHV3-7	0.123	0	0	0	0	0	0	0	0	0	0	0	0	0	0	0	1	0.123	0.123	332
EXO5	0.11	0	0	0	0	0	0	0	0	0	0	0	0	0	0	0	1	0.11	0.11	335
ATM	0.101	0	0	0	0	0	0	0	0	0	0	0	0	0	0	0	1	0.101	0.101	336

Table S6. GPCR-associated factors from the LSI-generated GPCR-DDR interaction cloud. For each specific GPCR system protein identified in the interaction cloud, the protein description, official gene symbol and cumulate cosine similarity score (across the specific concept interrogators) are given.

Protein Description	Gene Symbol	Cumulative Cosine Similarity Score
Olfactory receptor 6V1	OR6V1	1.165
Brefeldin A-inhibited guanine nucleotide-exchange protein 1	ARFGEF1	0.963
Arrestin domain-containing protein 4	ARRDC4	0.364
Adhesion G protein-coupled receptor L2	LPHN2	0.351
Vomeronasal type-1 receptor 1	VN1R1	0.35
Regulator of G-protein signaling 6	RGS6	0.314
Guanine nucleotide-binding protein G(I)/G(S)/G(O) subunit gamma-12	GNG12	0.243
Histamine H4 receptor	HRH4	0.16
Atypical chemokine receptor 2	ACKR2	0.154
ER lumen protein-retaining receptor 3	KDELR3	0.15
Hydroxycarboxylic acid receptor 1	HCAR1	0.135
ADP-ribosylation factor GTPase-activating protein 2	ARFGAP2	0.125
Secretin receptor	SCTR	0.124
Neurotensin receptor type 2	NTSR2	0.117
Free fatty acid receptor 1	FFAR1	0.109
Receptor expression-enhancing protein 2	REEP2	0.108
Adenylate cyclase type 3	ADCY3	0.106
G protein-coupled receptor kinase 6 pseudogene 1	GRK6P1	0.101
Relaxin-3 receptor 1	RXFP3	0.101
Galanin receptor type 3	GALR3	0.1

Table S7. DDR-associated factors from the LSI-generated GPCR-DDR interaction cloud. For each specific DDR system protein identified in the interaction cloud, the protein description, official gene symbol and cumulate cosine similarity score (across the specific concept interrogators) are given.

**Protein Description**	**Gene Symbol**	**Cumulative Cosine Similarity Score**
Mitochondrial transcription termination factor 2	MTERFD3	1.489
Poly(ADP-ribose) polymerase family member 8	PARP8	1.061
Ring finger protein 8	RNF8	0.774
Single stranded DNA binding protein 1	SSBP1	0.756
FA complementation group A	FANCA	0.738
DNA topoisomerase I	TOP1	0.678
Replication timing regulatory factor 1	RIF1	0.669
Werner helicase interacting protein 1	WRNIP1	0.637
H2A histone family member X	H2AFX	0.54
TERF2 interacting protein	TERF2IP	0.513
Werner syndrome RecQ like helicase	WRN	0.421
Telomeric repeat binding factor 2	TERF2	0.385
Aprataxin	APTX	0.373
Growth arrest and DNA damage inducible alpha pseudogene 1	GADD45AP1	0.349
BRCA1 interacting protein C-terminal helicase 1	BRIP1	0.326
RAD50 double strand break repair protein	RAD50	0.304
Bloom syndrome RecQ like helicase	BLM	0.29
MRE11 homolog, double strand break repair nuclease	MRE11A	0.275
ATM interactor	ATMIN	0.128
ATM serine/threonine kinase	ATM	0.101

Table S8. Ingenuity signaling pathway analysis of the combined GPCR and DDR system LSI-based lists. For each specific Ingenuity canonical signaling pathway significantly populated by the combined GPCR and DDR LSI protein lists (Table S4, S5), the negative $\log_{10}$ of the *p*-value, the enrichment ratio and the specific pathway-associated proteins from the input dataset are indicated.

Ingenuity Canonical Pathways	–log(*p*-value)	Ratio	Pathway-Associated Proteins
Cell Cycle Control of Chromosomal Replication	22.8	0.411	MCM5,LIG1,MCM6,CDC45,MCM8,CDT1,POLA1,POLE,DNA2,RPA1,POLD1,MCM4,RPA2,RPA3,PRIM1,MCM2,ORC5,POLA2,PRIM2,MCM9,ORC4,MCM7,ORC1
ATM Signaling	9.17	0.163	RNF8,TOPBP1,RBBP8,TDP1,RAD50,SMC1A,PPP1CC,RNF168,GADD45A,FANCD2,SMC2,RAD9A,PTPA,H2AFX,TP53BP1,BLM
Role of BRCA1 in DNA Damage Response	8.51	0.175	FANCM,FAAP100,TOPBP1,FANCG,RBBP8,RPA1,FANCC,RAD50,GADD45A,FANCD2,FAAP24,BRIP1,BLM,FANCA
Telomere Extension by Telomerase	7.78	0.467	TERF2,TNKS,TERF2IP,TINF2,TERF1,POT1,RAD50
Cardiac β-Adrenergic Signaling	6.85	0.113	PDE2A,PDE9A,ADCY3,PPP1CB,AKAP6,GNG3,PLD6,PDE1A,AKAP11,GNG10,PDE1C,PDE8A,PPP1CC,PTPA,PDE11A,GNG12
DNA Double-Strand Break Repair by Homologous Recombination	6.46	0.429	LIG1,GEN1,POLA1,ATRX,RPA1,RAD50
Hereditary Breast Cancer Signaling	5.06	0.0933	FANCM,FAAP100,FANCG,POLR2J,RPA1,FANCC,RAD50,POLR2A,GADD45A,FANCD2,H2AFX,FAAP24,BLM,FANCA
Relaxin Signaling	4.8	0.0886	PDE2A,PDE9A,ADCY3,RLN2,GNG3,PLD6,PDE1A,GNG10,PDE1C,PDE8A,RLN1,RXFP1,PDE11A,GNG12
tRNA Splicing	4.39	0.167	PDE8A,PDE2A,PDE9A,PLD6,PDE1A,PDE11A,PDE1C
Pyrimidine Ribonucleotides Interconversion	4.19	0.156	SMARCA1,SMARCAL1,RECQL4,RAD54L,BLM,RECQL,DHX9
Pyrimidine Ribonucleotides De Novo Biosynthesis	4.07	0.149	SMARCA1,SMARCAL1,RECQL4,RAD54L,BLM,RECQL,DHX9
Cleavage and Polyadenylation of Pre-mRNA	3.95	0.333	CSTF1,CSTF2,CPSF3,CSTF3
BER Pathway	3.95	0.333	LIG1,PNKP,POLE,FEN1
Mismatch Repair in Eukaryotes	3.41	0.25	RPA1,FEN1,POLD1,EXO1
Retinoic Acid-Mediated Apoptosis Signaling	3.3	0.113	PARP6,TNKS,PARP15,TIPARP,PARP8,PARP11,PARP9
L-Glutamine Biosynthesis II (tRNA-Dependent)	3.29	1	GATB,QRSL1
Nucleotide Excision Repair Pathway	2.96	0.143	RPA3,POLR2A,POLR2J,RPA1,RPA2
Death Receptor Signaling	2.9	0.086	PARP6,TNKS,PARP15,TIPARP,PARP8,PARP11,MAP3K5,PARP9
Transcriptional Regulatory Network in Embryonic Stem Cells	2.86	0.111	SET,RIF1,H3F3A/H3F3B,HIST4H4,HIST1H4I,SMARCAD1
Role of CHK Proteins in Cell Cycle Checkpoint Control	2.74	0.105	PTPA,RAD9A,HUS1,RPA1,RAD50,RAD1
Protein Kinase A Signaling	2.65	0.0474	PDE2A,PDE9A,ADCY3,PPP1CB,AKAP6,PYGB,GNG3,PLD6,PDE1A,AKAP11,PDE1C,GNG10,PDE8A,PPP1CC,H3F3A/H3F3B,HIST1H1T,PDE11A,GNG12,DUSP16
DNA Damage-Induced 14-3-3σ Signaling	2.06	0.158	RAD9A,HUS1,RAD1
Gustation Pathway	2.06	0.0584	PDE8A,PDE2A,LPAR4,PDE9A,ADCY3,PLD6,PDE1A,PDE11A,PDE1C
UVA-Induced MAPK Signaling	1.85	0.0625	PARP6,TNKS,PARP15,TIPARP,PARP8,PARP11,PARP9

Telomerase Signaling	1.76	0.0598	TERF2,PTPA,TERF2IP,TPP1,TINF2,TERF1,POT1
3-Phosphoinositide Degradation	1.55	0.0506	MTMR6,INPP4A,SET,PPP1CC,PTPA,ILKAP,PPP5C,DUSP16
Assembly of RNA Polymerase I Complex	1.53	0.167	POLR1C,POLR1A
G Protein Signaling Mediated by Tubby	1.45	0.0938	GNG3,GNG12,GNG10
cAMP-Mediated Signaling	1.44	0.0439	PDE8A,PDE2A,PDE9A,ADCY3,AKAP6,PLD6,PDE1A,PDE11A,AKAP11,PDE1C
DNA Double-Strand Break Repair by Non-Homologous End Joining	1.41	0.143	WRN,RAD50
DNA Methylation and Transcriptional Repression Signaling	1.38	0.0882	H3F3A/H3F3B,HIST4H4,HIST1H4I
D-myo-inositol (1,4,5,6)-tetrakisphosphate Biosynthesis	1.33	0.0486	MTMR6,SET,PPP1CC,PTPA,ILKAP,PPP5C,DUSP16
D-myo-inositol (3,4,5,6)-tetrakisphosphate Biosynthesis	1.33	0.0486	MTMR6,SET,PPP1CC,PTPA,ILKAP,PPP5C,DUSP16
